# Hydrogen-Bonded Thiol Undergoes Unconventional Excited-State Intramolecular Proton-Transfer Reactions

**DOI:** 10.1021/jacs.3c10405

**Published:** 2024-01-30

**Authors:** Jian-Kai Wang, Chih-Hsing Wang, Chi-Chi Wu, Kai-Hsin Chang, Chun-Hsiang Wang, Yi-Hung Liu, Chao-Tsen Chen, Pi-Tai Chou

**Affiliations:** † Department of Chemistry, National Taiwan University, Taipei 10617, Taiwan, Republic of China; ‡ Center for Emerging Material and Advanced Devices, National Taiwan University, Taipei 10617, Taiwan, Republic of China

## Abstract

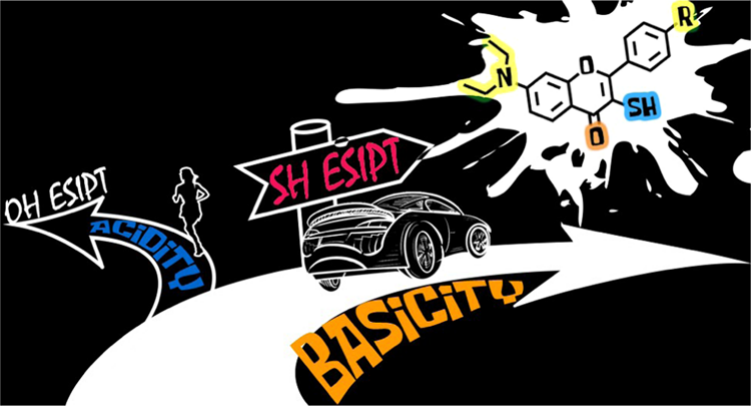

The chapter on the thiol-related hydrogen bond (H-bond) and its excited-state intramolecular proton-transfer (ESIPT) reaction was recently opened where compound 4′-diethylamino-3-mercaptoflavone (**3NTF**) undergoes ESIPT in both cyclohexane solution and solid, giving a 710 nm tautomer emission with an anomalously large Stokes shift of 12,230 cm^–1^. Considering the thiol H-bond to be unconventional compared to the conventional Pauling-type –OH or –NH H-bond, it is thus essential and timely to probe its fundamental difference between their ESIPT. However, thiol-associated ESIPT tends to be nonemissive due to the dominant *n*π* character of the tautomeric lowest excited state. Herein, based on the 3-mercaptoflavone scaffold and π-elongation concept, a new series of 4′-substituted-7-diethylamino-3-mercaptoflavones, **NTFs**, was designed and synthesized with varied H-bond strength and 690–720 nm tautomeric emission upon ultraviolet (UV) excitation in cyclohexane. The order of their H-bonding strength was experimentally determined to be **N-NTF** < **O-NTF** < **H-NTF** < **F-NTF**, while the rate of –SH ESIPT measured by fluorescence upconversion was **F-NTF** (398 fs)^−1^ < **H-NTF** (232 fs)^−1^ < **O-NTF** (123 fs)^−1^ < **N-NTF** (101 fs)^−1^ in toluene. Unexpectedly, the strongest H-bonded **F-NTF** gives the slowest ESIPT, which does not conform to the traditional ESIPT model. The results are rationalized by the trend of carbonyl oxygen basicity rather than –SH acidity. Namely, the thiol acidity relevant to the H-bond strength plays a minor role in the driving force of ESIPT. Instead, the proton-accepting strength governs ESIPT. That is to say, the noncanonical thiol H-bonding system undergoes an unconventional type of ESIPT.

## Introduction

The interactions of high electronegativity fluorine, oxygen, nitrogen, and their associated hydrogen atom, dubbed as the conventional Pauling-type hydrogen bond (H-bond),^1,2^ have been dominating the H-bond relevant research field. In comparison, the –SH H-bond may virtually be considered a “non-Pauling-type” H-bond because of the involvement of a weakly electronegative sulfur atom. The –SH H-bonding system had not been in the center of the spotlight or the main participants in the H-bond relevant research. The early conventional case that kept sulfur out of the scope was based on the observation of boiling points of hydrogen chalcogenides including H_2_S, where only water is exceptionally polarized of all, demonstrating an abnormally high boiling point, and was rationalized with the existence of a strong intermolecular H-bond. Thus, H_2_S, with other hydrogen chalcogenides, was classified as the non-hydrogen bond species; earlier, only a few literature studies had validated the role of sulfur in the H-bond.^3−5^ Yet, with the advances of spectroscopic methods and theoretical calculations, the perspective that sulfur participates in the H-bond was deemed to be acceptable and further came to the fore.^6−10^ For instance, in biological systems, the thioether in methionine-containing dipeptides exhibited amide–NH---S hydrogen bonds, which were even stronger than the amide–NH---O=C version.^10^ Cysteine-containing proteins also adopted –SH---O=C-type hydrogen bonds to stabilize their secondary structures.^11^ Sulfur-mediated H-bonds have also been applied to artificial nucleic acid research^12^ and protein engineering.^13^ Notwithstanding the pervasiveness of sulfur-mediated hydrogen bonds, the lack of fundamental photophysical studies made them less understanding and hence application.

As for the H-bond-related research, its associated proton transfer in the electronically excited states has long been an appealing field.^14−18^ Of particular interest is the excited-state intramolecular proton transfer (ESIPT).^19−30^ In most cases, the existence of an intramolecular H-bond in the ground state is the prerequisite for ESIPT. Upon photoexcitation, enhanced acidification of proton donors, typically –OH or –NH, or basification of proton acceptors, =O or N, synergistically makes viable proton transfer through an intramolecular H-bond, forming a proton-transfer tautomer. The resulting tautomer, in most cases, is capable of emitting photons with an exceptionally large Stokes shift. Such a photophysical reaction can be further fine-tuned by the H-bond nature and can generate different types of proton-transfer properties, such as adiabatic- versus nonadiabatic-type ESIPT^31−36^ or kinetic- versus thermodynamic-type ESIPT,^33^ which has become an advantageous strategy in the design of functional materials^37−49^ and analytical applications.^50−61^

**Figure 1:**
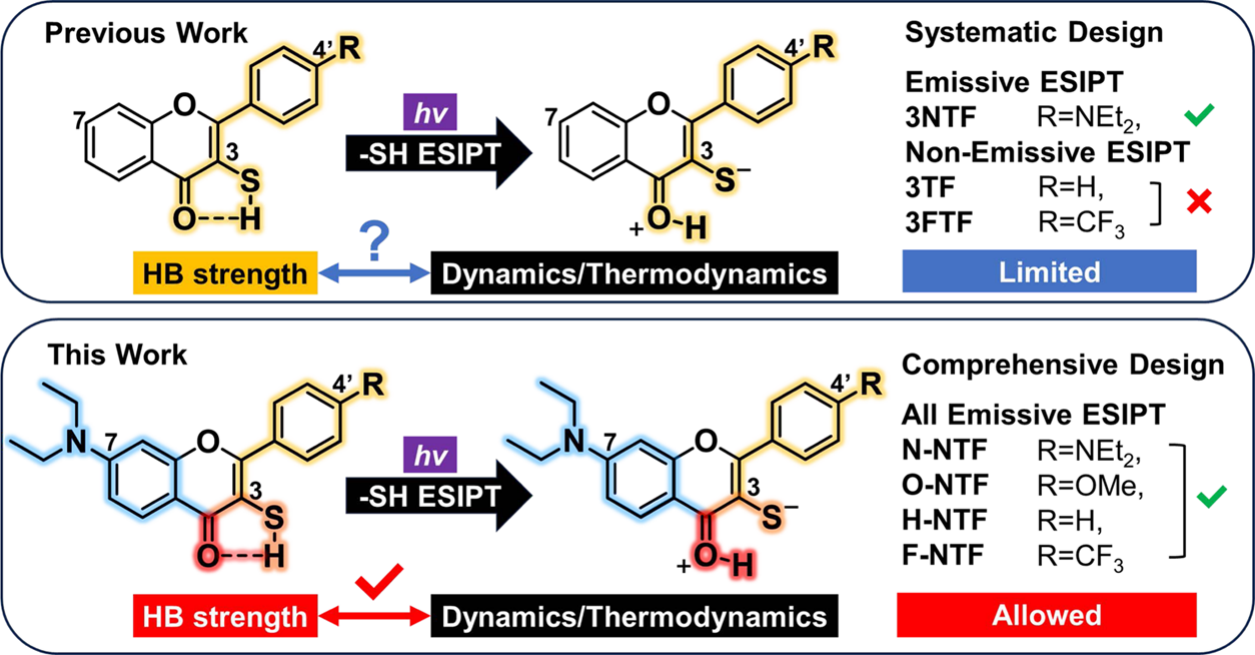
Progression from **3NTF**, **3TF**, and **3FTF** to **NTFs**, i.e., **H-NTF, N-NTF, O-NTF**, and **F-NTF**. The yellow and blue highlights indicate the substituent effect on the carbonyl (red) and thiol groups (orange), respectively, through the resonance or inductive effect.

On the other hand, sulfur has a larger atomic size (cf. O and N), low electronegativity, and hence a great dispersed valence electron cloud. This commonly leads to the more acidic property of the –SH hydrogen (cf., the –OH hydrogen). For example, the p*K*_a_ of phenol is 9.99, whereas it is 6.50 for benzenethiol.^62^ Also, while the conventional H-bond may be realized as a monopole–dipole static interaction between the positively charged H atom, in part, and the dipolar part of the accepting site, the thiol H-bond may be more subject to a dispersive type of interaction. Accordingly, whether a thiol-mediated H-bond and its associated ESIPT are distinct from classical –OH (–NH)-type ESIPT is one of the central issues in H-bonding chemical science. Two of the most relevant cases are the proton transfer of thiotropolone^63,64^ and β-thiooxoketones^7,65,66^ in which the hydrogen-bonded thione-hydroxyl undergoes –OH proton-transfer reactions, generating a thiol–carbonyl tautomer. Both of these cases, though the sulfur element is involved, should not be considered as the –SH-type ESIPT. Until recently, via the synthesis of 3-mercaptoflavones **3NTF**, **3FTF**, and **3TF** (Figure 1), we reported an unambiguous case of thiol H-bond-associated ESIPT, among which **3NTF** showed a prominent 710 nm tautomer emission at room temperature with a large Stokes shift of 12,230 cm^–1^ in cyclohexane.^67^ Our report is the first one involving the ESIPT process in a thiol-based flavone system. The study opens a new chapter in the field of ESIPT for unconventional, i.e., the non-Pauling-type, H-bonding systems. Further extension to gain in-depth and broad insights into thiol-ESIPT is required, among which the structure–thiol-ESIPT relationship is of key interest. It is of fundamental importance to probe the correlation for H-bonding strength versus thiol-ESIPT dynamics/thermodynamics that has been widely studied in the –OH- and –NH-type ESIPT systems (vide infra).^35,36,68,69^ Unfortunately, limited by only **3NTF** exhibiting tautomer emission, further research was pending.^67^

Bearing the goal of shedding light on the correlation between thiol H-bonding and ESIPT properties, we report here the strategy and synthesis of a new series of 3-mercaptoflavones, in which the underlying design concept is based on the experimental demand and chemical logistics: (1) Upon forming a zwitterionic tautomer after ESIPT, if there is any (Figure 1), the energy of the sulfur nonbonding orbital (*n*) should increase significantly, lowering the corresponding nπ* transition, which accounts for the nonemissive tautomer for **3TF** and **3FTF** in previous work (Figure 1).^67^ To overcome this hurdle, in this study, a diethylamino group, N(C_2_H_5_)_2_, is incorporated at the C(7) position to elongate the π-conjugation and hence lower the ππ* transition to ensure the observation of tautomer emission. (2) Accordingly, the C(4′) position, which is facile for chemical modification, can be functionalized by substituents with different electronic properties. As a result, compounds **H-NTF**, **N-NTF**, **O-NTF**, and **F-NTF** (Figure 1) were synthesized, which greatly extended the chemical diversity in terms of H-bonding strength (vide infra). Note that hereafter, we use **NTFs** as a generic name to represent the sum of the titled compounds in this study. With these, the insight of structure versus thiol-ESIPT can be probed in a comprehensive manner. Details of the results and discussion are elaborated in the following sections.

## Results and Discussion

### Synthesis and Characterization

**Scheme 1:**
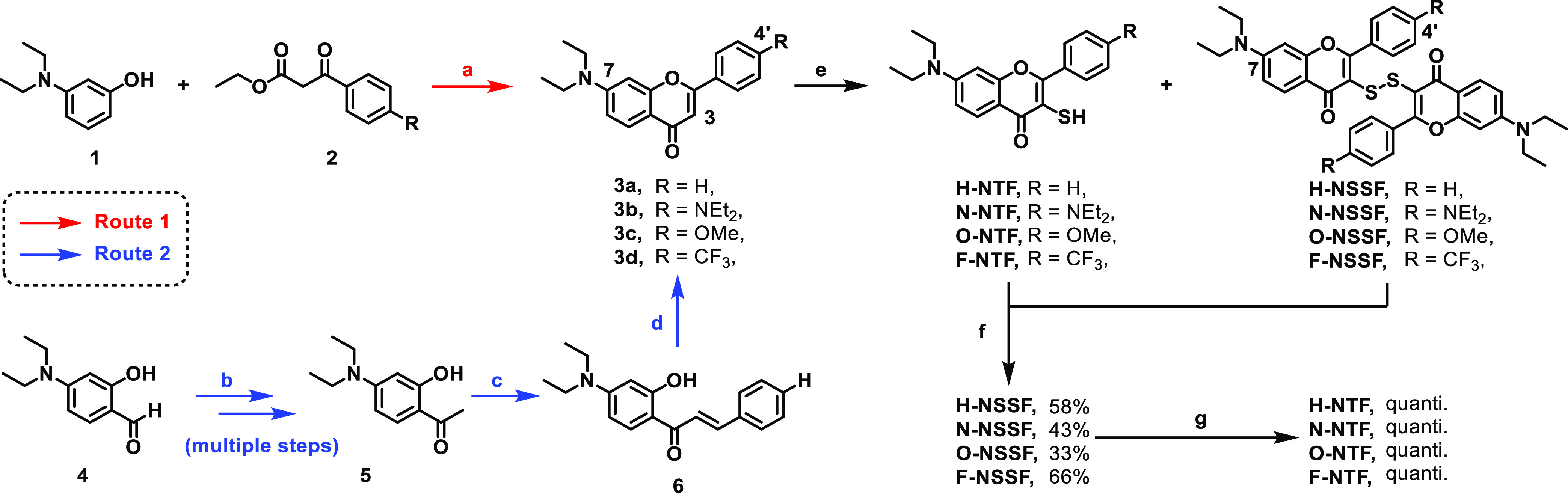
Syntheses of **H-NTF**, **N-NTF**, **O-NTF**, and **F-NTF** Reagents and conditions: (a) neat, 100 °C, 5 h; then 180 °C, 56 h, **3a** (25%), **3b** (51%), **3c** (46%), and **3d** (48%); (b) (i) KOH, K_2_CO_3_, MeCN, reflux, 10 min; then MeI, reflux, 2 h, 85%, (ii) MeMgBr, anhyd. THF, 0 °C to rt, 1 h, (iii) DDQ, 1,4-dioxane, r.t., 4 h, 94% (over two steps), (iv) BBr3, anhyd. DCM, 0 °C 10 min, then r.t., 2 h, 38%; (c) benzaldehyde, ^*t*^BuOK, benzene, 80 °C, 3 h, 81%; (d) I_2_, DMSO, 160 °C, 1 h, 43%; (e) (i) freshly prep. LDA, THF, −78 °C, 30 min, (ii) S8, −78 °C, 1 h, (iii) 10% HCl_(aq)_, **H-NTF/H-NSSF** = 2:1 based on ^1^H NMR (see Figure S1); (f) NEt_3_, I_2_, MeOH, rt, 30 min, 58% (over two steps); and (g) NaBH_4_ (3 equiv), EtOH/CHCl_3_, 0 °C, 1 h; then citric acid (5 equiv) 30 min, quantitative.

Syntheses of **NTFs** were achieved by, first, construction of flavone scaffolds, followed by the introduction of sulfur to afford the desired mercaptoflavone derivatives. Intuitively, using the previously reported synthetic route from our group to synthesize the flavone molecular framework was first attempted (Scheme 1). Starting with commercially available 4-diethylamino-2-hydroxybenzaldehyde (**4**), multiple steps involving methylation of the hydroxyl group followed by the addition of the methyl Grignard reagent, oxidation of the resulting secondary alcohol, and demethylation promoted by boron tribromide (BBr_3_) furnished 4-diethylamino-2-hydroxyacetophenone (**5**).^70^ Subsequent Claisen–Schmidt condensation of **5** with benzaldehyde followed by an iodine-mediated oxidation reaction yielded flavone **3a**. Despite the fact that the desired flavone can be obtained, the lengthy syntheses resulted in a relatively poor total yield of **3a** (ca. 11%). Alternatively, a more concise synthetic route recently reported by Gamsey et al. was adopted to prepare **3a**–**d** (Scheme 1).^71^ Starting with commercially available 3-diethylaminophenol (**1**) and various 1-ethoxy-3-(4′-substituted phenyl)propane-1,3-dione (**2**), the flavone scaffold can be readily synthesized in a one-pot manner with moderate to good yields (25–51%). With **3a**–**d** in hand, **H-NTF**, **N-NTF**, **O-NTF**, and **F-NTF** were synthesized following previously reported procedures. A lithium diisopropylamide (LDA)-mediated lithiation followed by the addition of sulfur powder resulted in a mixture of **NTFs** and oxidized **NSSF** dimers (Scheme 1). Oxidization of the mixture with iodine in the presence of triethylamine (TEA) and then reduction with NaBH_4_ followed by acidification with citric acid and sublimation at 200 °C under a reduced pressure of 10 Pa gave pure **NTFs**.^67^

To characterize –SH H-bonding features of the newly synthesized **NTFs**, Fourier transform infrared (FTIR) experiments were first carried out, where broad and weak S–H stretching frequencies were resolved at 2436, 2478, 2477, and 2472 cm^–1^ for **F-NTF, H-NTF**, **O-NTF**, and **N-NTF**, respectively, in the pure solid film on KBr (see Figure S46). These results were all comparable to our previously reported **3NTF** (2493 cm^–1^) and also lower in frequency than that of free S–H stretching predicted at ∼2600 to 2550 cm^–1^, firmly supporting the existence of intramolecular hydrogen bonds.^72−75^ Furthermore, **F-NTF** displays the most unique low stretching frequency and the broadest peak shape, hinting at the strongest intramolecular H-bonding nature.

**Figure 2:**
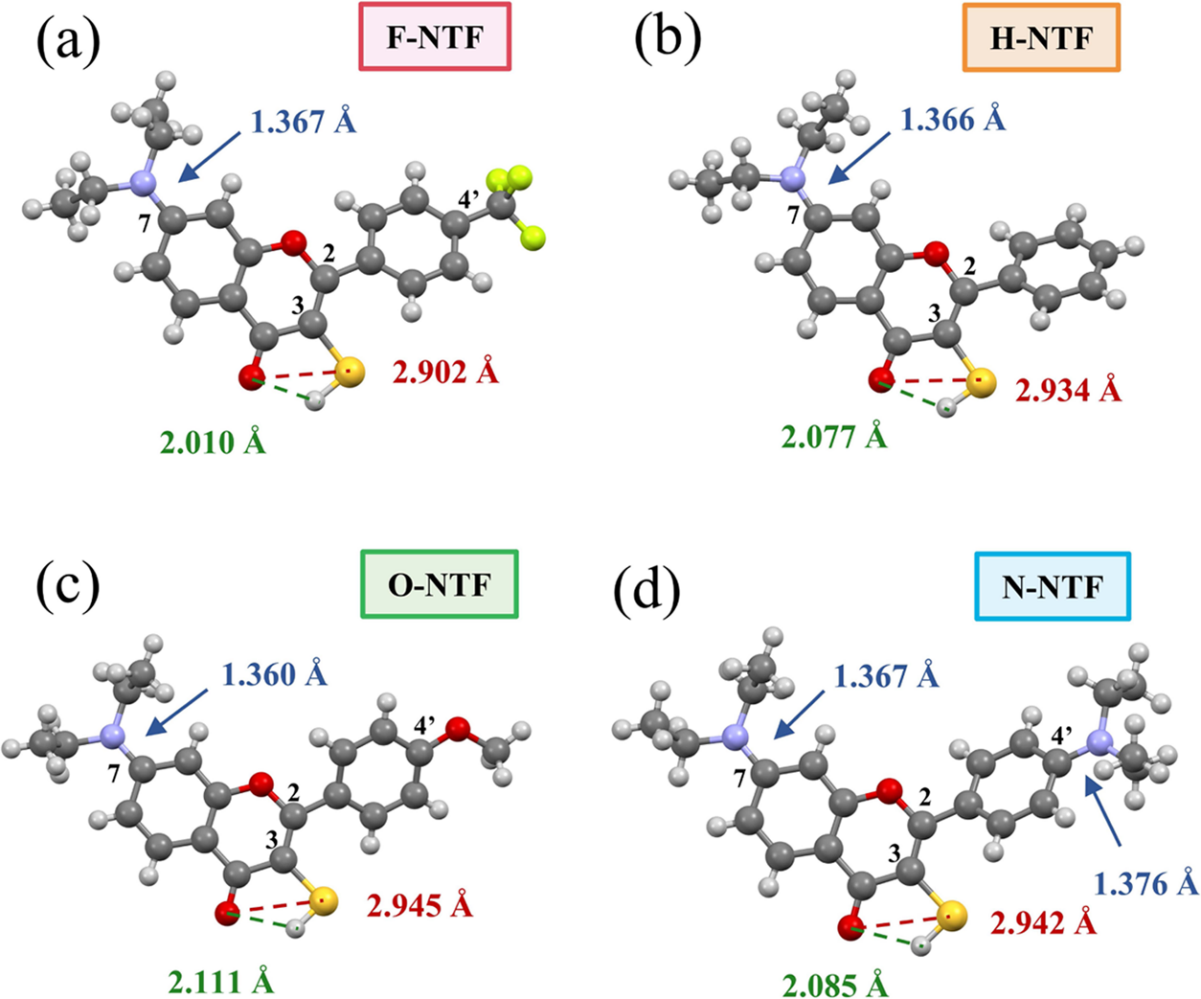
X-ray crystallographic structure of (a) **F-NTF**, (b) **H-NTF**, (c) **O-NTF**, and (d) **N-NTF** and the pertinent structure parameters. The displacement ellipsoids are drawn at the 50% probability level, and the H atoms are drawn as spheres of arbitrary radii.

Further support for H-bond formation is provided by single-crystal XRD and ^1^H NMR experiments. The single crystals of all synthesized compounds **H-NTF, N-NTF, O-NTF**, and **F-NTF** were successfully grown in ethyl acetate (EtOAc) either at −4 or 25 °C under an argon atmosphere and were analyzed by X-ray diffraction (XRD) techniques at 100 K. The ORTEP structures are shown in Figure 2. All of these crystal structures demonstrate comparable thiol–carbonyl distance (ranging from 2.902 to 2.945 Å, see Figure 2) to our previously reported 2.925 Å in **3NTF** (see Figure S47), suggesting the presence of intramolecular –SH---O=C hydrogen bonds. Moreover, the C(7)–N bonds of **NTFs** are all shorter (ranging from 1.360 to 1.367 Å, see Figure 2 and Table 1) than our previously reported C(4)–N bond in **3NTF** (1.371 Å, see Figure S47), revealing the superior donating ability of the C(7)-diethylamino group, verifying our π-elongation design. Similar dihedral angles between chromenone and the phenyl groups were observed in the C(4′)-substituted **F-NTF** (27.0°), **N-NTF** (35.0°), **O-NTF** (33.3°), and **3NTF** (28.2°; see Figure S47), substantiating the feasibility of synergistic manipulation of the hydrogen bond via C(7) and C(4′) substitution. To investigate the H-bond strength in **NTFs**, the S---O=C distances are determined to be 2.902 Å (**F-NTF**), 2.934 Å (**H-NTF**), 2.942 Å (**N-NTF**), and 2.945 Å (**O-NTF**; see Figure 2). Although the thiol hydrogen is intuitively added according to the computational optimization, where H atoms are drawn as spheres of arbitrary radii, the H-bond distance, defined by the distance between thiol hydrogen and carbonyl oxygen, is estimated to be in the order of 2.010 Å (**F-NTF**), 2.077 Å (**H-NTF**), 2.085 Å (**N-NTF**), and 2.111 Å (**O-NTF**) (see Figure 2). Among all four compounds, **F-NTF** presents the most deviated and the shortest in both S---O=C (2.902 Å) and –SH---O=C (2.010 Å) distance, clarifying that **F-NTF** possesses the strongest H-bond. **H-NTF** (2.934 Å) is determined to possess the second strongest hydrogen bond. Finally, the S---O=C distances of **N-NTF** and **O-NTF** are virtually identical (2.942 vs 2.945 Å), showing the indistinguishable intramolecular hydrogen bond strength of these two. Therefore, the intramolecular H-bond strength is inferred to be **F-NTF > H-NTF > N-NTF** ≈ **O-NTF** by single-crystal XRD.

**Table 1:** Hydrogen Bonding Parameters of NTFs Determined by XRD and NMR

struct\par	*d*_S-OC_ (Å)	*d*_SH–OC_ (Å)	*A*_NMR_ × 10^3^
**F-NTF**	2.902	2.010	–2.4
**H-NTF**	2.934	2.077	3.3
**O-NTF**	2.945	2.111	11
**N-NTF**	2.942	2.085	16

Also, the respective proton nuclear magnetic resonance spectra (^1^H NMR) of **F-NTF, H-NTF, O-NTF**, and **N-NTF** displayed sharp singlet peaks at δ 5.505, 5.385, 5.412, and 5.451 ppm in CDCl_3_, respectively (see Figures S41, S35, S39, and S37). To further evaluate the strength of these intramolecular H-bonds and the thiol acidity, the thiol chemical shifts of **F-NTF, H-NTF, O-NTF**, and **N-NTF** in DMSO-*d*_6_ were obtained to be 5.438, 5.361, 5.446, and 5.526 ppm, respectively (see Figure S48), where **F-NTF** showed the broadest peak followed by **H-NTF**, **O-NTF**, and **N-NTF**, demonstrating the large proclivity of sharing protons with the environment. Therefore, the acidity trend of thiol is deduced to be **F-NTF** > **H-NTF** > **O-NTF** > **N-NTF**, as determined by ^1^H NMR spectroscopy. The respective intramolecular H-bonding strengths are then quantified with Abraham’s method, as shown in eq 1
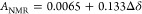
1where Δδ = δ (DMSO) – δ (CDCl_3_) denotes the difference between the chemical shifts between the two solvents.^76^

*A*_NMR_ values for **F-NTF, H-NTF, O-NTF**, and **N-NTF** are estimated to be −0.0024, 0.0033, 0.011, and 0.016, respectively. Taking the same trend as –OH- and –NH-type intramolecular H-bond efficiency, i.e., the lower the value, the higher the effectiveness,^76^ the trend of intramolecular H-bond strength is therefore deduced to be **F-NTF > H-NTF** > **O-NTF > N-NTF**, which is consistent with the results obtained from single-crystal XRD and IR. The above trend reveals that the –SH intramolecular H-bonding strength correlates with –SH acidity, being increased upon the increase of –SH acidity.

### Steady-State Photophysical Properties

**Figure 3:**
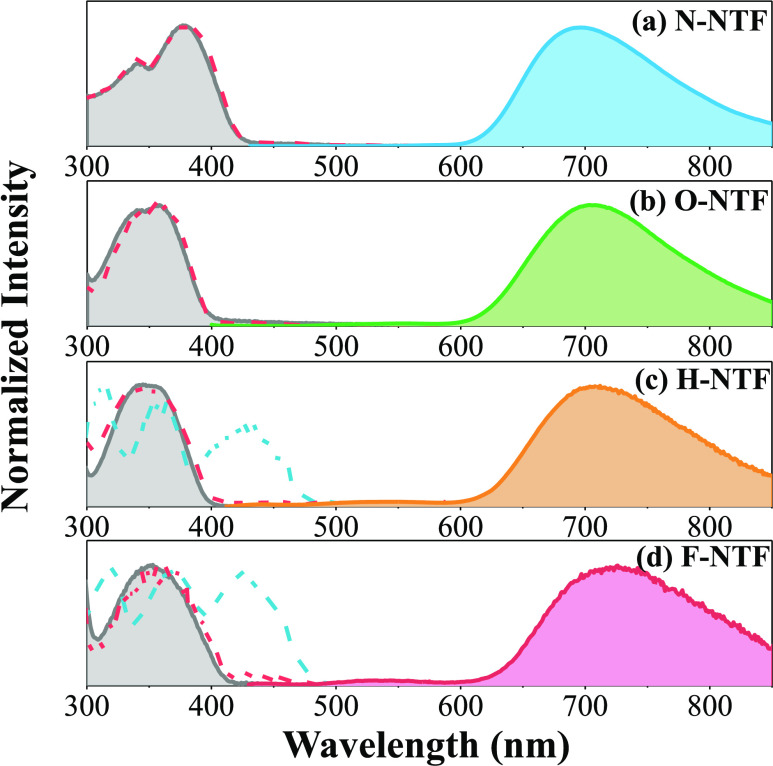
Steady-state absorption and emission properties of (a) **N-NTF**, (b) **O-NTF**, (c) **H-NTF**, and (d) **F-NTF** in cyclohexane. The absorption spectra are all depicted with shaded, gray solid lines in the respective row. The emission spectra upon 390 nm excitation of **N-NTF**, **O-NTF**, **H-NTF**, and **F-NTF** are depicted in blue, green, orange, and red solid lines, respectively. The excitation spectra monitoring at 750 and 530 nm are depicted in the left-sided red and blue dashed lines, respectively.

As shown in Figure 3a, we start from **N-NTF** in cyclohexane, where **N-NTF** reveals a major absorption band that is maximized at 380 nm. Upon excitation at the absorption peak wavelength, the emission is dominated by a 695 nm emission band. By monitoring at the 695 nm emission band, the corresponding excitation spectrum maximized at 380 nm is identical to the absorption spectrum (Figure 3a). Therefore, the 695 nm emission with a Stokes shift as large as ∼12,000 cm^–1^ (energy difference between absorption and emission peak frequencies) is reasonably assigned to the proton-transfer tautomer emission via the –SH-associated ESIPT, as previously concluded for **3NTF** (see Figure 1).^67^ For further support, a compound thiol of **N-NTF** being methylated, i.e., **Me-N-NTF**, was also synthesized (see Figures S44 and S45). Upon 360 nm excitation of dilute **Me-N-NTF** in cyclohexane, except for the normal emission maximized at 480 nm, no emission with a large Stokes shift in the red is observed (see Figure S49). This result supports the notion that the emission at 695 nm observed in **N-NTF** originates from the ESIPT of the thiol proton.

Similar patterns were observed for **O-NTF** in the steady-state absorption and their corresponding solely large red-shifted 700 nm emission that is assigned to the proton-transfer emission (see Figure 3b). For **H-NTF** and **F-NTF**, note that in addition to the major proton-transfer emission in the red, a trace but non-negligible emission band around 500–570 nm was observed and denoted as the F_short_ band (see Figure 3c,d). Notably, the measurements were conducted under a low concentration of 10^–4^–10^–5^ M; hence, the possibility of origin from the aggregation effect has been ruled out. The excitation spectrum monitored at the F_short_ band, for example, 530 nm, reveals a band at 420–470 nm that is entirely different from that of the absorption spectrum. In other words, the excitation spectrum displays a negligible absorbance in the absorption spectrum (Figure 3c,d). The results suggest that the F_short_ emission must emanate from trace amounts of interfering species that possess a significantly higher emission quantum yield (QY) than the predominant proton-transfer tautomer emission having low QY (see Table 2). As elaborated in the SI for details, because the F_short_ intensity gradually decreased after a series of different stages of purification, especially a drastic reduction of F_short_ intensity after vacuum sublimation, the results eliminate previously proposed ion pair formation.^67^ Instead, it is more plausible to infer the existence of trace impurities in **H-NTF** and **F-NTF** even after thorough purification in this study. As for the possible structure of impurity, experiments depicted in the SI have eliminated the S–S-linked dimer of 3-mercaptoflavones and suggested the origin of the photooxygenation product.^77^ Unfortunately, trace product yields are forbidden for further characterization. Nevertheless, the current compound purity after sublimation meets spectroscopic demand. This viewpoint can be firmly supported by the corresponding spectra of the studied **NTFs** in solid powder where ESIPT takes place with good QY of the proton-transfer emission (>5 × 10 ^–3^). As a result, all **NTFs** exhibit dominant proton-transfer tautomer emission in the range of 690–720 nm (see Figures S55 and S56), free from impurity interference.

**Table 2:** Photophysical and Population Lifetime Data of NTFs^a^^b^

sample	λ_tau_ (nm)	PLQY_tau_ (%)	τ_short_ (ns)	τ_tau_ (ps)	*k*_r(tau)_ (s^–1^)	*k*_nr(tau)_ (s^–1^)
**N-NTF**	695 (667^c^)	1.66 (6.82^c^)	-	358 (1470^c^)	4.64 × 10^7^	1.84 × 10^9^
**O-NTF**	700 (669^c^)	0.68 (2.45^c^)	-	257 (925^c^)	2.65 × 10^7^	3.87 × 10^9^
**H-NTF**	705 (671^c^)	0.18 (0.58^c^)	2.4	127 (488^c^)	1.18 × 10^7^	7.87 × 10^9^
**F-NTF**	720 (702^c^)	0.15 (0.70^c^)	3.1	120 (468^c^)	1.50 × 10^7^	8.33 × 10^9^

a Note: τshort for F_short_ emission is acquired by excitation at 450 nm, monitoring at 530 nm. τ_tau_ for F_tau_ emission is acquired by excitation at 390 nm, monitoring at 750 nm. The tautomer decay curves of solid powder are shown in Figure S56.

b Data were obtained in cyclohexane.

c Data were obtained from solid powders.

### Time-Resolved Photophysical Properties

**Figure 4:**
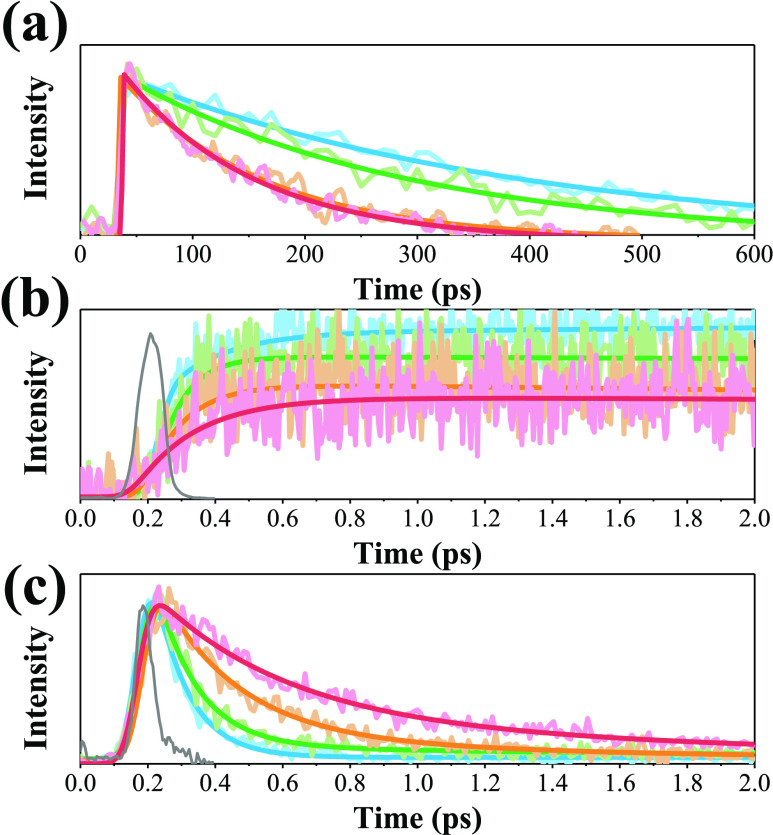
Population decay of **NTFs** in toluene: The kinetic trace at (a) 750 nm (tautomer decay), (b) 700 nm (tautomer rise), and (c) 470 nm (normal decay) emission. **N-NTF** (blue line), **O-NTF** (green line), **H-NTF** (orange line), and **F-NTF** (red line) and the instrument response function (IRF, gray line) acquired by the fluorescence upconversion method, λ_ex_ = 400 nm.

Time-resolved measurements were conducted with either time-correlated single photon counting (TCSPC) or femtosecond fluorescence upconversion technique (experimental details are given in Section 4 in the SI). We first performed TCSPC in a picosecond time range to investigate the fluorescence population lifetime of the titled compounds. As a result, the lifetime of proton-transfer tautomer emission, monitored at 750 nm, was resolved to be several hundred picoseconds for the studied **NTFs** (see Figure 4a and Table 2). Assuming that ultrafast ESIPT (vide infra) leads to 100% ESIPT efficiency, the emission quantum yield (QY) was measured to be 1.66, 0.68, 0.18, and 0.15% for **N-NTF**, **O-NTF**, **H-NTF**, and **F-NTF**, respectively. Accordingly, the radiative rate constant *k*_r_, deduced by *k*_r_ = QY × τ_obs_^–1^, is determined to be 4.64 × 10^7^, 2.65 × 10^7^, 1.18 × 10^7^, and 1.50 × 10^7^ s^–1^, respectively. All pertinent data are given in Table 2. Similar *k*_r_ but a large difference in QY indicates the dominant nonradiative decay rate *k*_nr_ (Table 2) plausibly governed by the energy gap law.^78−81^ That is, *k*_nr_ increases as the emission energy gap decreases from **N-NTF** (695 nm) to **F-NTF** (720 nm) in the near IR region. In comparison, upon monitoring at the F_short_ band such as 530 nm, the population lifetime was measured to be several nanoseconds (Table 2). Because the responsible F_short_ species has negligible absorbance, *k*_r_ and QY could not be deduced in the current stage. However, assuming regular ππ* configuration with an allowed transition at ∼450 nm, *k*_r_ is theoretically around 10^8^–10^9^ s^–1^. Therefore, the QY of the F_short_ emission is expected to be ≫10%, which should be much higher than that of the tautomer emission (Table 2), consistent with our early proposal of trace amount giving F_short_ emission (vide supra).

To gain further insights into the ESIPT, we probe the early dynamics in the femtosecond range of the corresponding emission by the fluorescence upconversion measurement. Our current Ti:sapphire femtosecond laser has been upgraded to 35 fs for the fundamental laser output at 800 nm. This, coupled with the use of off-axis parabolic mirrors and the colinear pump–probe configuration into a 0.1 mm BBO crystal, gives a system response time of 75 fs after convolution for the fluorescence upconversion experiment (for more details, see Section 4.2 in the SI). Note that due to the low solubility of **NTFs** in cyclohexane, the upconversion experiment was carried out in toluene. As a result, upon monitoring the proton-transfer tautomer emission at, e.g., 700 nm, the early rise dynamics of ESIPT are resolvable for all **NTFs** and are shown in Figure 4b. For example, the **N-NTF** tautomer emission shows a fast rise time of 122 fs, followed by a nearly constant intensity in the acquisition range of 2 ps, which should be ascribed to the population decay of 358 ps measured in the TCSPC experiment. Accordingly, as listed in Table 3, the rise time of the proton-transfer tautomer emission is in the order of **N-NTF** (122 fs) < **O-NTF** (149 fs) < **H-NTF** (285 fs) < **F-NTF** (428 fs). We also made efforts to probe the decay dynamics of the normal emission, which is expected to be around 430–480 nm and whose intensity is near zero due to the ultrafast ESIPT. As a result, the kinetic trace monitored at 470 nm for all **NTFs** is also depicted in Figure 4c and the early fast decay time (= *k*_PT_^–1^, where *k*_PT_ denotes the rate constant of ESIPT, assuming its domination on the relaxation) is fitted to be in the order of **N-NTF** (101 fs) **< O-NTF** (123 fs) < **H-NTF** (232 fs) < **F-NTF** (398 fs), which, within the experimental uncertainty, correlates well with the rise component of the tautomer emission. The result clearly shows a precursor–successor type of kinetic relationship for ESIPT. To ensure reproducibility, the above upconversion experiments were repeated at least three times, and the % of uncertainty is no more than 10% for the fast decay component.

**Table 3:** Early Dynamics of ESIPT in Terms of Fast Decay and Rise Components at 470 and 700 nm, Respectively, in Toluene

sample	decay of normal form (fs)	rise of tautomer form (fs)
**N-NTF**	101	122
**O-NTF**	123	149
**H-NTF**	232	285
**F-NTF**	398	428

The results of the ESIPT dynamics attract our attention. As shown in Table 3, the trend of *k*_PT_ for ESIPT in the order of **N-NTF** > **O-NTF** > **H-NTF** > **F-NTF** lacks correlation with the H-bonding strength obtained experimentally (^1^H NMR, IR, and X-ray, see Table 1). For example, the rate of ESIPT was measured to be ∼(398 fs)^−1^ for **F-NTF**, which is the slowest among the studied **NTFs**, while its H-bonding strength proves to be the strongest (vide supra). On the other hand, the rate of ESIPT for **N-NTF** was measured to be the fastest one (101 fs)^−1^, whereas its H-bond strength ranks fourth among the four studied compounds. Therefore, the result for **NTFs** seems to lack a relationship between the H-bonding strength and ESIPT dynamics. In addition, as shown in Figure S54, the acidity of the thiol hydrogen was also assessed through the p*K*_a_ value, which was determined by pH absorption titration in combination with computational methods. The results also indicate that the rate of ESIPT does not exhibit a relationship with the acidity of the thiol.

The above results of ESIPT dynamics for **NTFs** are distinct from the –OH- and –NH-type ESIPT, where the correlation among structure, H-bonding properties, and dynamics/thermodynamics of ESIPT has been widely studied and established.^35,36,68,69^ Empirically, for the same class of the ESIPT molecules, i.e., the same π-conjugated core chromophore modified by different substituents to fine-tune the H-bonding strength, the correlation for the H-bonding strength versus the –OH (–NH) ESIPT dynamics follows a trend. That is, the increase in the H-bonding strength leads to an increase in the ESIPT rate for the same class of ESIPT molecules.

Elaborated in the early section, the –OH (–NH)-associated H-bond lies in its share of the proton between two atoms, both of which possess large electron negativity. Differently, the –SH-associated H-bond relies on the large size and, hence, the large polarization of the sulfur atom. Considering the lack of relationship between H-bonding strength and ESIPT dynamics for **NTFs**, its ESIPT mechanism may be distinct intrinsically from that of the classical –OH (–NH)-type ESIPT. This leads us to explore an alternative pathway participated by the proton-accepting site, i.e., the basicity site at the carbonyl oxygen, for thiol-ESIPT. In the same class of **NTFs**, the substituent effect at C(4′) alters the property of the C=O bond, where an increase in the C=O bond distance implies a more single-bond-like property. Consequently, the increased sp^3^ character leads to enhanced nucleophilicity, resulting in stronger basicity of the carbonyl oxygen. This viewpoint can be proven by the CO bond length obtained by single-crystal XRD: with **N-NTF**, equipped with the strongest electron-donating group on C(4′) among all four **N-NTFs**, renders the longest C=O bond (1.244 Å, i.e., the most basic C=O) and therefore the largest *k*_PT_; on the contrary, **F-NTF**, with the strongest electron-withdrawing group on C(4′), yields the shortest C=O bond (1.237 Å, i.e., the least basic C=O) and consequently the smallest *k*_PT_.

Because all **NTFs** decomposed under strongly acidic conditions, it was infeasible to measure the basicity of C=O by pH titration. Nevertheless, we expected that the carbonyl oxygen of **N-NTF** and **O-NTF** would be the top two most basic proton acceptors due to the π-conjugation effect, hence, the corresponding photoinduced charge transfer in the excited state. The order of carbonyl oxygen basicity is anticipated to be **N-NTF** > **O-NTF** > **H-NTF** > **F-NTF** in both ground and excited states. This viewpoint can be verified with the assistance of a computational approach. In this study, the computational approach utilized the B3LYP/6-311++g(3df,3pd)^82−84^ level via the Gaussian 16 package^85^ to estimate the more accurate energies. Vibrational modes were also analyzed to confirm the minimum energy for the optimized state. Solvation corrections for the solvent used (toluene, ε = 2.38)^86^ were included using the polarizable continuum model (PCM).^87^

**Figure 5:**
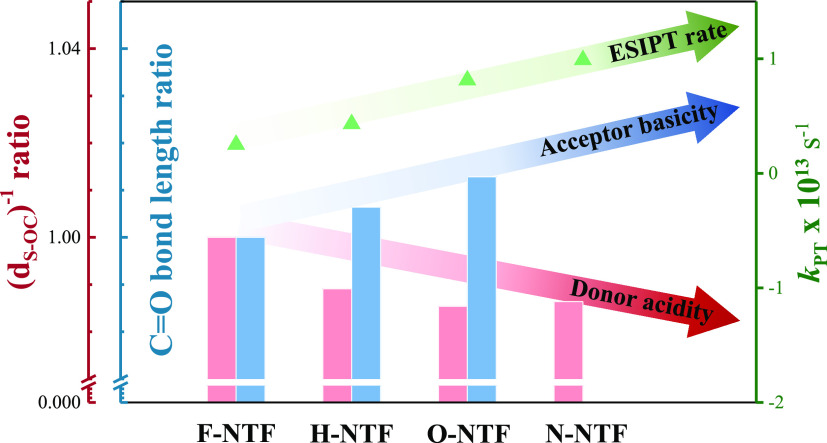
Correlation between the ESIPT rate and several H-bond-related properties of **NTFs**. The ESIPT rate is denoted with *k*_PT_ (green triangle and arrow). The properties include the H-bond strength (red column), the related donor acidity (red arrow), and the excited-state acceptor basicity (blue column and arrow).

**Figure 6:**
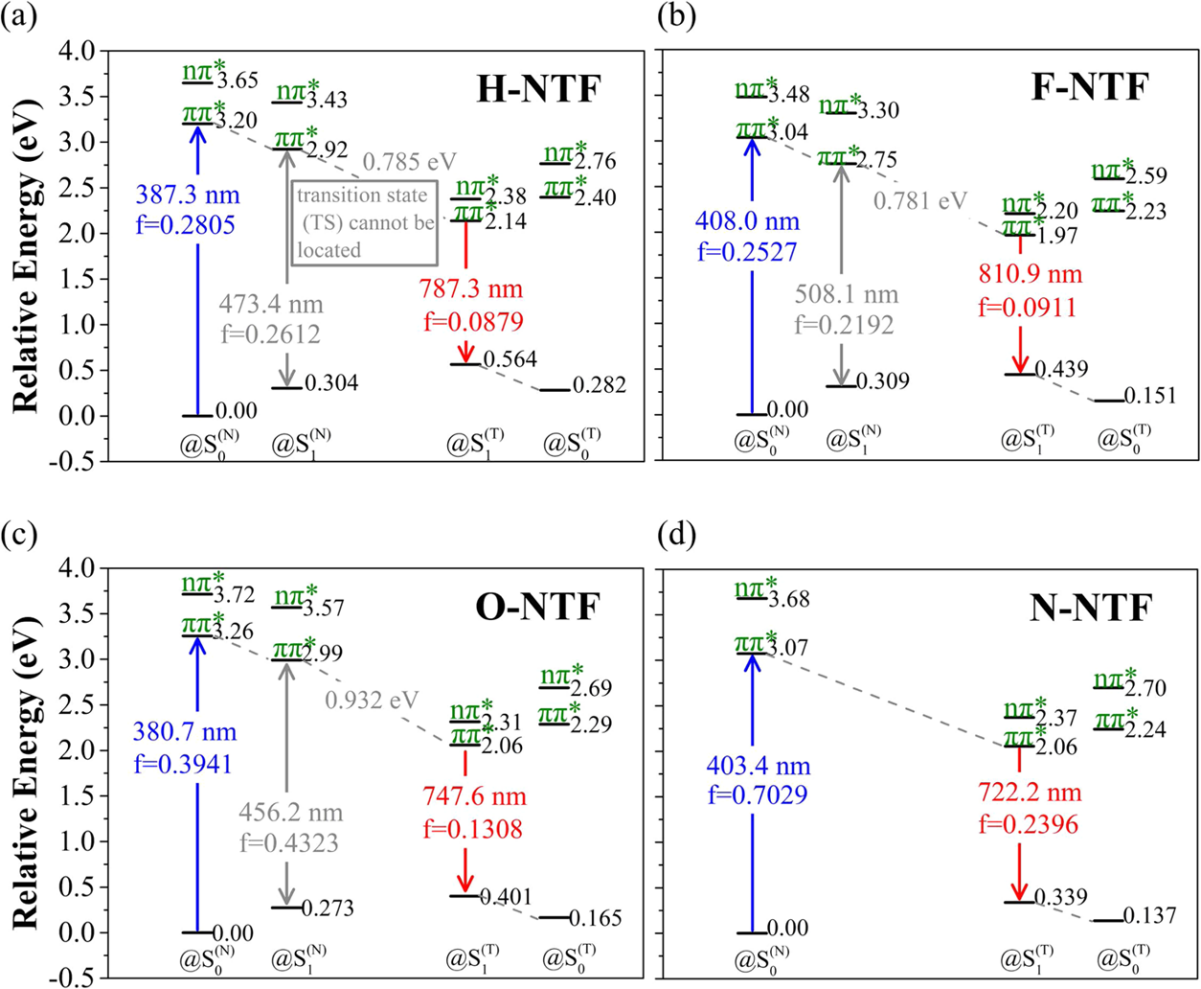
Calculated energy of the lower lying states and their corresponding electronic character for (a) **H-NTF**, (b) **F-NTF**, (c) **O-NTF**, and (d) **N-NTF**, where @S_0_^(N)^, @S_1_^(N)^, @S_0_^(T)^, and @S_1_^(T)^ denote the geometry optimized states at S_0_^(N)^, S_1_^(N)^, S_0_^(T)^, and S_1_^(T)^, respectively. For **N-NTF**, note that the geometry optimization at the Franck–Condon excited S_1_ (N) state relaxes spontaneously to the S_1_ (T) state. Additionally, “transition state (TS) cannot be located” is depicted in a square block using compound **H-NTF** and likewise in (b–d). For the transition state (TS) of all title compounds, the S1 geometry of the Franck–Condon transition state and hence barrier cannot be calculated. The results indicate that the potential energy surface along the ESIPT coordinate is nearly barrierless, consistent with the large *k*_PT_ of ultrafast ESIPT among all studied **NTFs**.

As a result, the calculated bond lengths of C=O at S_0_- and S_1_-optimized structures of the normal form for all **NTFs** are listed in Tables 4 and S6. Table 4 also lists the C=O bond distance determined by single-crystal XRD, where the data correlate well with the calculated value in S_0_, validating the computational approach. Furthermore, the difference in the C=O distance between S_0_ and S_1_, denoted Δ(C=O) (=S_1_(C=O)–S_0_) (C=O), (Table 4) in theory should refer to the changes in basicity. In other words, the larger the Δ(C=O), the greater the change in the basicity from S_0_ to S_1_. Note that in Table 4, S_1_(C=O) for **N-NTF** normal species is not accessible due to its spontaneous relaxation to the proton-transfer tautomer state S_1_^(T)^ (see Figure 6). The results indicate that Δ(C=O) for **N-NTF**, if it exists, should be the largest among all **NTFs**. Accordingly, the increase of the Δ(C=O) value, shown in Table 4, is in the order of **F-NTF** < **H-NTF** < **O-NTF** < **N-NTF**, consistent with the same trend as *k*_PT_ of ESIPT. That is, the larger changes in C=O basicity led to a faster rate of ESIPT (Figure 5) for **NTFs**.

**Table 4:** Calculated Bond Lengths of C=O at S_0_- and S_1_-Optimized Structures of Normal Form and Experimental Results of C=O Bond Length Determined by Single-Crystal XRD

bond\structure	**F-NTF**	**H-NTF**	**O-NTF**	**N-NTF**
C=O @S_0_-opt (Å)	1.233	1.234	1.234	1.236
C=O @S_1_-opt (Å)	1.248	1.256	1.264	-
Δ (C=O)^a^ (Å)	0.015	0.022	0.030	-
C=O @Exp (Å)	1.237	1.240	1.238	1.244

a Derived by deducing C=O @S_0_-opt from C=O @S_1_-opt.

We also exploited relevant data to assess the ESIPT thermodynamics. Note that we focus only on the thermodynamics of the ESIPT reaction from the electronically excited **NTFs** to their proton-transfer tautomer species and their associated state configurations. The energy of each state and its corresponding electronic character are shown in Figure 6, while the associated frontier orbital configurations and the pertinent data are depicted in Figure S57 and Tables S5–S7. Several remarks can be summarized according to the computational results: (i). The most stable form in the singlet excited states is ascribed to the tautomer S_1_^(T)^ state (T denotes the tautomer), indicating thermodynamically favorable ESIPT for all **NTFs**. (ii). For all **NTFs**, both S_1_^(N)^ and S_1_^(T)^ states are in a ππ* configuration, consistent with our original design of adding –N(C_2_H_5_)_2_ at the C(7) position to elongate the π-conjugation. (iii) The calculated S_1_^(T)^ →S_0_^(T)^ Franck–Condon transition at 722 nm (**N-NTF**), 747 nm (**O-NTF**), 787 nm (**H-NTF**), and 810 nm (**F-NTF**) has the same trend as the experimental values in cyclohexane (see Figure 3), except that the calculated values are in the lower energy due perhaps to the functional being chosen and use of an incomplete basis set. (iv) For **N-NTF**, the geometry optimization at the Franck–Condon excited S_1_^(N)^ state relaxes spontaneously to S_1_^(T)^, indicating its rather small barrier in ESIPT, rationalizing its fastest ESIPT of ∼(101 fs)^−1^ among all studied **NTFs**. (v) The energy difference between S_1_^(N)^ and S_1_^(T)^ (Δ*E*_ESIPT_ = S_1_^(T)^–S_1_^(N)^) is −0.781 eV (**F-NTF**) > −0.785 eV (**H-NTF**) > −0.932 eV (**O-NTF**) > −1.01 eV (**N-NTF**, taking S_1_^(N)^ geometry @S_0_^(N)^, see Figure 6). Compared to the ESIPT rates shown in Figure 5, we found a good correlation in that the more exothermic Δ*E*_ESIPT_ leads to faster ESIPT. As for (v), recently, the semiempirical Bell–Evans–Polanyi (BEP) principle has been applied to –OH– and –NH-type ESIPT, showing that under the same class of ESIPT analogues, the more exothermic Δ*E*_ESIPT_ leads to the smaller barrier and hence faster ESIPT.^35,36,69^ Although the non-Pauling-type thiol H-bonded **NTFs** show unconventional ESIPT dynamics where its rate does not correlate with the H-bonding strength, the BEP principle holds for **NTFs**. This may not be too surprising because the BEP principle considers the general chemical reaction and not specifically for the ESIPT reaction.

## Conclusions

In summary, we have strategically designed and synthesized a new class of thiol intramolecular H-bonded molecules (**NTFs**) and investigated their properties in both the ground and excited states. All studied **NTFs** undergo ESIPT, giving a >690 nm proton-transfer tautomer emission in nonpolar solvents. Comprehensive steady-state and time-resolved UV–vis–NIR spectroscopies conclude that the thiol-ESIPT rate does not correlate with the H-bonding strength that is commonly observed for the –OH (–NH)-type ESIPT, i.e., the ESIPT rate increases with the H-bond strength. Instead, the rate of ESIPT increases upon the increase in the basicity of the proton acceptor. Fundamentally, the result points to the intrinsic difference between nonclassical –SH- and Pauling-type –OH H-bonds, where the proton-donating site is governed by the diffusive electron density and electron negativity, respectively. For the latter, the H-bonding strength or the acidity of the proton-donating site determines the –OH-type ESIPT dynamics and thermodynamics. For the former, the H-bonding strength seems to play a minor role, while the proton-accepting site is a key for ESIPT, at least for **NTFs**. As for generalization, more –SH classes of ESIPT need to be explored, which should advance our fundamental knowledge of the non-Pauling-type H-bond and its associated proton-transfer reaction.

## Supporting Information Available

Details of synthetic procedures, additional NMR spectra, and crystal structures of **N-NTF**, **O-NTF**, **H-NTF**, and **F-NTF**; computational approach; and supporting photophysical measurements (PDF)
