# Supplementary material for: Hydrogen-Bonded Thiol Undergoes Unconventional Excited-State Intramolecular Proton-Transfer Reactions
Source: J Am Chem Soc. 2024 Jan 30;146(5):3125–35. doi: 10.1021/jacs.3c10405 (PMC10859960; doi:10.1021/jacs.3c10405)
Supplement: Supplementary file 1 — ja3c10405_si_001.pdf [file ja3c10405_si_001.pdf]

Supporting Information

Hydrogen-Bonded Thiol Undergoes Unconventional Excited-State Intramolecular Proton Transfer Reactions

Jian-Kai Wang,¶ Chih-Hsing Wang,¶ Chi-Chi Wu,¶ Kai-Hsin Chang, Chun-Hsiang Wang, Yi-Hung Liu, Chao-Tsen Chen,* Pi-Tai Chou*

Department of Chemistry, National Taiwan University, Taipei, 10617, Taiwan, R.O.C.

Center for Emerging Material and Advanced Devices, National Taiwan University, Taipei, 10617, Taiwan, R.O.C.

¶These three authors contributed equally

Corresponding Authors:

* Chao-Tsen Chen: chenct@ntu.edu.tw

* Pi-Tai Chou: chop@ntu.edu.tw

Table of Contents

1. **General Experimental Section** **S-4**
2. **Experimental Procedures** **S-5**

Figure S1. ^{1}H NMR spectrum in $CDCl_3$ acquired from the crude of route 1 in **scheme 1** indicating the presence of both **H-NTF** (highlighted in red color), and **H-NSSF** (highlighted in blue color) and the ratio of **H-NTF** and **H-NSSF** is 2:1. S-18

3. **Structure Characterization** **S-19**

Figure S2. ^{1}H NMR of **2b** in $CDCl_3$ S-19
Figure S3. ^{13}C NMR of **2b** in $CDCl_3$ S-19
Figure S4. ^{1}H NMR of **2c** in $CDCl_3$ S-20
Figure S5. ^{13}C NMR of **2c** in $CDCl_3$ S-20
Figure S6. ^{1}H NMR of **2d** in $CDCl_3$ S-21
Figure S7. ^{13}C NMR of **2d** in $CDCl_3$ S-21
Figure S8. ^{19}F NMR of **2d** in $CDCl_3$ S-22
Figure S9. ^{1}H NMR of **s1** in $CDCl_3$ S-23
Figure S10. ^{13}C NMR of **s1** in $CDCl_3$ S-23
Figure S11. ^{1}H NMR of **s2** in $CDCl_3$ S-24
Figure S12. ^{13}C NMR of **s2** in $CDCl_3$ S-24
Figure S13. ^{1}H NMR of **5** in $CDCl_3$ S-25
Figure S14. ^{13}C NMR of **5** in $CDCl_3$ S-25
Figure S15. ^{1}H NMR of **6** in $CDCl_3$ S-26
Figure S16. ^{13}C NMR of **6** in $CDCl_3$ S-26
Figure S17. ^{1}H NMR of **3a** in $CDCl_3$ S-27
Figure S18. ^{13}C NMR of **3a** in $CDCl_3$ S-27
Figure S19. ^{1}H NMR of **3b** in $CDCl_3$ S-28
Figure S20. ^{13}C NMR of **3b** in $CDCl_3$ S-28
Figure S21. ^{1}H NMR of **3c** in $CDCl_3$ S-29
Figure S22. ^{13}C NMR of **3c** in $CDCl_3$ S-29
Figure S23. ^{1}H NMR of **3d** in $CDCl_3$ S-30
Figure S24. ^{13}C NMR of **3d** in $CDCl_3$ S-30
Figure S25. ^{19}F NMR of **3d** in $CDCl_3$ S-31
Figure S26. ^{1}H NMR of **H-NSSF** in $CDCl_3$ S-32
Figure S27. ^{13}C NMR of **H-NSSF** in $CDCl_3$ S-32
Figure S28. ^{1}H NMR of **N-NSSF** in $CDCl_3$ S-33
Figure S29. ^{13}C NMR of **N-NSSF** in $CDCl_3$ S-33
Figure S30. ^{1}H NMR of **O-NSSF** in $CDCl_3$ S-34

Figure S31. ^{13}C NMR of **O-NSSF** in $CDCl_3$ S-34
Figure S32. ^{1}H NMR of **F-NSSF** in $CDCl_3$ S-35
Figure S33. ^{13}C NMR of **F-NSSF** in $CDCl_3$ S-35
Figure S34. ^{19}F NMR of **F-NSSF** in $CDCl_3$....S-36
Figure S35. ^{1}H NMR of **H-NTF** in $CDCl_3$ S-37
Figure S36. ^{13}C NMR of **H-NTF** in $CDCl_3$ S-37
Figure S37. ^{1}H NMR of **N-NTF** in $CDCl_3$....S-38
Figure S38. ^{13}C NMR of **N-NTF** in $CDCl_3$ S-38
Figure S39. ^{1}H NMR of **O-NTF** in $CDCl_3$ S-39
Figure S40. ^{13}C NMR of **O-NTF** in $CDCl_3$ S-39
Figure S41. ^{1}H NMR of **F-NTF** in $CDCl_3$....S-40
Figure S42. ^{13}C NMR of **F-NTF** in $CDCl_3$....S-40
Figure S43. ^{19}F NMR of **F-NTF** in $CDCl_3$ S-41
Figure S44. ^{1}H NMR of **Me-N-NTF** in $CDCl_3$....S-42
Figure S45. ^{13}C NMR of **Me-N-NTF** in $CDCl_3$....S-42
Figure S46. S-H stretching wavenumber obtained via FTIR (KBr). The SH stretching peak wavenumber of **F-NTF**, **H-NTF**, **O-NTF**, and **N-NTF** are depicted in red, orange, green, and blue respectively....S-43
Table S1. Single-crystal XRD data for **F-NTF** (CCDC 2295378)....S-44
Table S2. Single-crystal XRD data for **H-NTF** (CCDC 2295381)....S-45
Table S3. Single-crystal XRD data for **O-NTF** (CCDC 2295379)....S-46
Table S4. Single-crystal XRD data for **N-NTF** (CCDC 2295382)....S-47
Figure S47. Single-crystal XRD structure of **F-NTF**, **H-NTF**, **O-NTF**, **N-NTF**, and **3NTF**.[1] The displacement ellipsoids are drawn at the 50% probability level, and the H atoms are drawn as spheres of arbitrary radii....S-48
Figure S48. S-H chemical shifts in DMSO-d_6 for **F-NTF**, **H-NTF**, **O-NTF**, and **N-NTF.** S-48
4. **Photophysical Properties S-49**
4.1 Steady-State Spectra, PLQY and Lifetime Measurements....S-49
Figure S49. The steady-state absorption, emission, and excitation spectra of Me-N-NTF in cyclohexane. The excitation wavelength for the emission is 360 nm. S-49
4.2 Time-resolved fluorescence spectroscopy....S-49
Figure S50. The decay curves were obtained from repeated scans of **F-NTF** in toluene. This result suggests that photodegradation during the fs-upconversion measurement period is negligible. (excitation wavelength: 400 nm)....S-50
4.3 Impurity issue S-51
Figure S51. The absorption spectrum of **N-NSSF** (i.e., **N-NTF** dimer with an

S-S linkage) in cyclohexane. S-52
Figure S52. The absorption (a) and emission (b) spectrum of **F-NTF** in cyclohexane. The emission spectrum was excited at 360 nm. The F-NTF solution was exposed by 360 nm UV light (~70 μW) and measured with an interval of every 20 mins. Furthermore, the crude of this reaction was also analyzed with LC-ESI-MS and the trace and mass spectrum of photoproduct are shown in (c) and (d) respectively. S-53
Figure S53. The compared steady-state spectrum of **NTFs** before (left) and after (right) additional sublimation purified. S-54
Figure S54. The pKa value of each compound is achieved by the experiment and calculation respectively...S-55
Figure S55. The emission and absorption spectra of the studied **NTF**s in solid powder. The absorption spectra of solid powders were measured by an integrating sphere. The emission spectra were measured by 390 nm excitation. Note that all **NTFs** exhibit solely the proton-transfer tautomer emission........S-56
Figure S56. The tautomer emission lifetime of solid powder **NTFs** was measured using the TCSPC technique (see section 4), with a 377.8 nm pulse laser used as the excitation wavelength. The emission was monitored at 750 nm. S-56
5. Computational Section S-57
Table S5. The computed optical excitations and molecular orbital contributions for all title compounds...S-57
Figure S57. Frontier molecular orbitals associated with major optical transitions of (a) normal form at S_0-optimized structure and (b) tautomer form at S_1-optimized structure for all title compounds. S-58
Table S6. The distances (Å) between S-H, H…O and C=O in S_0 and S_1-optimized geometries of normal forms...S-58
Table S7. The cartesian coordinates of the normal forms and tautomers of S_0 and S_1-optimized **F-NTF**, **H-NTF**, **O-NTF** and **N-NTF**......................................S-59
6. References..S-71

1. General Experimental Section

All chemicals were purchased from commercial sources and used as received. Solvents used for syntheses were dried either by standard literature methods before being distilled or by the drying solvent system (SPBT-1 bench top solvent purification system, LC Technology Solutions, Inc.) and stored under nitrogen over 4Å molecular sieves after drying. Merck silica gel 60 F254 was used as TLC plate, visualized by UV light or staining reagents such as PMA. Flash column chromatography was performed on Silica gel 60 (230-400 mesh ASTM). ^{1}H (400 MHz), ^{13}C (100 MHz), and ^{19}F (376 MHz) spectra were recorded on a Bruker AVIII HD 400 NMR or a Bruker AVIII 400 NMR. Chemical shifts are reported relative to $CDCl_3$ solvent peaks (7.26 ppm for ^{1}H from residual $CHCl_3$ and 77.16 ppm for ^{13}C) and quoted as δ values in ppm. The following abbreviations are used: singlet (s), doublet (d), triplet (t), and multiplet (m). Melting points were determined on a Fargo MP-1D melting point apparatus without further correction. Infrared spectra were recorded on a Bruker Vertex 70 FTIR or a Nicolet iS50 FTIR spectrometer. Mass spectra with an electrospray ionization (ESI) were collected on a Bruker microTOF-Q Ⅱ. Single crystal structures were determined by a Bruker AXS D8 Venture diffractometer with an Oxford Cryostream 800+.

2. Experimental Procedures

A general procedure for the synthesis of **2b**, **2c**, and **2d**

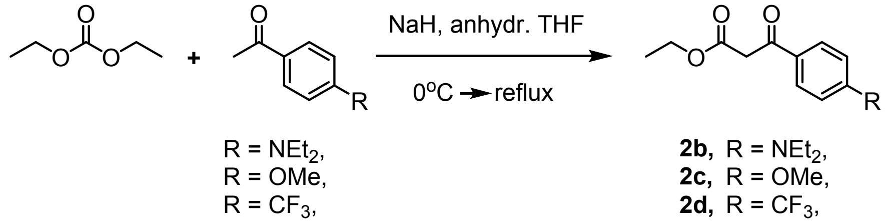

To an anhydrous THF (75 mL) solution of diethyl carbonate (9.52 mL, 78 mmol, 2.5 equiv.) and NaH (60% dispersion in mineral oil, 6.27 g, 157 mmol, 5 equiv.), a solution of 4-diethylaminoacetophenone or 4-methoxyacetophenone, or 4-trifluoromethylacetopenone (31 mmol, 1.0 equiv.) in anhydrous THF (75 mL) was added via an addition funnel at 0 °C over 10 min. The resulting solution was refluxed with stirring and monitored by TLC. After all the starting materials were consumed, the reaction was cooled to 0 °C and quenched with saturated aqueous NH_4Cl solution (50 mL), followed by extraction with EtOAc (100 mL*3) and water (50 mL*1). The combined organic layers were wash with brine (10 mL), dried over $MgSO_4$, and concentrated. The crude product was purified by flash column chromatography to afford 1-ethoxy-3-(4'-substituted phenyl)propane-1,3-diones (**2b-d**).

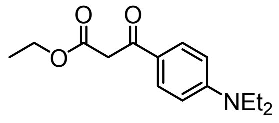

2b

1-ethoxy-3-(4'-diethlaminophenyl)propane-1,3-dione (2b)

90% yield. Light brown viscous liquid. R_f = 0.32 (EtOAc/Hex = 1/4). ^{1}H NMR (400 MHz, $CDCl_3$) δ 7.77 (d, J = 9.2 Hz, 2H), 6.56 (d, J = 9.2 Hz, 2H), 4.14 (q, J = 7.1 Hz, 2H), 3.83 (s, 2H), 3.36 (q, J = 7.1 Hz, 4H), 1.20 (t, J = 7.1 Hz, 3H), 1.13 (t, J = 7.1 Hz, 6H). ^{13}C NMR (100 MHz, $CDCl_3$) δ 189.74, 168.21, 151.46, 131.04, 123.17, 110.11, 60.99, 45.29, 44.43, 14.01, 12.35. IR (ZnSe): 2976.01, 2933.49, 1740.70, 1661.3, 1595.93 cm^{-1}. HRMS (ESI): calculated for $C_{15}H_{22}NO_3$ (M^{+}+1) 264.1594, found 264.1596.

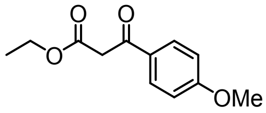

2c

1-ethoxy-3-(4'-methoxyphenyl)propane-1,3-dione (2c)

90% yield. Greenish yellow liquid. R_f = 0.43 (EtOAc/Hex = 1/4). ^{1}H NMR (400 MHz, $CDCl_3$) δ 7.93 (d, J = 9.0 Hz, 2H), 6.95 (d, J = 9.0 Hz, 2H), 4.21 (q, J = 7.1 Hz, 2H), 3.94 (s, 2H), 3.88 (s, 3H), 1.26 (t, J = 7.2 Hz, 3H). ^{13}C NMR (100 MHz, $CDCl_3$) δ 190.97, 167.70, 163.95, 130.83, 129.07, 113.89, 113.85, 85.64, 61.29, 55.46, 55.32, 45.72, 14.04. IR (ZnSe): 2980.89, 2983.45, 1740.37, 1677.63, 1601.71 cm^{-1}. HRMS (ESI): calculated for $C_{12}H_{14}NaNO_4$ (M^++23) 245.0784, found 245.0789.

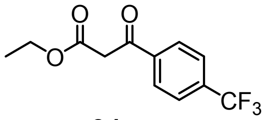

2d

1-ethoxy-3-(4'-trifluoromethylphenyl)propane-1,3-dione (2d)

71% yield. Light brown liquid. R_f = 0.50 (EtOAc/Hex = 1:1). ^{1}H NMR (400 MHz, $CDCl_3$) δ 7.80 (d, J = 8.2 Hz, 2H), 7.59 (d, J = 8.2 Hz, 2H), 4.22 (q, J = 7.2 Hz, 2H), 3.97 (s, 2H), 1.28 (t, J = 7.1 Hz, 3H). ^{13}C NMR (100 MHz, $CDCl_3$) δ 191.69, 172.92, 169.42, 167.04, 138.72, 136.85, 135.31, 134.99, 134.66, 134.34, 133.15, 132.82, 132.50, 132.17, 128.88, 127.87, 127.60, 126.36, 125.85, 125.81, 125.77, 125.74, 125.53, 125.49, 125.45, 125.41, 125.17, 124.89, 122.46, 122.18, 119.75, 119.47, 88.98, 61.61, 60.63, 45.99, 14.10, 13.91. ^{19}F NMR (376 MHz, $CDCl_3$) δ -63.15. IR (ZnSe): 2986.16, 1744.29, 1696.07, 1637.60, 1616.15 cm^{-1}. HRMS (ESI): calculated for $C_{12}H_{11}F_3NaNO_3$ (M^++23) 283.0552, found 283.0550.

General procedure for the synthesis of **3a**, **3b**, **3c**, and **3d**

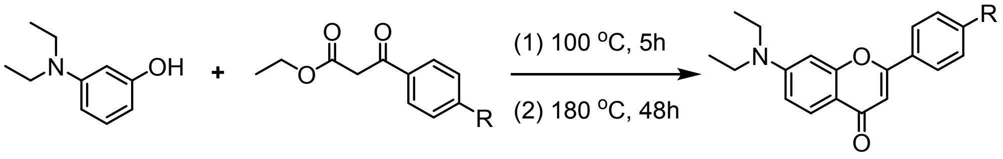

1

2a, R = H,
2b, R = NEt_2,
2c, R = OMe,
2d, R = CF_3,

3a, R = H,
3b, R = NEt_2,
3c, R = OMe,
3d, R = CF_3,

Under an inert atmosphere, a mixture of **1** (1.74 g, 11 mmol, 1.0 equiv.) and **2a**, or **2b**, or **2c**, or **2d** (19 mmol, 1.8 equiv.) was stirred at 100 °C for 5 h followed by an additional stirring at 180 °C for 48 h. The resulting crude product was further purified by flash column chromatography to yield flavone scaffolds (**3a-d**).

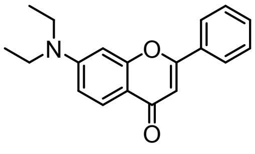

3a

7-(diethylamino)-2-phenyl-4H-chromen-4-one (3a)

25% yield. Brownish yellow solid, m.p. 136-137 °C. R_f = 0.38 (Acetone/DCM = 1/19). ^{1}H NMR (400 MHz, $CDCl_3$) δ 7.98 (d, J = 9.1 Hz, 1H), 7.88-7.84 (m, 2H), 7.46-7.44 (m, 3H), 6.69 (dd, J = 9.1, 2.5 Hz, 1H), 6.64 (s, 1H), 6.52 (d, J = 2.5 Hz, 1H), 3.41 (q, J = 7.1 Hz, 4H), 1.20 (t, J = 7.1 Hz, 6H). ^{13}C NMR (100 MHz, $CDCl_3$) δ 177.64, 162.05, 158.78, 152.02, 132.35, 130.97, 128.88, 126.75, 126.03, 113.18, 110.52, 107.25, 96.43, 44.74, 12.52. IR (ZnSe): 2972.54, 2930.24, 1624.59, 1600.42, 1405.59 cm^{-1}. HRMS (ESI): calculated for $C_{19}H_{20}NO_2$ (M^++1) 294.1489, found 294.1490.

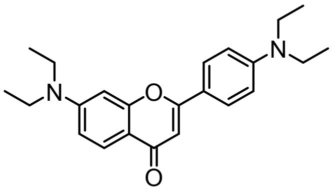

3b

7-(diethylamino)-2-(4-(diethylamino)phenyl)-4H-chromen-4-one (3b)

51% yield. Yellow solid, m.p. 159-160 °C. R_f = 0.45 (Acetone/DCM = 1/9). ^{1}H NMR (400 MHz, $CDCl_3$) δ 7.98 (d, J = 9.0 Hz, 1H), 7.75 (d, J = 9.0 Hz, 2H), 6.70-6.66 (m, 3H), 6.53 (s, 2H), 3.45-3.37 (m, 8H), 1.23-1.17 (m, 12H). ^{13}C NMR (100 MHz, $CDCl_3$) δ 177.84, 163.23, 158.67, 151.71, 149.76, 127.70, 126.65, 118.20, 113.33, 111.13, 110.05, 103.81, 96.59, 44.74, 44.55, 12.60. IR (ZnSe): 2972.54, 1624.59, 1600.42, 1518.47 cm^{-1}. HRMS (ESI): calculated for $C_{23}H_{29}N_2O_2$ (M^++1) 365.2224, found 365.2222.

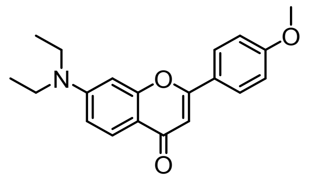

3c

7-(diethylamino)-2-(4-methoxyphenyl)-4H-chromen-4-one (3c)

46% yield. Brownish solid, m.p. 154-155 °C. R_f = 0.5 (Acetone/DCM = 3/17). ^{1}H NMR (400 MHz, $CDCl_3$) δ 7.98 (d, J = 9.1 Hz, 1H), 7.81 (d, J = 9.0 Hz, 2H), 6.96 (d, J = 9.0 Hz, 2H), 6.68 (dd, J = 9.1, 2.5 Hz, 1H), 6.56 (s, 1H), 6.51 (d, J = 2.5 Hz, 1H), 3.83 (s, 3H), 3.42 (q, J = 7.1 Hz, 4H), 1.21 (t, J = 7.1 Hz, 6H). ^{13}C NMR (100 MHz, $CDCl_3$) δ 177.72, 162.14, 161.94, 158.73, 151.94, 127.71, 126.74, 124.74, 114.32, 113.19, 110.38, 105.90, 96.49, 55.49, 44.77, 12.57. IR (ZnSe): 2972.91, 1628.38, 1606.71, 1524.64 cm^{-1}. HRMS (ESI): calculated for $C_{20}H_{22}NO_3$ (M^++1) 324.1594, found 324.1595.

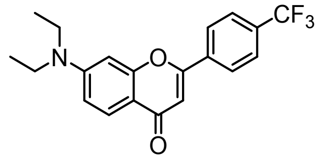

3d

7-(diethylamino)-2-(4-(trifluoromethyl)phenyl)-4H-chromen-4-one (3d)

48% yield. Brownish yellow solid, m.p. 203-204 °C. R_f = 0.3 (Acetone/DCM = 3/17). ^{1}H NMR (400 MHz, $CDCl_3$) δ 7.91-7.87 (m, 3H), 7.63 (d, J = 8.3 Hz, 2H), 6.62 (dd, J = 9.1, 2.3 Hz, 1H), 6.60 (s, 1H), 6.46 (d, J = 2.3 Hz, 1H), 3.37 (q, J = 7.1 Hz, 4H), 1.17 (t, J = 7.1 Hz, 6H). ^{13}C NMR (100 MHz, $CDCl_3$) δ 177.03, 159.99, 158.59, 152.11, 135.61, 132.45 (q, J = 32.4 Hz, $^2J_{CF}$), 126.67, 126.19, 125.68 (q, J = 3.8 Hz, $^3J_{CF}$), 123.69 (q, J = 270.7 Hz, $^1J_{CF}$) 113.00, 110.66, 108.30, 96.22, 44.67, 12.39. ^{19}F NMR (376 MHz, $CDCl_3$) δ -62.91. IR (ZnSe): 3069.05, 2973.86, 1627.06, 1595.91 cm^{-1}. HRMS (ESI): calculated for $C_{20}H_{19}F_3NO_2$ (M^++1) 362.1362, found 362.1362.

Synthesis of **5**

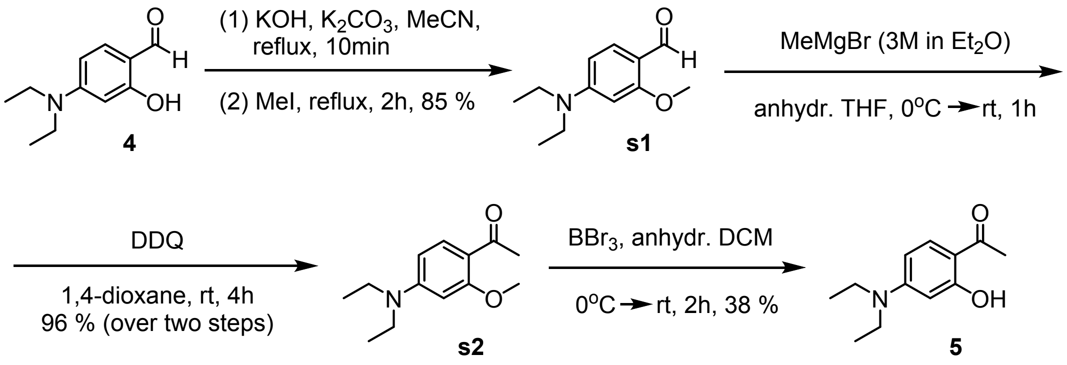

4-(diethylamino)-2methoxybenzaldehyde (s1)

A solution of MeCN (25 mL), 4-(diethylamino)-2-hydroxybenzaldehyde (5.8 g, 30.4 mmol, 1.0 equiv.), KOH (2 g, 35.7 mmol, 1.2 equiv.), and K_2CO_3 (8.3 g, 60 mmol, 2.0 equiv.) was refluxed for 10 min followed by the addition of MeI (3.5 mL, 56 mmol, 1.9 equiv.), and a two-hour reflux. The reaction was cooled to room temperature, and filtrated to remove the precipitate. The filtrate was concentrated and dissolved in DCM (200 mL) and washed with water three times (50 mL*3). Subsequently, the organic layer was collected, dried over $MgSO_4$, concentrated, and purified by flash column chromatography to yield **s1** as a yellow solid (5.3 g, 25.7 mmol, 85%). m.p. 91-93 °C. R_f = 0.20 (EtOAc/Hex = 1/4). ^{1}H NMR (400 MHz, $CDCl_3$) δ 10.08 (d, J = 0.8 Hz, 1H), 7.64 (d, J = 9.0 Hz, 1H), 6.22 (ddd, J = 9.0, 2.3, 0.8 Hz, 1H), 5.98 (d, J = 2.3 Hz, 1H), 3.83 (s, 3H), 3.37 (q, J = 7.1 Hz, 4H), 1.17 (t, J = 7.1 Hz, 6H). ^{13}C NMR (100 MHz, $CDCl_3$) δ 186.90, 164.09, 153.87, 130.58, 114.13, 104.19, 92.38, 55.16, 44.73, 12.52. IR (ZnSe): 2973.05, 2933.42, 2839.22, 1653.67, 1595.79, 1533.98, 1522.56 cm^{-1}. HRMS (ESI): calculated for $C_{12}H_{18}NO_2$ (M^{+}+1) 208.1332, found 208.1332.

1-(4-(diethylamino)-2-methoxyphenyl)ethan-1-one (s2)

To a solution of **s1** (4g, 19.3 mmol, 1.0 equiv.) in anhydrous THF (80 mL) was added 3M MeMgBr in Et_2O (7.1 mL, 21.6 mmol, 1.1 equiv.) dropwise at 0 °C. The resulting solution was warmed up to room temperature and stirred for 1 h, then cooled to 0 °C before quenching with water. THF was then removed under the reduced pressure and the residue was then taken up in DCM (100 mL) and washed with water (100 mL). The organic layer was dried with $MgSO_4$ and concentrated. The resulting residue was dissolved in 1,4-dioxane (4.5 mL) and added to a solution of DDQ (5.88 g, 25.6 mmol, 1.3 equiv.) in 1,4-dioxane (4.5 mL). The resulting mixture was stirred at room temperature for 4 h and subsequently filtered, concentrated, and finally purified by flash column chromatography to afford **s2** as a yellow solid (4.1g, 18.5 mmol, 96% over two steps). m.p. 74-76 °C. R_f = 0.33 (EtOAc/Hex = 3/7). ^{1}H NMR (400 MHz, $CDCl_3$) δ 7.77

(d, J = 9.0 Hz, 1H), 6.22 (dd, J = 9.0, 2.4 Hz, 1H), 6.03 (d, J = 2.4 Hz, 1H), 3.85 (s, 3H), 3.36 (q, J = 7.1 Hz, 4H), 2.50 (s, 3H), 1.16 (t, J = 7.1 Hz, 6H). ^{13}C NMR (100 MHz, $CDCl_3$) δ 196.17, 161.93, 152.56, 132.78, 115.31, 103.81, 93.12, 54.99, 44.55, 31.60, 12.53. IR (ZnSe): 2972.59, 2931.56, 1646.53, 1595.03, 1547.93, 1518.56, 1400.78 cm^{-1}. HRMS (ESI): calculated for $C_{13}H_{20}NO_2$ (M^++1) 222.1489, found 222.1486.

1-(4-(diethylamino)-2-hydroxyphenyl)ethan-1-one (5)

To a solution of **s2** (1.2 g, 5.4 mmol, 1.0 equiv.) in anhydrous DCM (25 mL) was added 1M BBr_3 (6 mL, 6 mmol, 1.1 equiv.) at 0 °C followed by stirring at room temperature for 2 h. The resulting mixture was poured into iced water and stirred for additional 30 min followed by extraction with DCM (50 mL*3) and water (50 mL). The combined organic layers were collected and dried over $MgSO_4$, concentrated, purified by flash column chromatography to afford **5** as a colorless liquid (417 mg, 2 mmol, 37%). R_f = 0.38 (Hex/DCM = 2/3). ^{1}H NMR (400 MHz, $CDCl_3$) δ 12.90 (s, 1H), 7.49 (d, J = 9.1 Hz, 1H), 6.17 (dd, J = 9.1, 2.6 Hz, 1H), 6.05 (d, J = 2.6 Hz, 1H), 3.37 (q, J = 7.1 Hz, 4H), 2.45 (s, 3H), 1.18 (t, J = 7.1 Hz, 6H). ^{13}C NMR (100 MHz, $CDCl_3$) δ 200.37, 165.19, 153.90, 132.68, 109.90, 103.67, 97.17, 44.69, 25.49, 12.69. IR (ZnSe): 2973.85, 2930.65, 1632.73, 1556.27, 1523.16, 1416.54, 1375.14 cm^{-1}. HRMS (ESI): calculated for $C_{12}H_{18}NO_2$ (M^++1) 208.1332, found 208.1333.

Synthesis of **6**

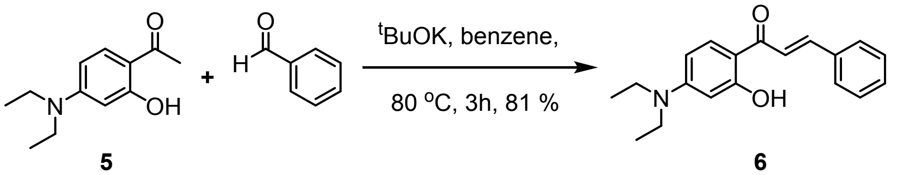

(*E*)-1-(4-(diethylamino)-2-hydroxyphenyl)-3-phenylprop-2-en-1-one (6)

A solution of **5** (32 mg, 0.15 mmol, 1.0 equiv.), benzaldehyde (0.031 mL, 0.30 mmol, 2.0 equiv.), ^{t}BuOK (40 mg, 0.36 mmol, 2.3 equiv.), and benzene (2 mL) was stirred at 80 °C for 3 h. The resulting mixture was cooled to room temperature and DCM (50 mL) and 10 % $HCl_{(aq)}$ were added sequentially followed by the extraction with DCM (50 mL*3) and water (30 mL). The combined organic layers were dried over $MgSO_4$, concentrated, purified by flash column chromatography to afford **6** as a yellow solid (36 mg, 0.12 mmol, 81%). m.p. 100-101 °C. R_f = 0.40 (Hex/DCM = 2/3). ^{1}H NMR (400 MHz, $CDCl_3$) δ 13.66 (s, 1H), 7.82 (d, J = 15.5 Hz, 1H), 7.73 (d, J = 9.3 Hz, 1H), 7.65-7.63 (m, 2H), 7.56 (d, J = 15.5 Hz, 1H), 7.44-7.39 (m, 3H), 6.26 (dd, J = 9.3, 2.6 Hz, 1H), 6.15 (d, J = 2.6 Hz, 1H), 3.42 (q, J = 7.1 Hz, 4H), 1.23 (t, J = 7.1 Hz, 6H). ^{13}C

NMR (100 MHz, $CDCl_3$) δ 189.59, 166.70, 154.03, 142.47, 135.40, 131.67, 130.18, 128.99, 128.38, 120.98, 110.34, 103.95, 97.59, 44.78, 12.78. IR (ZnSe): 3500, 2973.63, 1629.95, 1558.51, 1520.08, 1372.71 cm^{-1}. HRMS (ESI): calculated for $C_{19}H_{22}NO_2$ $(M^{+}+1)$ 296.1645, found 296.1647.

Synthesis of **3a**

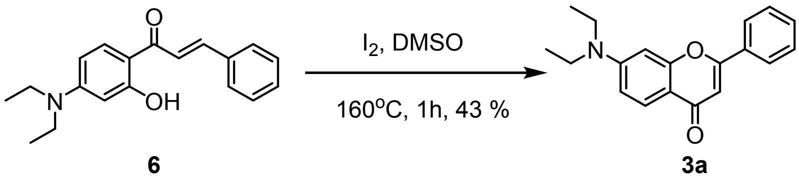

7-(diethylamino)-2-phenyl-4H-chromen-4-one (3a)

A solution of **6** (139 mg, 0.47 mmol, 1.0 equiv.), iodine (7.2 mg, 0.028 mmol, 0.06 equiv.), and DMSO (9 mL) was heated up to 160 °C for 1 h. The resulting solution was quenched with sat. $Na_2S_2O_{3(aq)}$, dissolved in DCM (100 mL), and extracted with DCM (100 mL*2) and water (50 mL*3). The combined organic layers were dried over $MgSO_4$, concentrated, and purified by flash column chromatography to yield **3a** as a brownish yellow solid (57.5 mg, 0.20 mmol, 43%). Brownish yellow solid, m.p. 136-137 °C. R_f = 0.38 (Acetone/DCM = 1/19). ^{1}H NMR (400 MHz, $CDCl_3$) δ 7.98 (d, J = 9.1 Hz, 1H), 7.88-7.84 (m, 2H), 7.46-7.44 (m, 3H), 6.69 (dd, J = 9.1, 2.5 Hz, 1H), 6.64 (s, 1H), 6.52 (d, J = 2.5 Hz, 1H), 3.41 (q, J = 7.1 Hz, 4H), 1.20 (t, J = 7.1 Hz, 6H). ^{13}C NMR (100 MHz, $CDCl_3$) δ 177.64, 162.05, 158.78, 152.02, 132.35, 130.97, 128.88, 126.75, 126.03, 113.18, 110.52, 107.25, 96.43, 44.74, 12.52. IR (ZnSe): 2972.54, 2930.24, 1624.59, 1600.42, 1405.59 cm^{-1}. HRMS (ESI): calculated for $C_{19}H_{20}NO_2$ $(M^{+}+1)$ 294.1489, found 294.1490.

A general procedure for the synthesis of **H-NSSF**, **N-NSSF**, **O-NSSF**, and **F-NSSF**

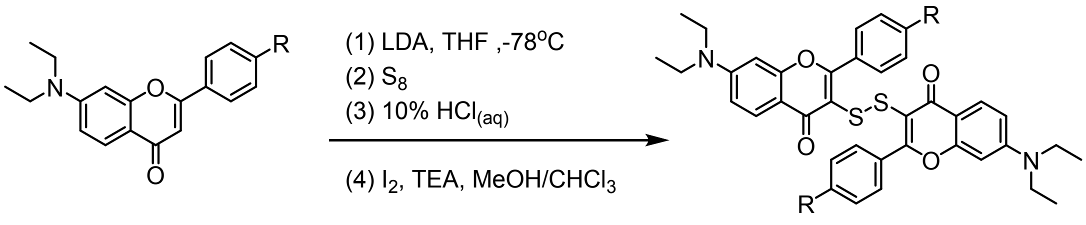

**3a,**	R = H,	**H-NSSF,**	R = H,
**3b,**	R = NEt_2,	**N-NSSF,**	R = NEt_2,
**3c,**	R = OMe,	**O-NSSF,**	R = OMe,
**3d,**	R = CF_3,	**F-NSSF,**	R = CF_3,

A solution of pre-dried diisopropylamine (0.16 mL, 1.1 mmol, 1.1 equiv.) in anhydrous THF (5 mL) was cooled to -78 °C (acetone/dry ice bath) and stirred for 10 min, followed by the addition of ^{n}BuLi (2.39 M in hexanes, 0.46 mL, 1.1 mmol, 1.1 equiv.) within 5 min and stirred for additional 10 min at -78 °C. To this freshly prepared LDA solution, anhydrous THF (2.5 mL) solution of **3a**, or **3b**, or **3c**, or **3d** was added dropwise within 5 min, respectively, and stirred for additional 30 min at -78 °C. Subsequently, sulfur powder (32.57 mg, 1.1 mmol, 1.1 equiv.) was added to the reaction mixture at -78 °C. The resulting mixture was then warmed up to room temperature and poured into degassed water, followed by washing with degassed diethyl ether (50 mL). The water layer was acidified by 10% $HCl_{(aq)}$. The murky water layer was then extracted with degassed EtOAc (50 mL*3). The combined organic layers were dried over $MgSO_4$ and concentrated under the reduced pressure to obtain a mixture of thiol flavones and disulfide flavones. The resulting mixture was dissolved in 50% MeOH/$CHCl_3$ containing 1 mL trimethylamine. Subsequently, saturated iodine in MeOH was added dropwise at room temperature and monitored by TLC until the completion of the reaction. The reaction mixture was then washed with $Na_2S_2O_{3(aq)}$ (50 mL*3), and extracted with DCM (50 mL*2). The combined organic layers were dried over $MgSO_4$, concentrated under the reduced pressure, and purified by flash column chromatography to afford disulfide flavones.

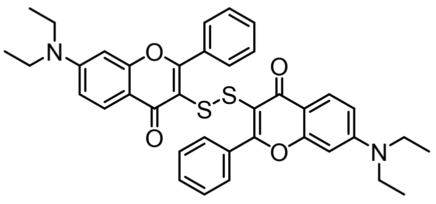

H-NSSF

3,3'-disulfanediylbis(7-(diethylamino)-2-phenyl-4H-chromen-4-one) (H-NSSF)

58% yield. Brown solid, m.p. 230-231 ^{o}C. R_f = 0.36 (MeOH/DCM = 1/19). ^{1}H NMR (400 MHz, $CDCl_3$) δ 7.87 (d, J = 9.0 Hz, 1H), 7.54 – 7.51 (m, 2H), 7.10 – 7.06 (m, 3H), 6.70 (dd, J = 9.0, 2.4 Hz, 1H), 6.40 (d, J = 2.4 Hz, 1H), 3.47 (q, J = 7.1 Hz, 4H), 1.26 (t, J = 7.1 Hz, 6H). ^{13}C NMR (100 MHz, $CDCl_3$) δ 173.87, 166.56, 158.01, 151.93, 132.76, 130.04, 129.50, 127.71, 127.31, 117.34, 112.08, 110.53, 96.11, 44.82, 29.65, 12.52. IR (ZnSe): 2973.43, 2928.70, 1637.08, 1614.25, 1566.93, 1522.62 cm^{-1}. HRMS (ESI): calculated for $C_{38}H_{37}N_2O_4S_2$ (M^{+}+1) 649.2189, found 649.2211.

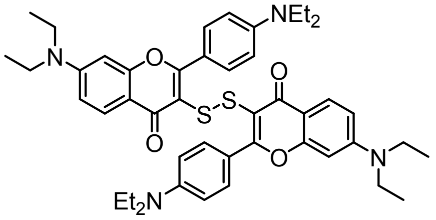

N-NSSF

3,3'-disulfanediylbis(7-(diethylamino)-2-(4-(diethylamino)phenyl)-4H-chromen-4-one) (N-NSSF)

43% yield. Yellow solid, m.p. 252-253 ^{o}C. R_f = 0.28 (Acetone/DCM = 3/17). ^{1}H NMR (400 MHz, $CDCl_3$) δ 7.87 (d, J = 9.0 Hz, 1H), 7.56 (d, J = 9.0 Hz, 2H), 6.59 (dd, J = 9.1, 2.4 Hz, 1H), 6.35 (d, J = 2.4 Hz, 1H), 6.25 (d, J = 9.1 Hz, 2H), 3.42 (q, J = 7.1 Hz, 4H), 3.16 (q, J = 7.1 Hz, 4H), 1.23 (t, J = 7.0 Hz, 6H), 1.06 (t, J = 7.1 Hz, 6H). ^{13}C NMR (100 MHz, $CDCl_3$) δ 174.75, 167.24, 158.02, 151.62, 148.88, 131.50, 127.81, 119.32, 115.08, 112.48, 109.91, 109.66, 96.18, 44.71, 44.42, 12.79, 12.70. IR (ZnSe): 2971.94, 2929.34, 1611.25, 1522.37, 1498.39 cm^{-1} . HRMS (ESI): calculated for $C_{46}H_{55}N_4O_4S_2$ (M^{+}+1) 791.3659, found 791.3683.

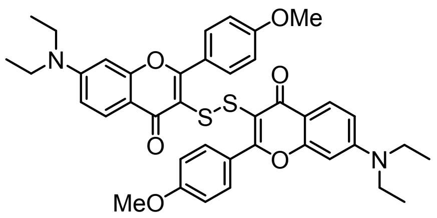

O-NSSF

3,3'-disulfanediylbis(7-(diethylamino)-2-(4-methoxyphenyl)-4H-chromen-4-one) (O-NSSF)

33 % yield. Brownish yellow solid, m.p. 150 – 152 °C. R_f = 0.22 (MeOH/DCM = 1/49). ^{1}H NMR (400 MHz, $CDCl_3$) δ 7.82 (d, J = 9.0 Hz, 1H), 7.54 (d, J = 8.6 Hz, 2H), 6.64 (dd, J = 9.0, 1.80 Hz, 1H), 6.52 (d, J = 8.6 Hz, 2H), 6.35 (d, J = 1.80 Hz, 1H), 3.61 (s, 3H), 3.45 (q, J = 7.0 Hz, 4H), 1.26 (t, J = 7.0 Hz, 6H). ^{13}C NMR (100 MHz, $CDCl_3$) δ 174.13, 166.22, 160.94, 157.97, 151.94, 131.42, 127.61, 125.18, 116.56, 112.76, 112.14, 110.41, 96.13, 55.08, 44.88, 12.66. IR (ZnSe): 2974.01, 2931.45, 1613.49, 1578.24, 1566.77, 1522.24, 1498.96 cm^{-1}. HRMS (ESI): calculated for $C_{40}H_{41}N_2O_6S_2$ (M^++1) 709.2401, found 709.2409.

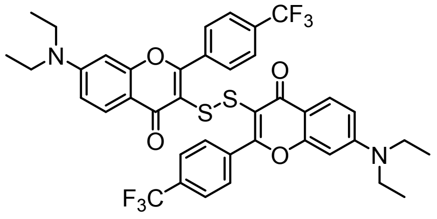

F-NSSF

3,3'-disulfanediylbis(7-(diethylamino)-2-(4-(trifluoromethyl)phenyl)-4H-chromen-4-one) (F-NSSF)

66% yield. Yellow solid, m.p. 245-246 °C. R_f = 0.35 (MeOH/DCM = 2/23). ^{1}H NMR (400 MHz, $CDCl_3$) δ 7.79 (d, J = 9.1 Hz, 1H), 7.63 (d, J = 8.1 Hz, 2H), 7.29 (d, J = 8.1 Hz, 2H), 6.69 (dd, J = 9.1, 2.4 Hz, 1H), 6.36 (d, J = 2.4 Hz, 1H), 3.46 (q, J = 7.1 Hz, 4H), 1.26 (t, J = 7.1 Hz, 6H). ^{13}C NMR (100 MHz, $CDCl_3$) δ 173.26, 164.97, 157.99, 152.36, 136.07, 131.86 (q, J = 32.5 Hz, $^2J_{CF}$), 131.70, 130.17, 127.79, 124.52 (q, J = 3.3 Hz, $^3J_{CF}$), 118.11, 111.90, 111.34, 95.64, 44.91, 12.50. ^{19}F NMR (376 MHz, CDCl3) δ -62.80. IR (ZnSe): 2977.11, 2932.28, 1614.63, 1582.76, 1525.23 cm^{-1}. HRMS (ESI): calculated for $C_{40}H_{35}F_6N_2O_4S_2$ (M^++1) 785.1937, found 785.2012.

A general procedure for the synthesis of **H-NTF**, **N-NTF**, **O-NTF**, and **F-NTF**

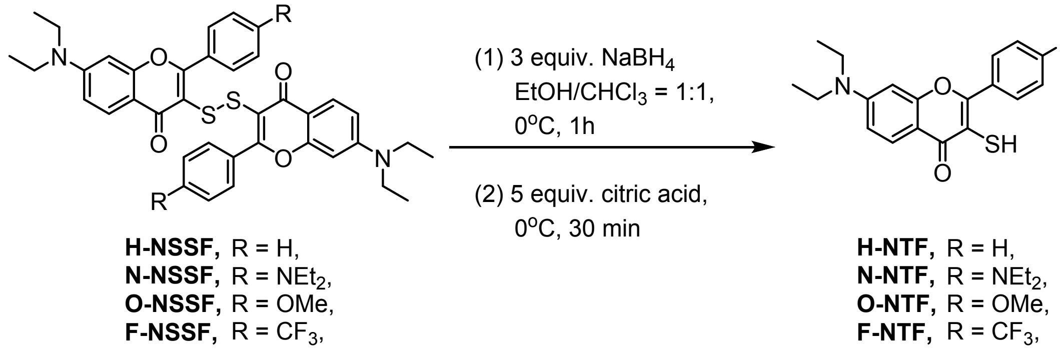

To a solution of disulfide (0.071 mmol, 1.0 equiv.) in 50 % EtOH/$CHCl_3$ (2 mL) was added $NaBH_4$ (8.0 mg, 0.212 mmol, 3.0 equiv.) at 0 °C and stirred for 1 h. Subsequently, citric acid (67.8 mg, 0.353 mmol, 5.0 equiv.) was added to the solution at 0 °C and stirred for additional 30 min. The resulting mixture was extracted with degassed EtOAc (3 mL) and degassed Water (2 mL). The combined organic layers were washed with degassed $NH_4Cl_{(aq)}$ (1 mL) and degassed $NaCl_{(aq)}$ (1 mL) sequentially and then dried over $MgSO_4$, and concentrated. Recrystallization was carried out in EtOAc with freeze-pump-thawing degassed cycles and stored at -4 °C freezer to give the single-crystal quality of mercaptoflavones. Sublimation: 20 mg of crude **NTFs** were sublimed under 10 Pa with temperature gradient; the yellow solid deposit at 200 °C was collected and characterized.

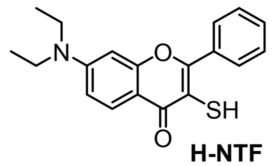

H-NTF

7-(diethylamino)-3-mercapto-2-phenyl-4H-chromen-4-one (H-NTF)
Quantitative yield. Yellow solid, m.p. 129-131 °C. R_f = 0.41 (EtOAc/Hex = 2/3). ^{1}H NMR (400 MHz, $CDCl_3$) δ 8.06 (d, J = 9.2 Hz, 1H), 7.90 (dd, J = 8.1, 1.7 Hz, 2H), 7.57-7.44 (m, 3H), 6.76 (dd, J = 9.2, 2.5 Hz, 1H), 6.47 (d, J = 2.5 Hz, 1H), 5.38 (s, 1H), 3.44 (q, J = 7.1 Hz, 4H), 1.23 (t, J = 7.1 Hz, 6H). ^{13}C NMR (100 MHz, $CDCl_3$) δ 172.11, 158.16, 155.12, 152.06, 134.10, 130.36, 128.58, 128.47, 127.74, 116.13, 111.11, 109.66, 95.88, 44.87, 12.58. IR (KBr): 2973.72, 2478.10 (S-H stretching), 1604.50 cm^{-1}. IR (ZnSe): 2972.73, 2477.60 (S-H stretching), 1604.00, 1523.49 cm^{-1}. HRMS (ESI): calculated for $C_{19}H_{20}NO_2S$ (M^++1) 326.1209, found 326.1209.

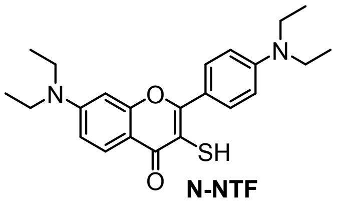

N-NTF

7-(diethylamino)-2-(4-(diethylamino)phenyl)-3-mercapto-4H-chromen-4-one (N-NTF)

Quantitative yield. Brown solid, m.p. 161-162 °C. R_f = 0.32 (EtOAc/Hex = 2/3). ^{1}H NMR (400 MHz, $CDCl_3$) δ 8.03 (d, J = 9.1 Hz, 1H), 7.84 (d, J = 9.1 Hz, 1H), 6.79 – 6.70 (m, 3H), 6.47 (d, J = 2.4 Hz, 1H), 5.45 (s, 1H), 3.44 (q, J = 7.1, 7.1 Hz, 4H), 3.43 (q, J = 7.1, 7.1 Hz, 4H), 1.23 (t, J = 7.1 Hz, 6H), 1.21 (t, J = 7.1 Hz, 6H). ^{13}C NMR (100 MHz, $CDCl_3$) δ 172.36, 157.94, 156.37, 151.76, 149.06, 130.03, 127.59, 120.09, 113.02, 110.74, 110.71, 109.89, 96.02, 44.85, 44.56, 12.71, 12.64. IR (KBr): 2969.87, 2472.32 (S-H stretching), 1600.64 cm^{-1}. IR (ZnSe): 2971.92, 2929.40, 2472.96 (S-H stretching), 1626.19, 1596.11 cm^{-1}. HRMS (ESI): calculated for $C_{23}H_{29}N_2O_2S$ (M^++1) 397.1944, found 397.1945.

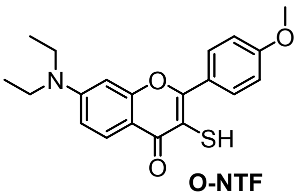

O-NTF

7-(diethylamino)-3-mercapto-2-(4-methoxyphenyl)-4H-chromen-4-one (O-NTF)

Quantitative yield. Orange solid, m.p. 110-112 °C. R_f = 0.38 (EtOAc/Hex = 2/3). ^{1}H NMR (400 MHz, $CDCl_3$) δ 8.02 (d, J = 9.1 Hz, 1H), 7.87 (d, J = 8.9 Hz, 2H), 7.02 (d, J = 8.9 Hz, 2H), 6.74 (dd, J = 9.1, 2.4 Hz, 1H), 6.45 (d, J = 2.4 Hz, 1H), 5.41 (s, 1H), 3.87 (s, 3H), 3.43 (q, J = 7.1 Hz, 4H), 1.22 (t, J = 7.1 Hz, 6H). ^{13}C NMR (100 MHz, $CDCl_3$) δ 172.14, 161.08, 158.01, 155.16, 151.95, 130.11, 127.65, 126.42, 114.97, 113.94, 110.96, 109.64, 95.88, 55.50, 44.83, 12.58. IR (KBr): 2969.87, 2478.10 (S-H stretching), 1604.50 cm^{-1}. IR (ZnSe): 2971.77, 2930.31, 2476.63 (S-H stretching), 1628.59, 1606.89, 1545.67 cm^{-1}. HRMS (ESI): calculated for $C_{20}H_{22}NO_3S$ (M^++1) 356.1320, found 356.1319.

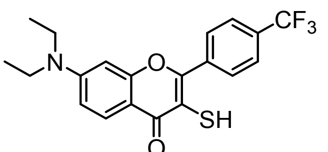

7-(diethylamino)-3-mercapto-2-(4-(trifluoromethyl)phenyl)-4H-chromen-4-one (F-NTF)

Quantitative yield. Orange solid. m.p. 154-155 °C. R_f = 0.34 (EtOAc/Hex = 3/7). ^{1}H NMR (400 MHz, $CDCl_3$) δ 8.05-8.03 (m, 3H), 7.77 (d, J = 8.3 Hz, 2H), 6.77 (dd, J = 9.2, 2.5 Hz, 1H), 6.46 (d, J = 2.4 Hz, 1H), 5.50 (s, 1H), 3.45 (q, J = 7.1 Hz, 4H), 1.24 (t, J = 7.1 Hz, 6H). ^{13}C NMR (100 MHz, $CDCl_3$) δ 171.74, 158.18, 153.11, 152.30, 137.52, 131.90 (q, J = 32.25 Hz, $^2J_{CF}$), 128.93, 127.90, 125.59 (q, J = 3.6 Hz, $^3J_{CF}$), 123.87 (d, J = 270.8 Hz, $^1J_{CF}$), 117.49, 111.39, 109.48, 95.75, 44.93, 12.58. ^{19}F NMR (376 MHz, $CDCl_3$) δ -62.91. IR (KBr): 2973.72, 2435.68 (S-H stretching), 1608.36 cm^{-1}. IR (ZnSe): 2975.14, 2929.34, 2449.39 (S-H stretching), 1629.07, 1609.79, 1587.61, 1523.49 cm^{-1}. HRMS (ESI): calculated for $C_{20}H_{19}F_3NO_2S$ (M^++1) 394.1083, found 394.1087.

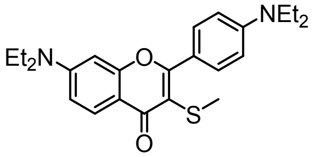

7-(diethylamino)-2-(4-(diethylamino)phenyl)-3-(methylthio)-4H-chromen-4-one (Me-N-NTF)

The synthetic method were referred to our previous report.[1] 88 % yield. Yellow solid. ^{1}H NMR (400 MHz, $CDCl_3$) δ 8.02 (d, J = 9.1 Hz, 1H), 7.78-7.70 (m, 2H), 6.74-6.67 (m, 3H), 6.45 (d, J = 2.4 Hz, 1H), 3.42 (q, J = 7.1 Hz, 8H), 2.33 (s, 3H), 1.21 (t, J = 7.1 Hz, 12H). ^{13}C NMR (101 MHz, $CDCl_3$) δ 175.74, 165.39, 158.18, 151.80, 149.32, 131.40, 127.40, 119.78, 114.81, 112.07, 110.49, 110.06, 96.19, 44.83, 44.51, 18.05, 12.69, 12.58. HRMS (ESI): calculated for $C_{24}H_{31}N_2O_2S$ (M^++1) 411.2101, found 411.2116.

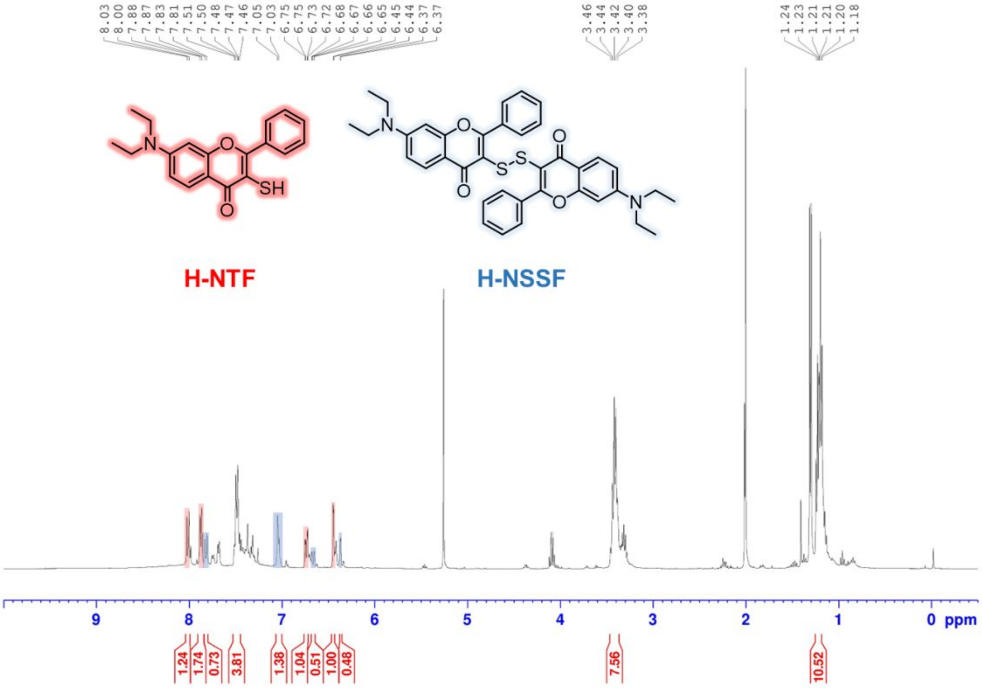

Figure S1. ^{1}H NMR spectrum in $CDCl_3$ acquired from the crude of route 1 in **scheme 1** indicating the presence of both **H-NTF** (highlighted in red color), and **H-NSSF** (highlighted in blue color) and the ratio of **H-NTF** and **H-NSSF** is 2:1.

3. Structure Characterization

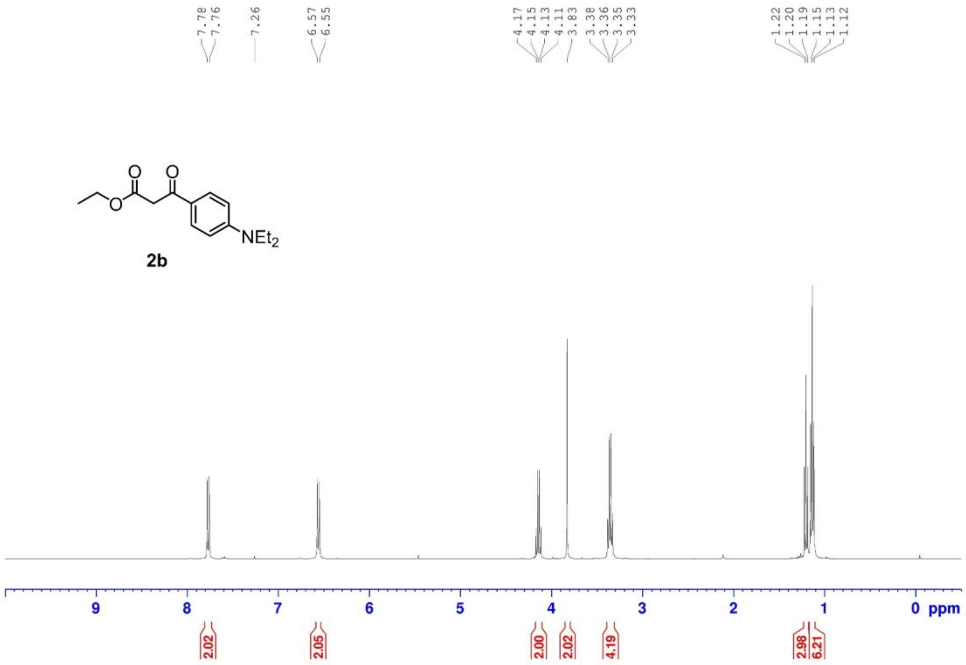

Figure S2. ^{1}H NMR of **2b** in $CDCl_3$

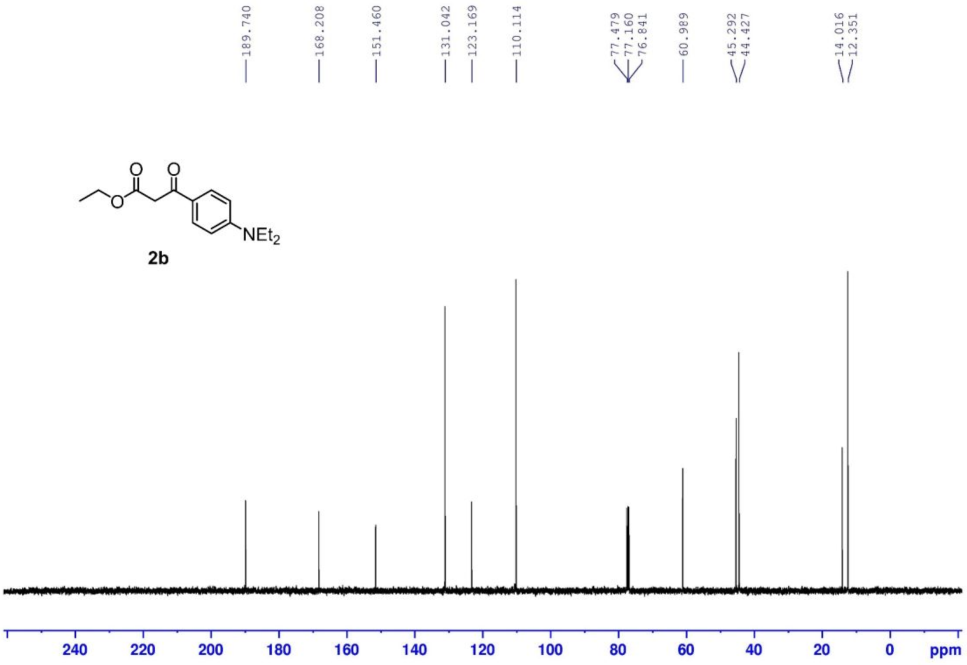

Figure S3. ^{13}C NMR of **2b** in $CDCl_3$

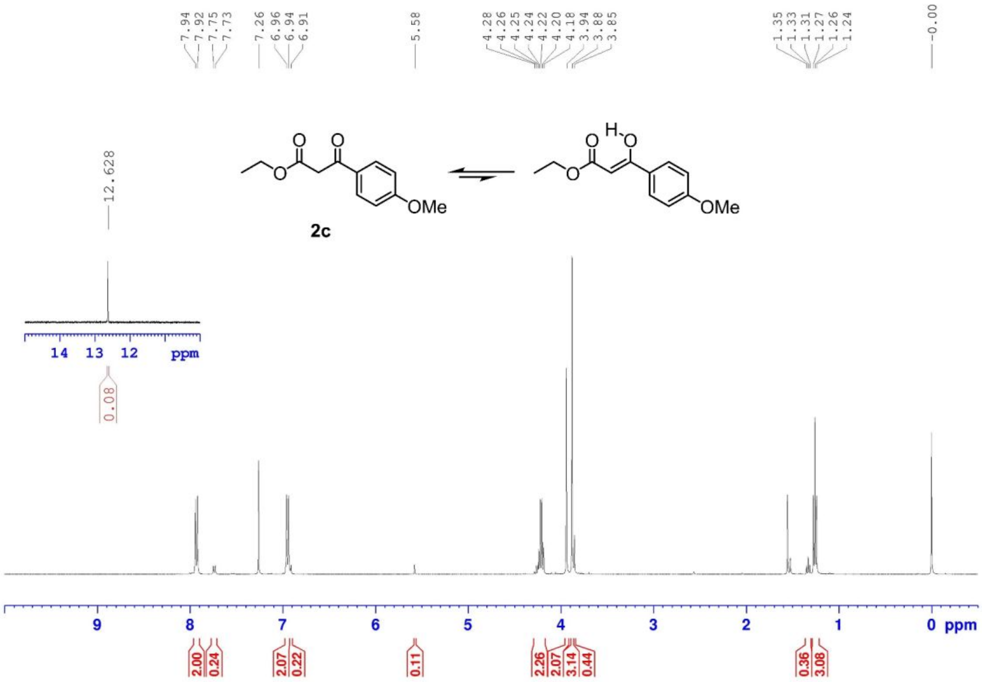

Figure S4. ^{1}H NMR of **2c** in $CDCl_3$

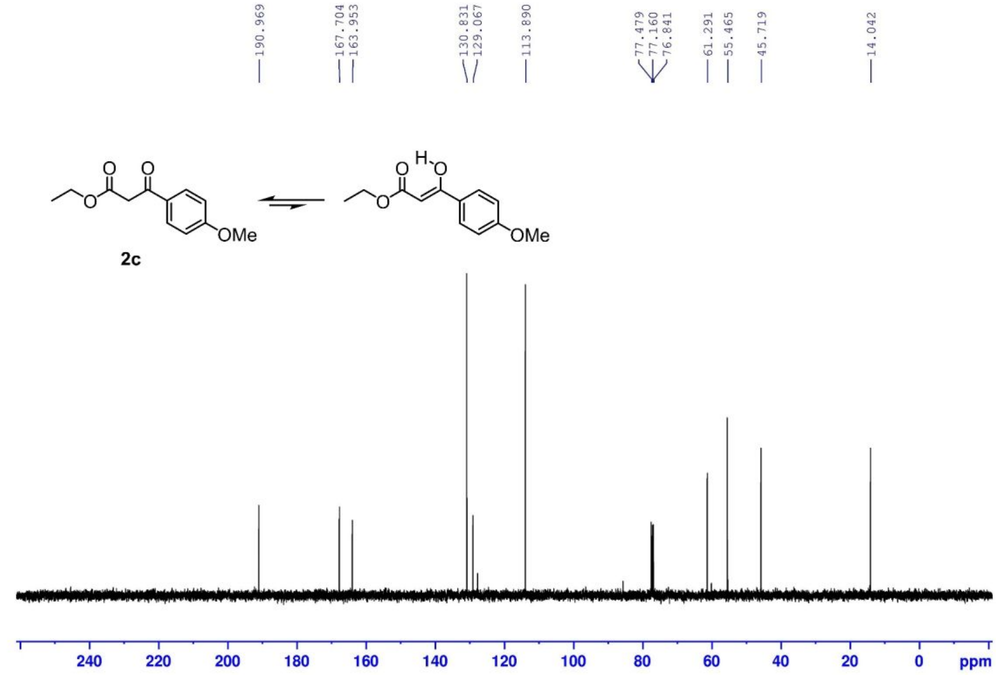

Figure S5. ^{13}C NMR of **2c** in $CDCl_3$

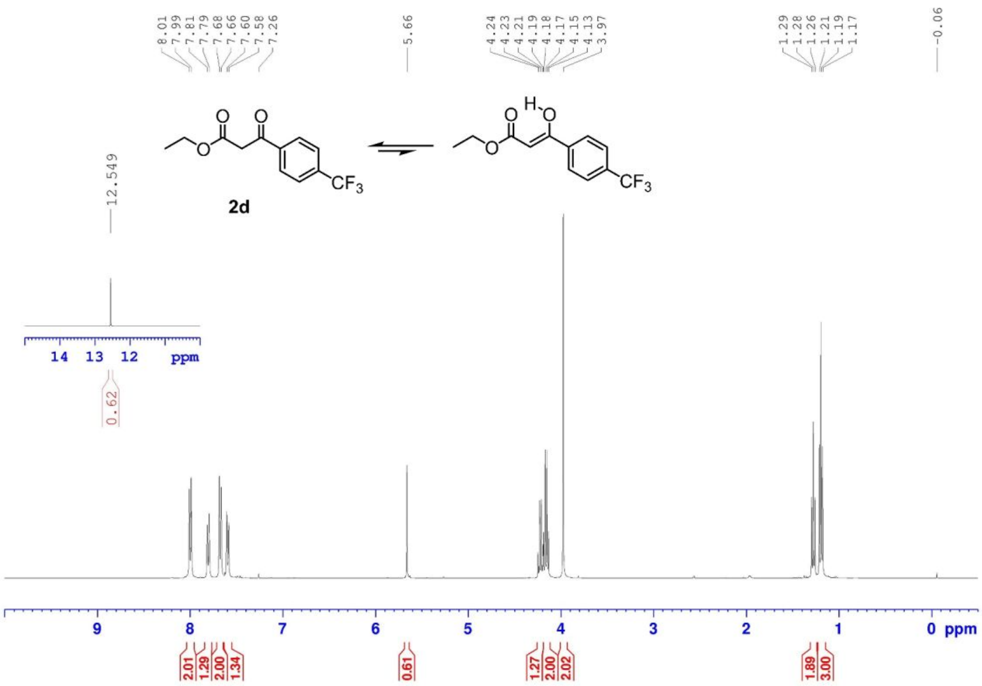

Figure S6. ^{1}H NMR of **2d** in $CDCl_3$

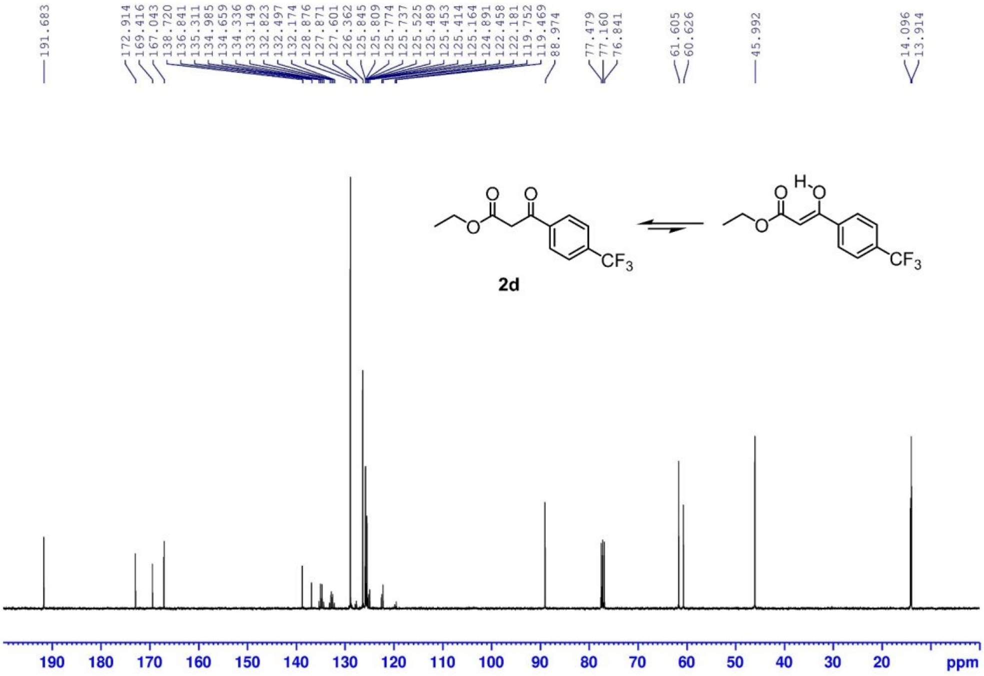

Figure S7. ^{13}C NMR of **2d** in $CDCl_3$

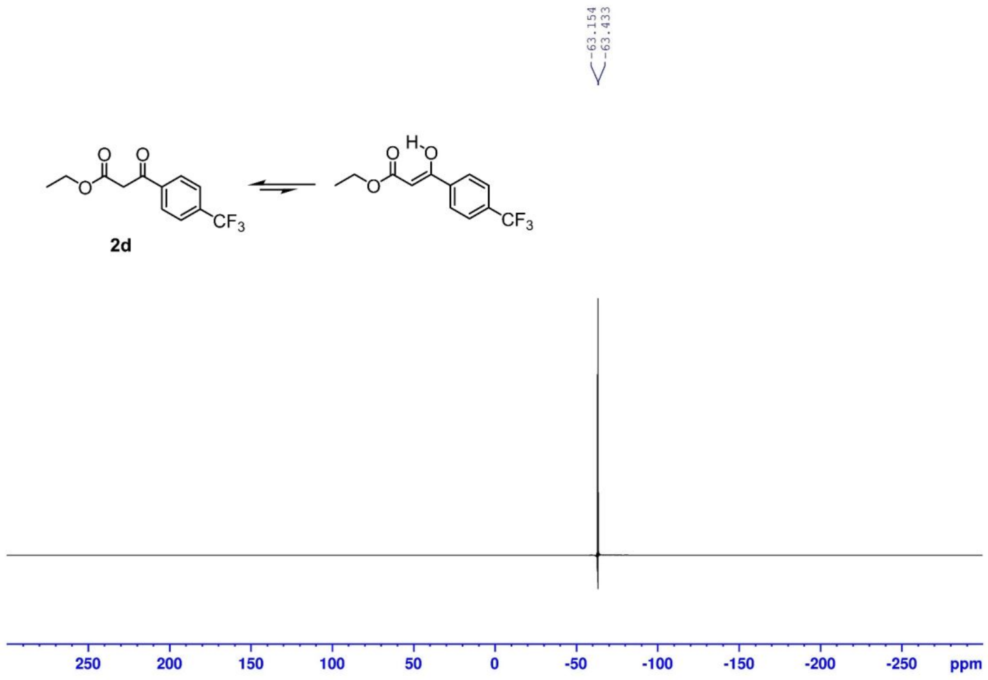

Figure S8. ^{19}F NMR of **2d** in $CDCl_3$

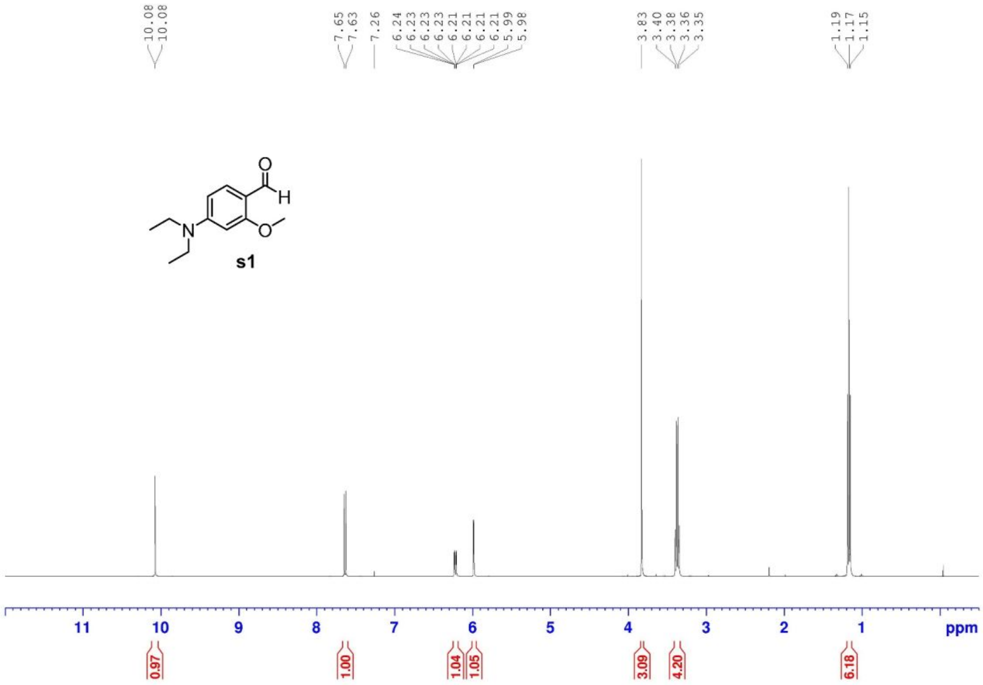

Figure S9. ^{1}H NMR of **s1** in $CDCl_3$

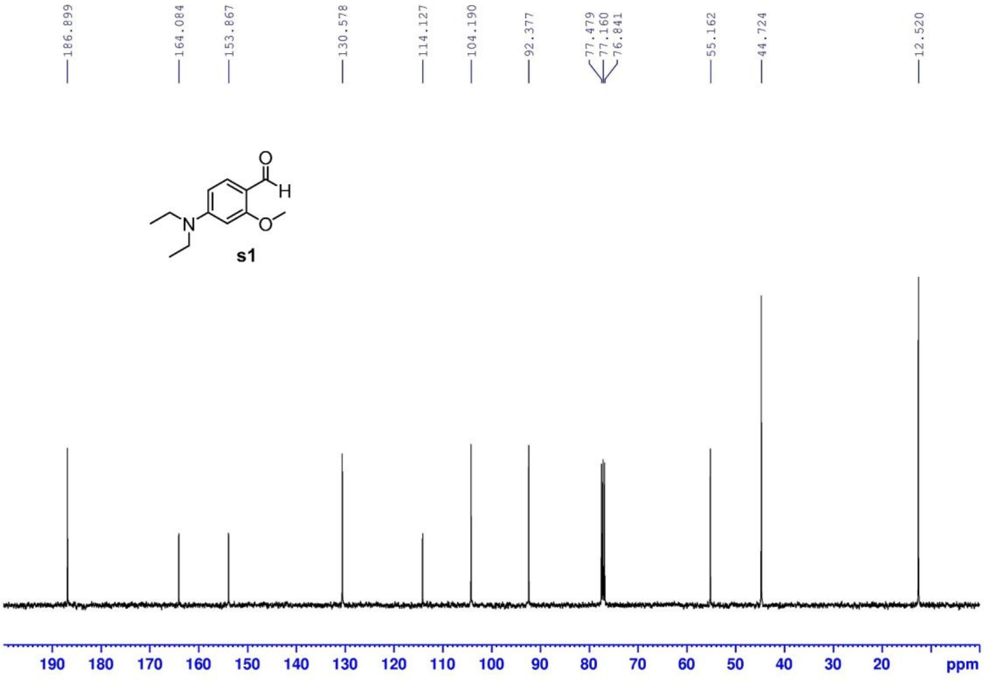

Figure S10. ^{13}C NMR of **s1** in $CDCl_3$

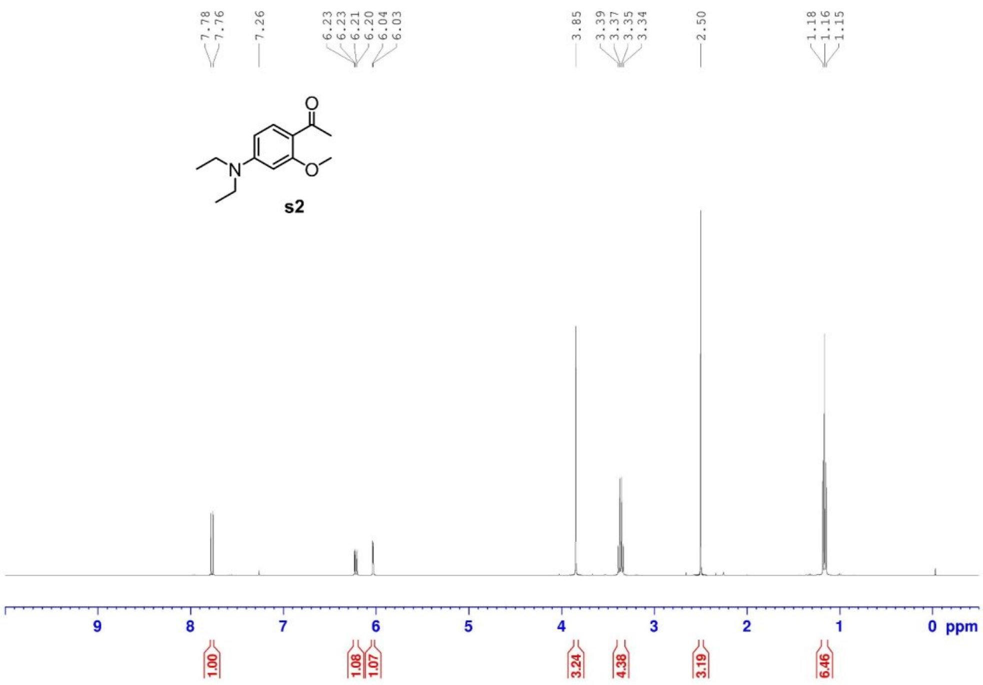

Figure S11. ^{1}H NMR of **s2** in $CDCl_3$

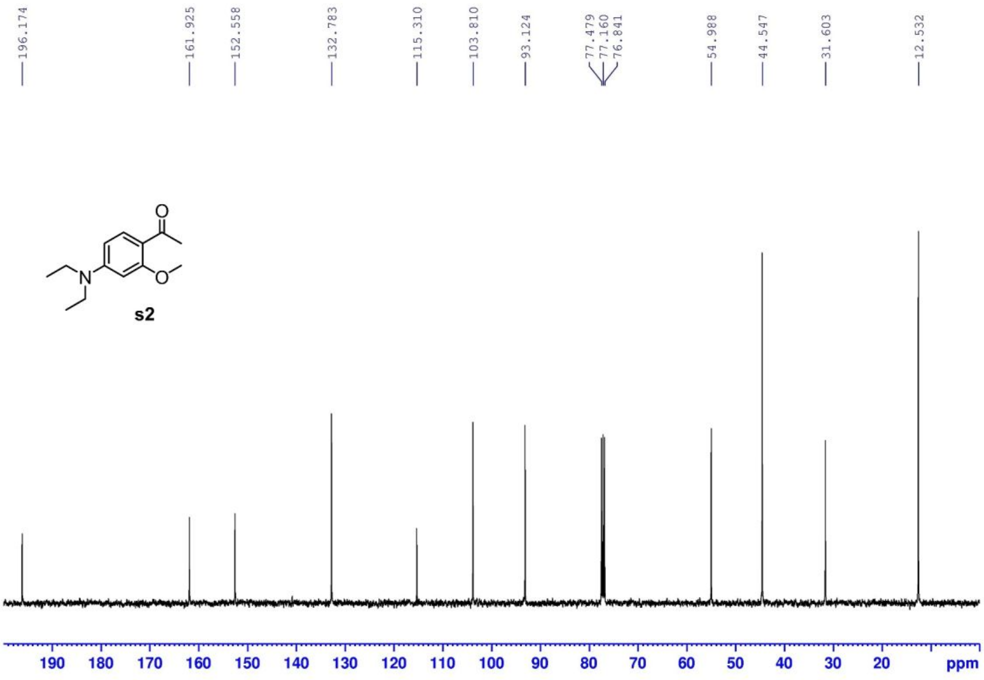

Figure S12. ^{13}C NMR of **s2** in $CDCl_3$

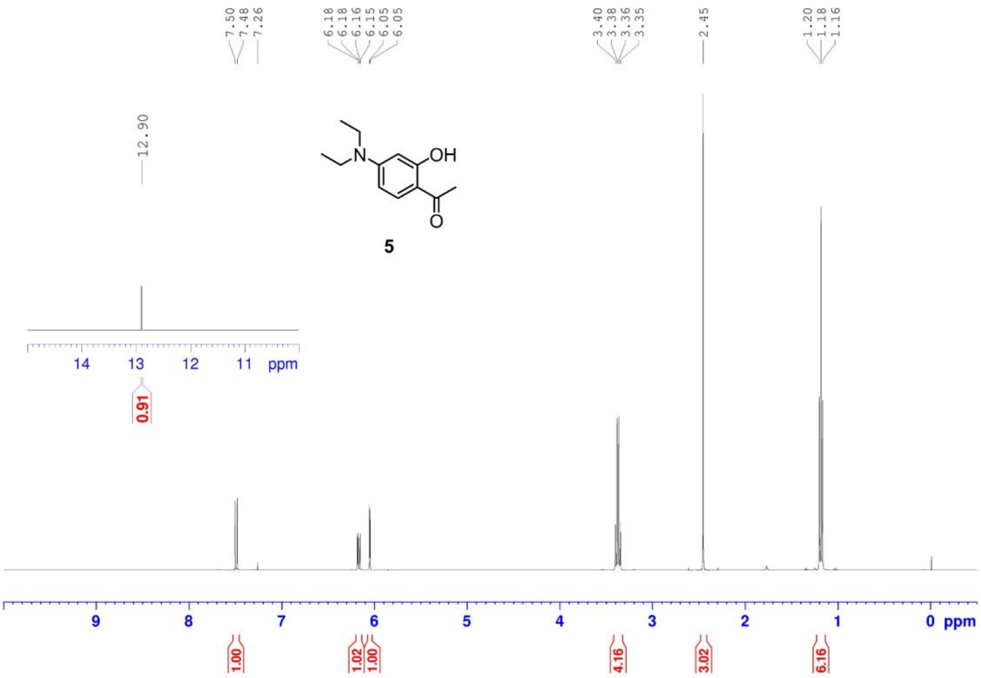

Figure S13. ^{1}H NMR of **5** in $CDCl_3$

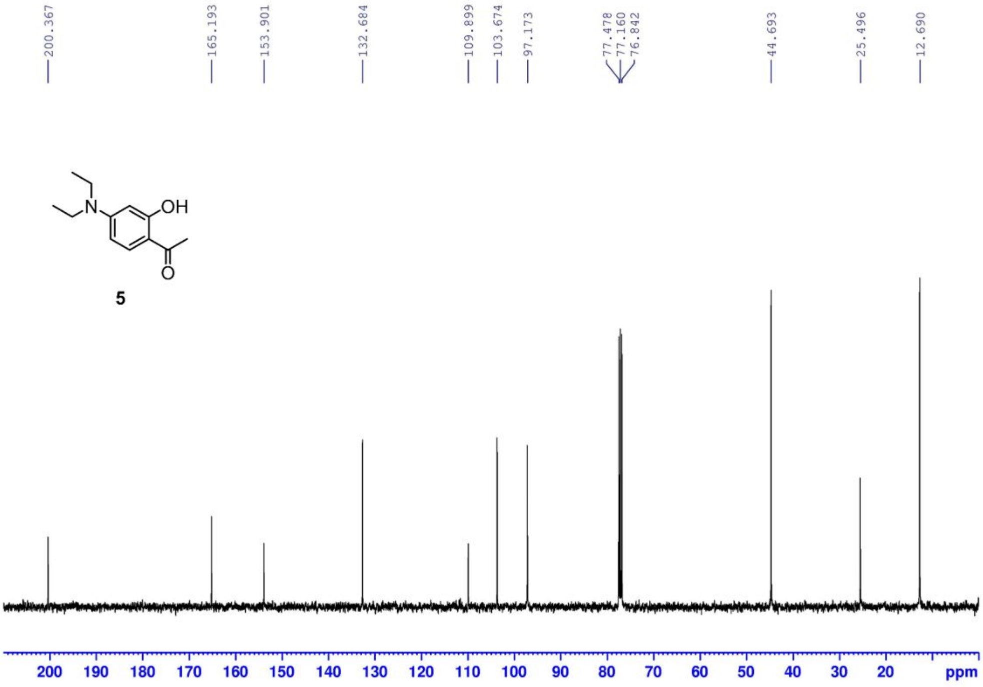

Figure S14. ^{13}C NMR of **5** in $CDCl_3$

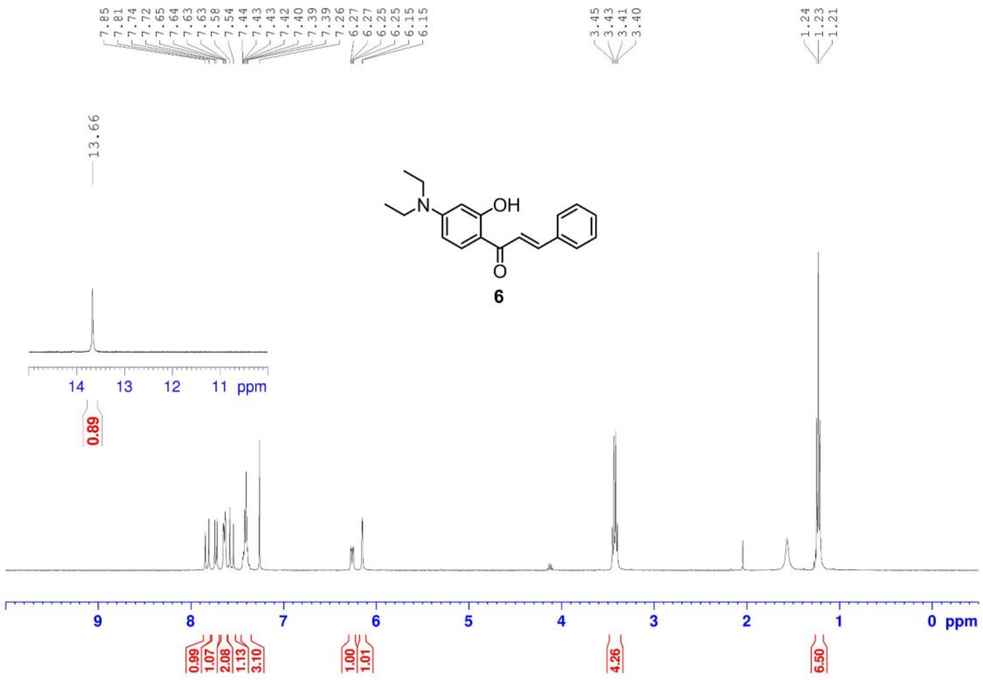

Figure S15. ^{1}H NMR of **6** in $CDCl_3$

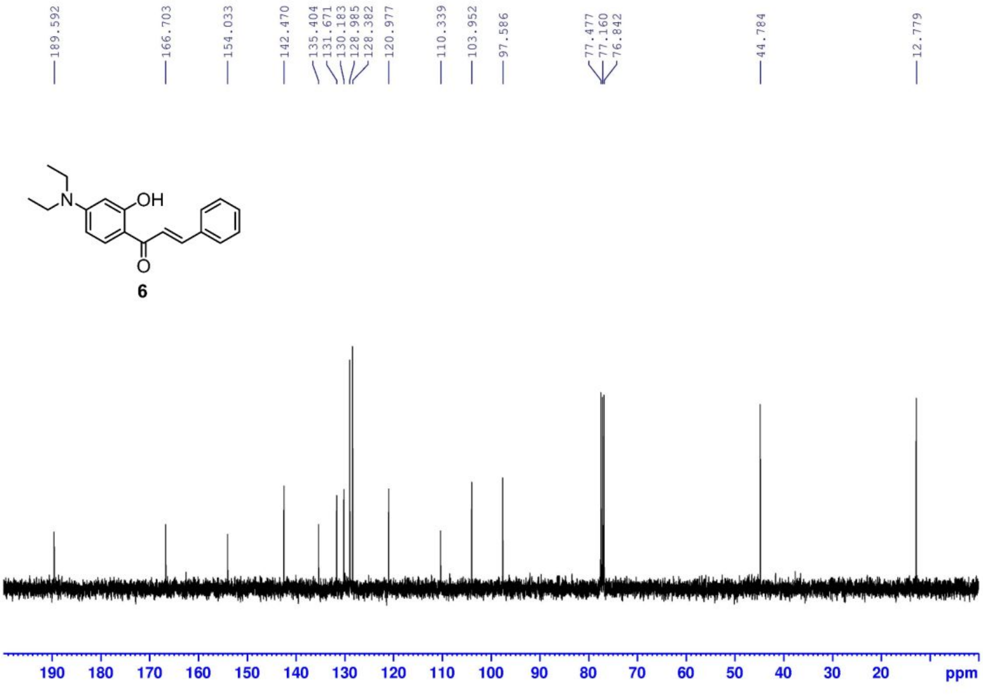

Figure S16. ^{13}C NMR of **6** in $CDCl_3$

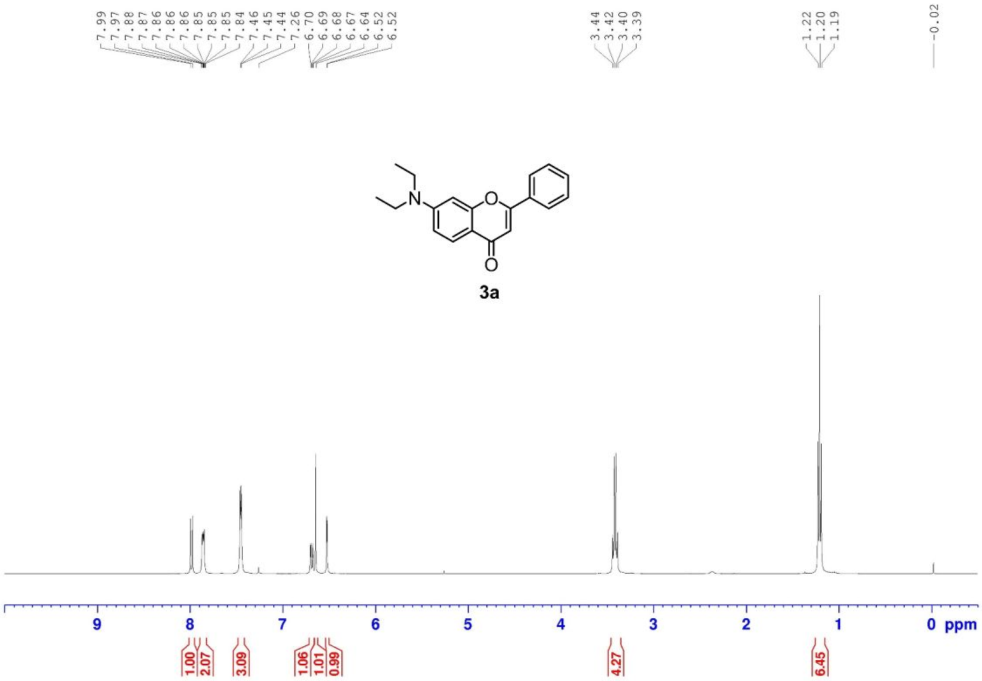

Figure S17. ^{1}H NMR of **3a** in $CDCl_3$

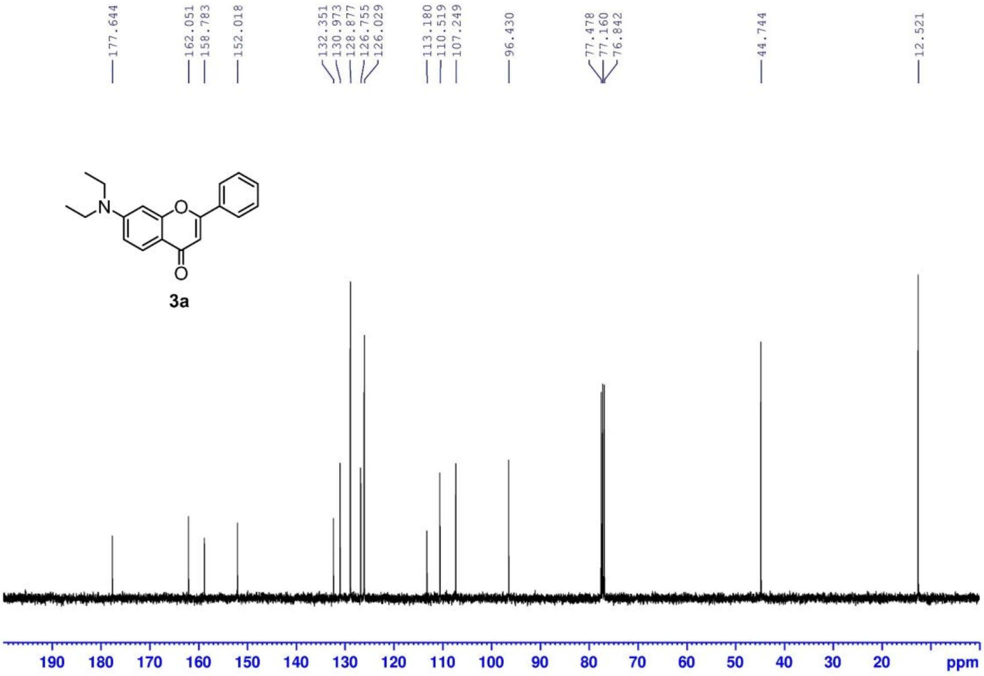

Figure S18. ^{13}C NMR of **3a** in $CDCl_3$

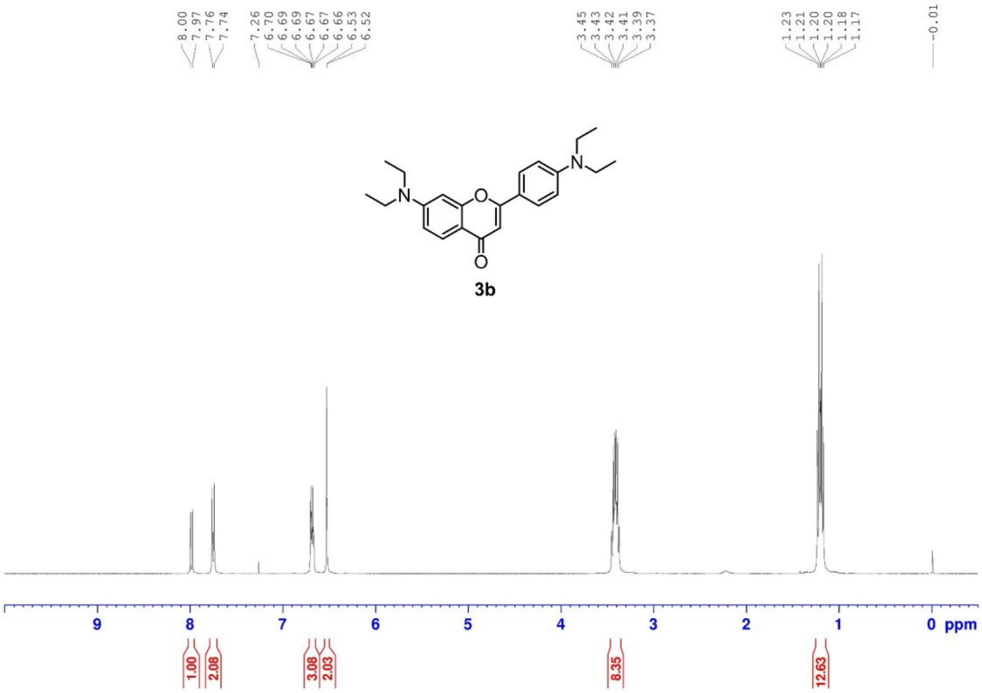

Figure S19. ^{1}H NMR of **3b** in $CDCl_3$

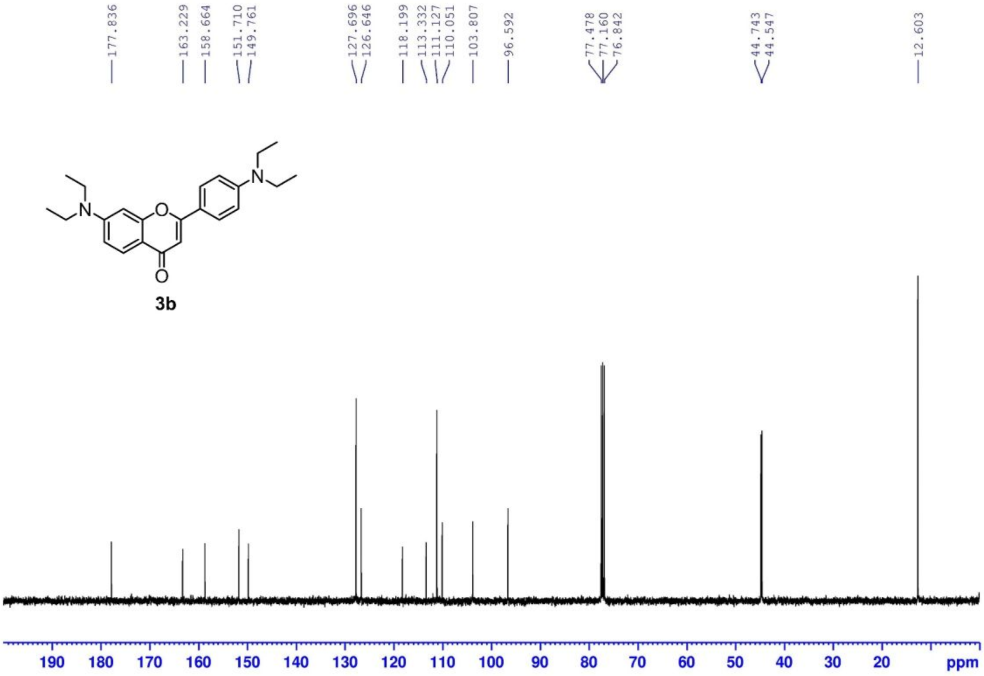

Figure S20. ^{13}C NMR of **3b** in $CDCl_3$

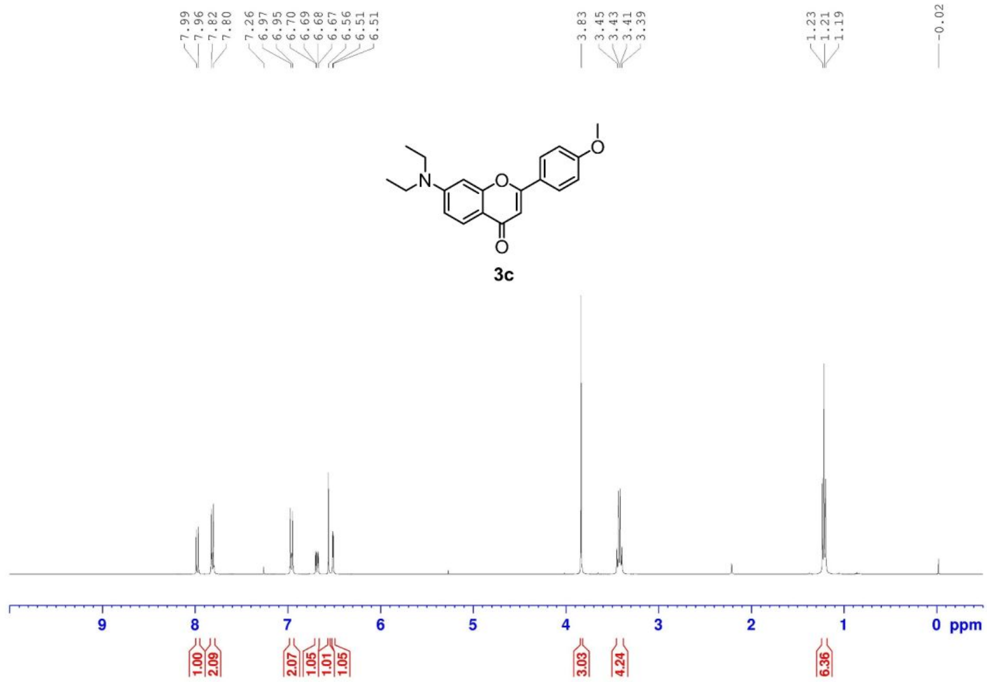

Figure S21. ^{1}H NMR of **3c** in $CDCl_3$

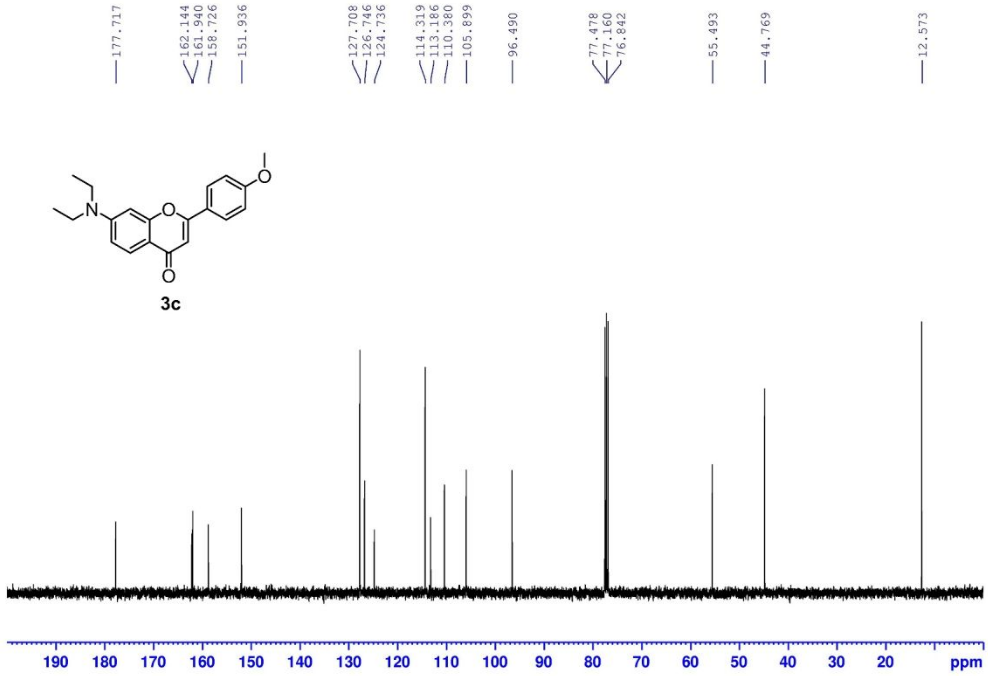

Figure S22. ^{13}C NMR of **3c** in $CDCl_3$

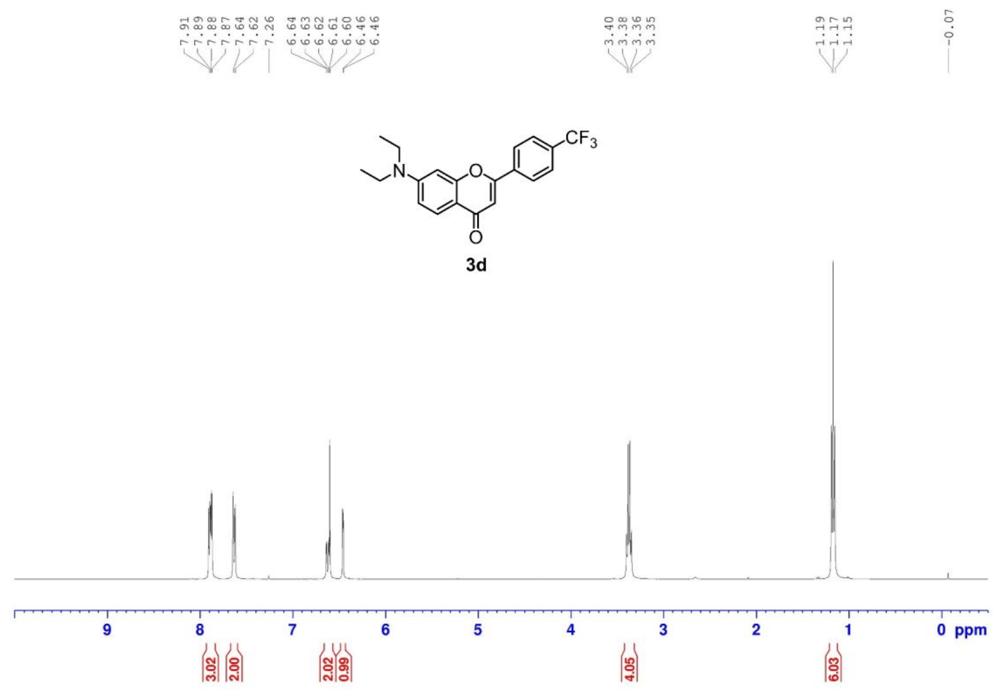

Figure S23. ^{1}H NMR of **3d** in $CDCl_3$

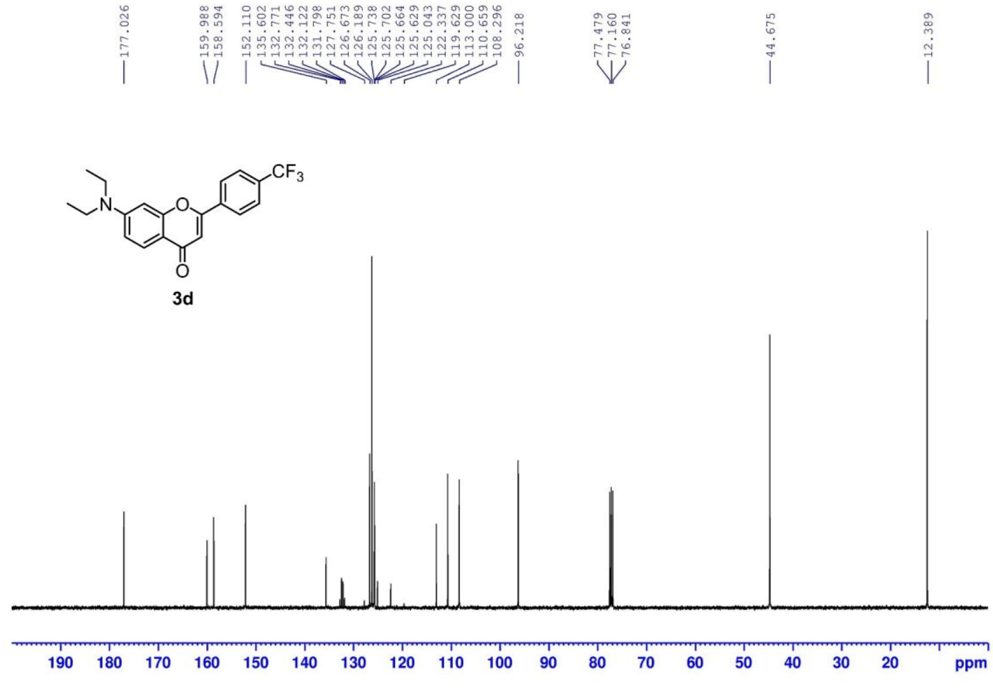

Figure S24. ^{13}C NMR of **3d** in $CDCl_3$

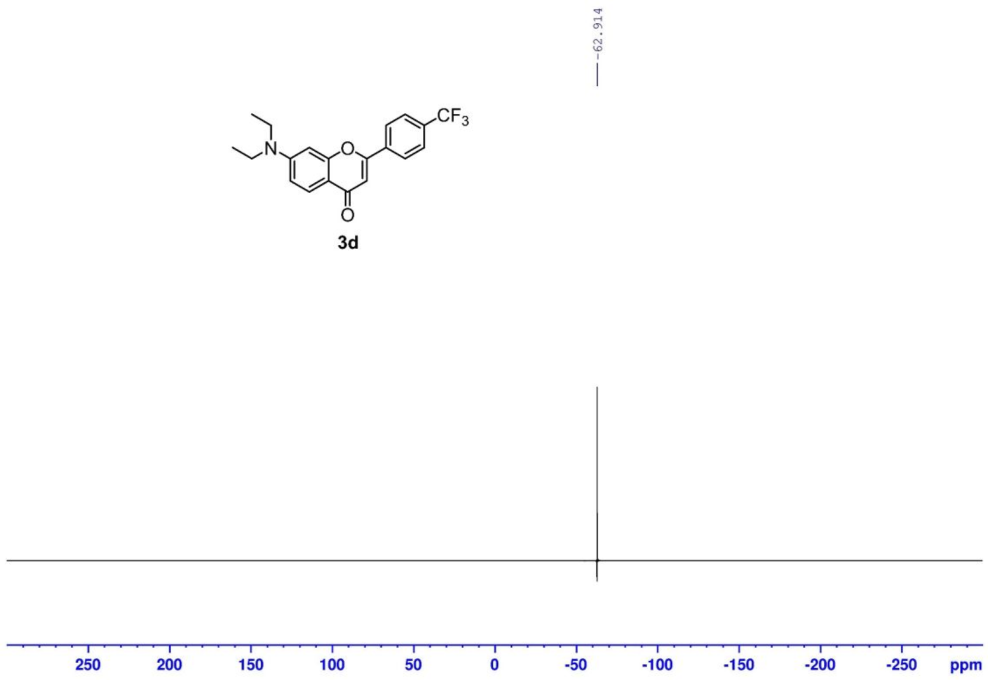

Figure S25. ^{19}F NMR of **3d** in $CDCl_3$

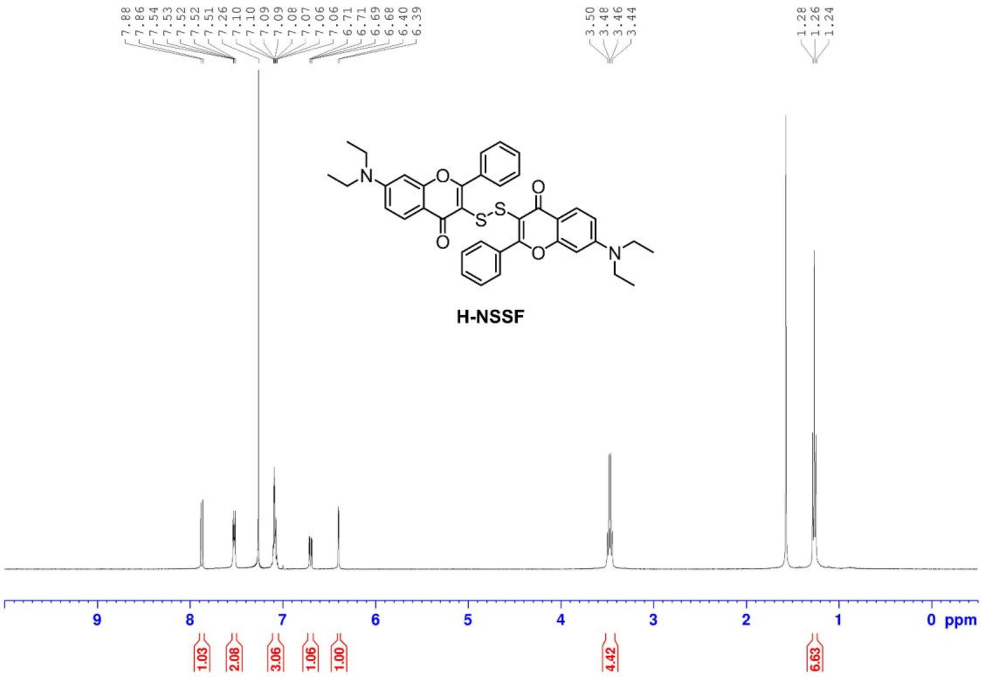

Figure S26. ^{1}H NMR of **H-NSSF** in $CDCl_3$

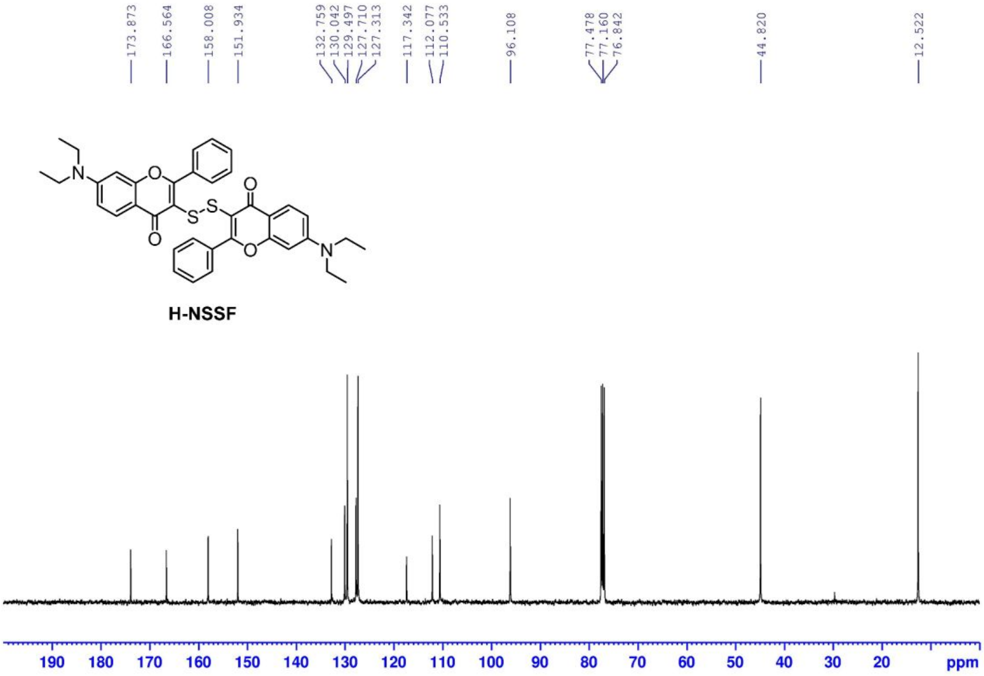

Figure S27. ^{13}C NMR of **H-NSSF** in $CDCl_3$

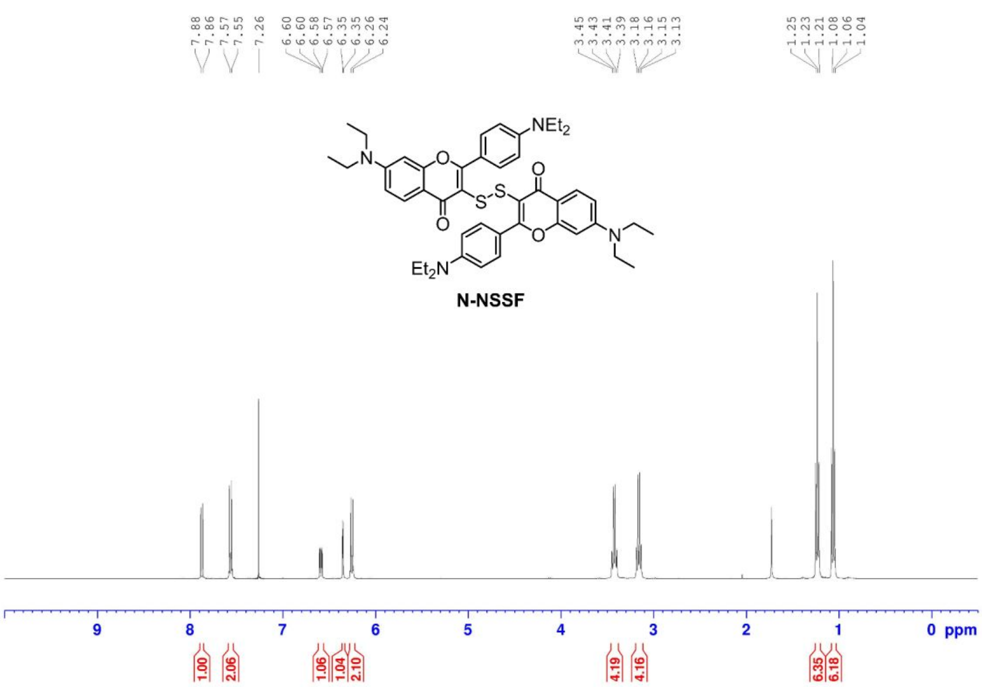

Figure S28. ^{1}H NMR of **N-NSSF** in $CDCl_3$

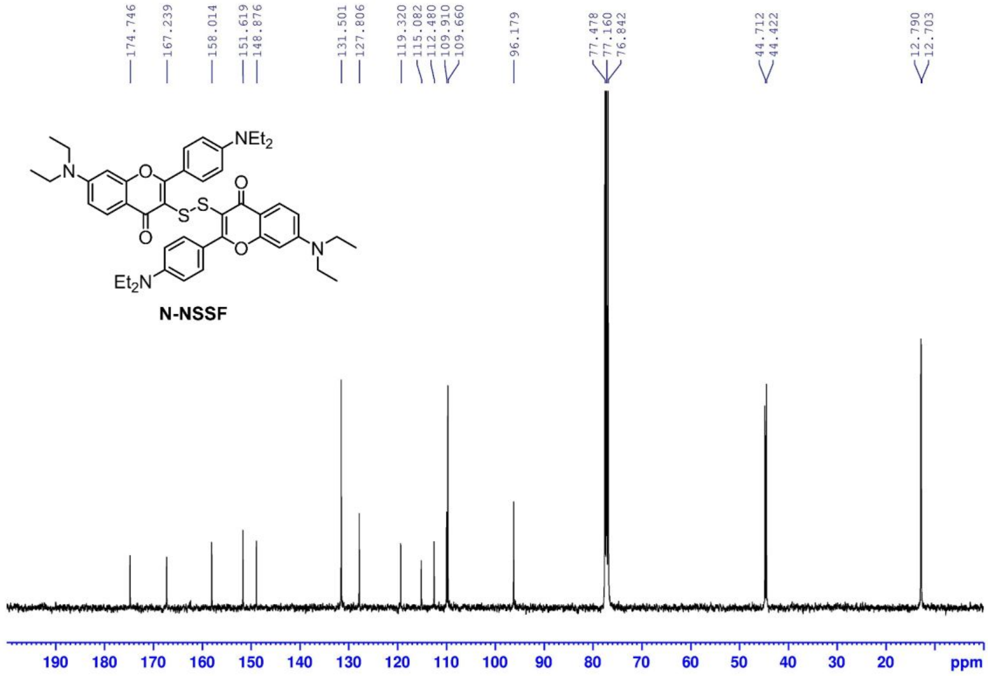

Figure S29. ^{13}C NMR of **N-NSSF** in $CDCl_3$

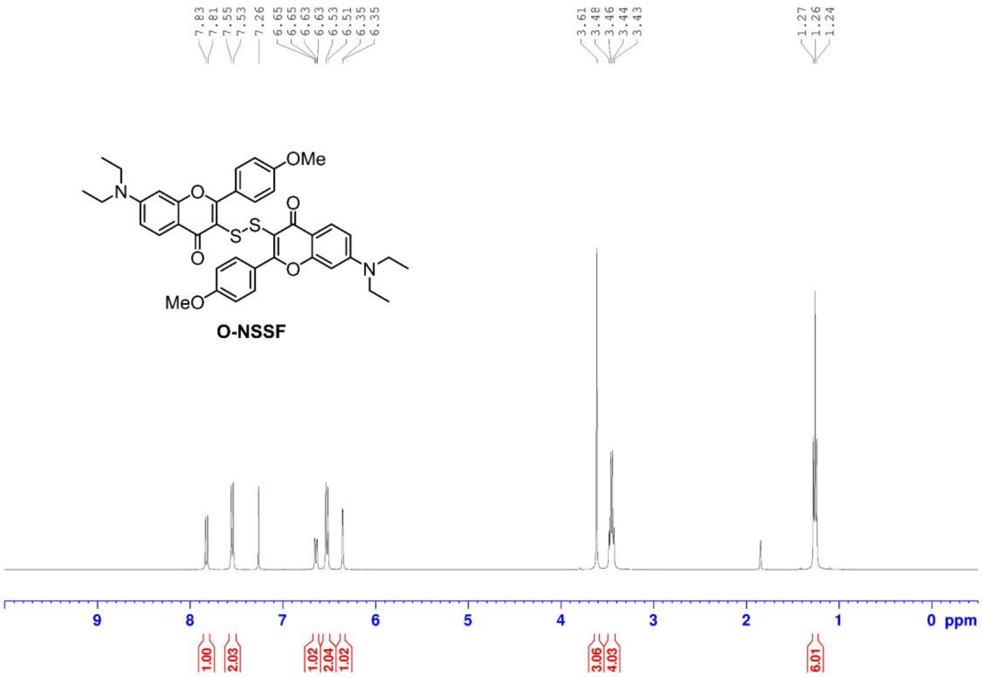

Figure S30. ^{1}H NMR of **O-NSSF** in $CDCl_3$

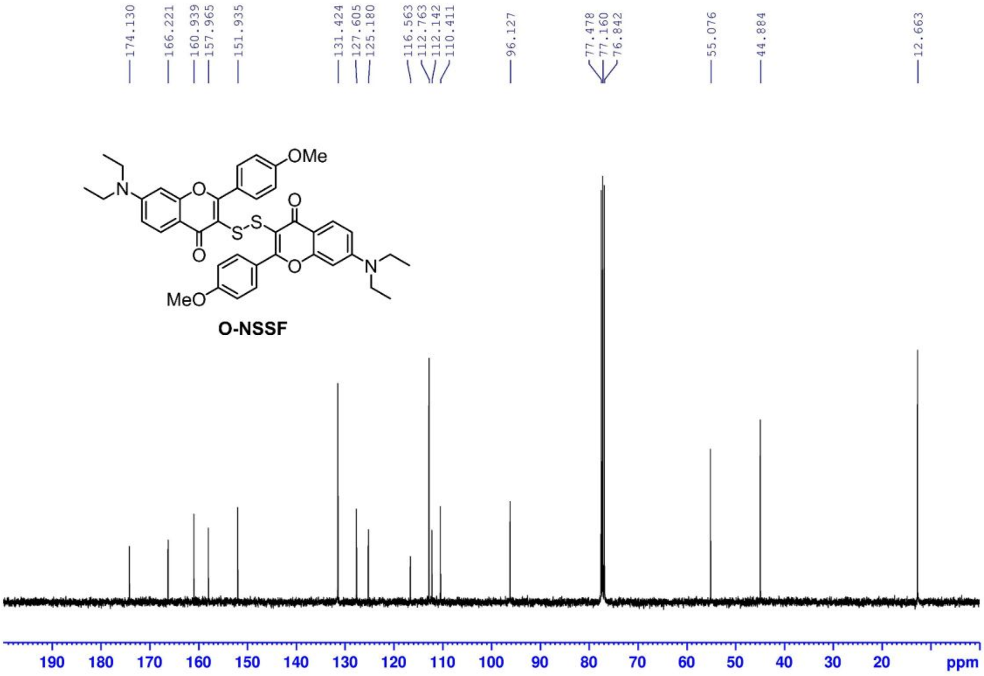

Figure S31. ^{13}C NMR of **O-NSSF** in $CDCl_3$

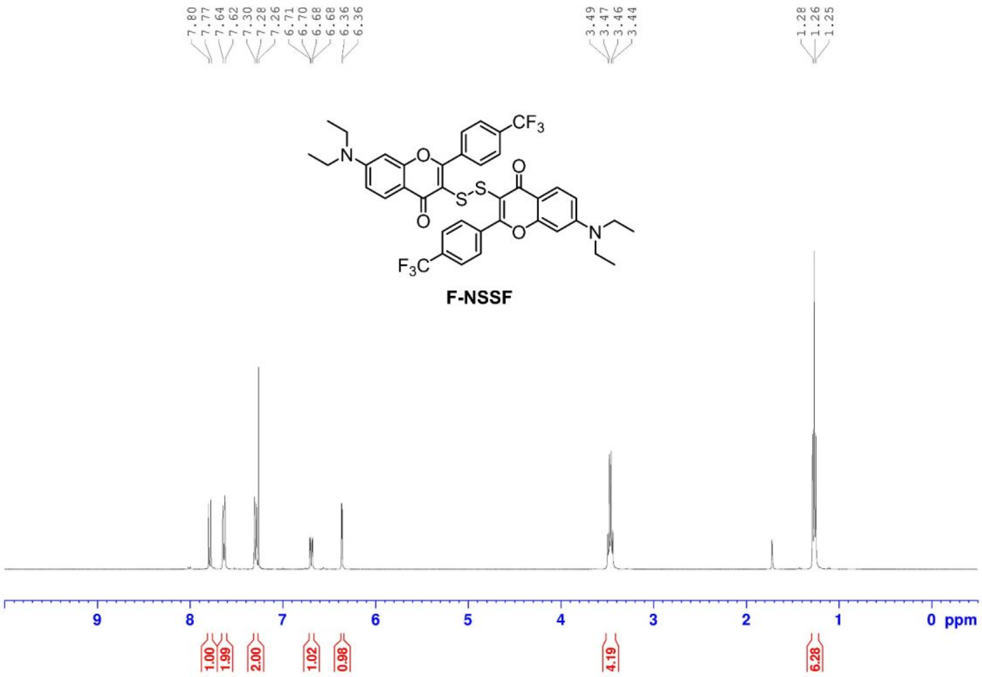

Figure S32. ^{1}H NMR of **F-NSSF** in $CDCl_3$

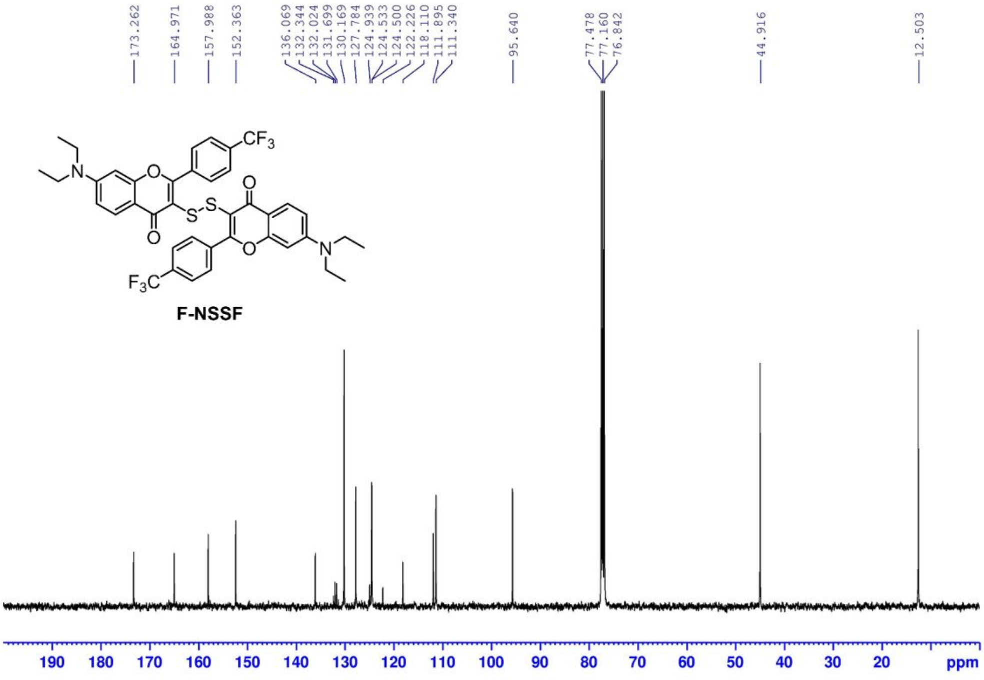

Figure S33. ^{13}C NMR of **F-NSSF** in $CDCl_3$

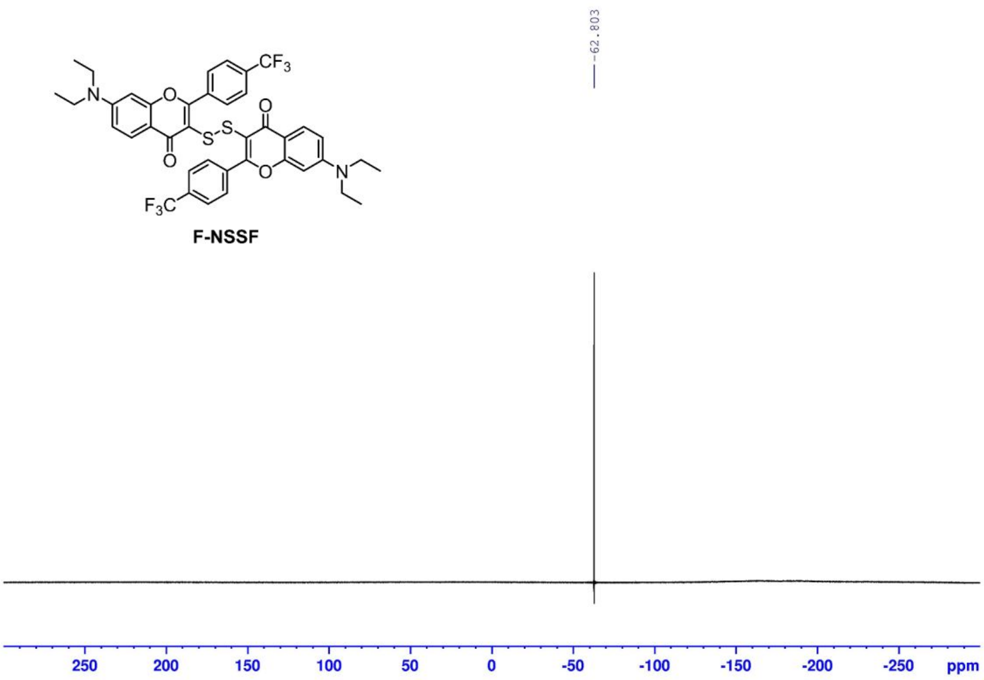

Figure S34. ^{19}F NMR of **F-NSSF** in $CDCl_3$

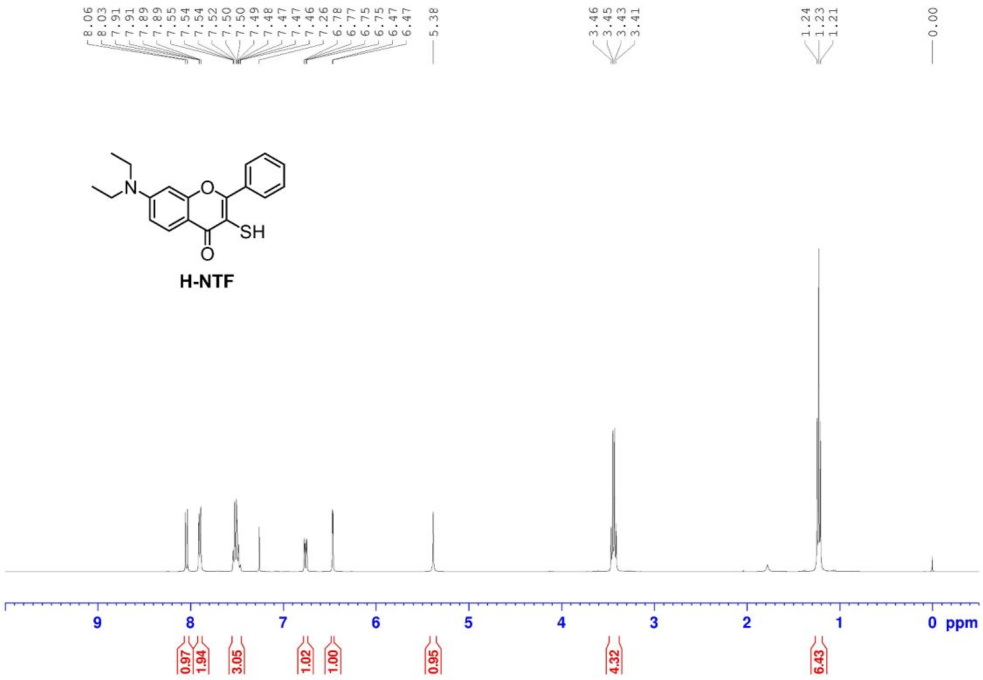

Figure S35. ^{1}H NMR of **H-NTF** in $CDCl_3$

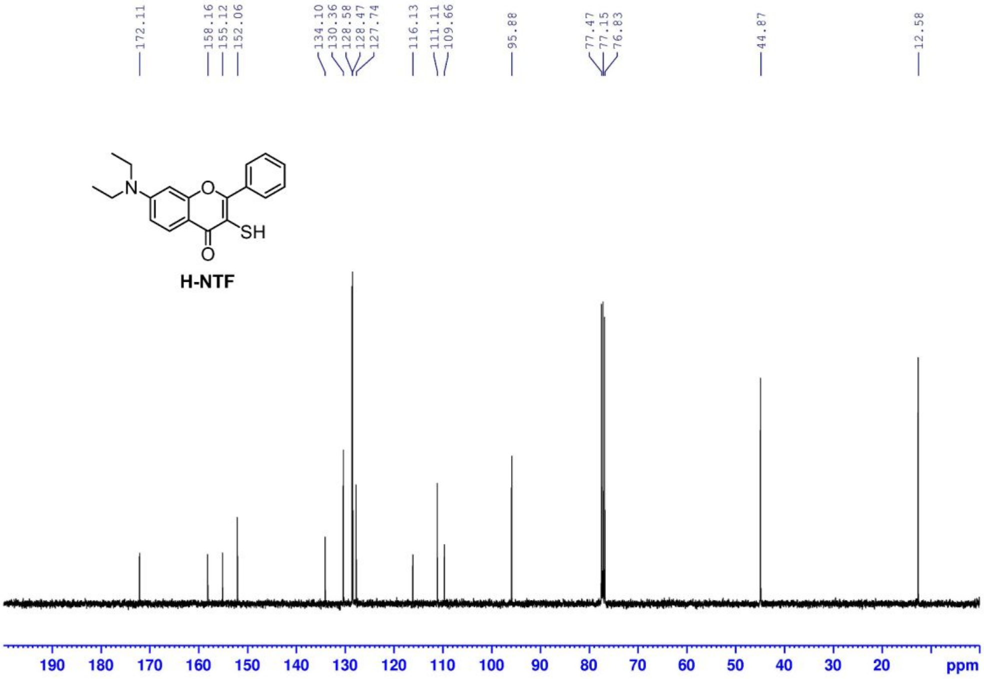

Figure S36. ^{13}C NMR of **H-NTF** in $CDCl_3$

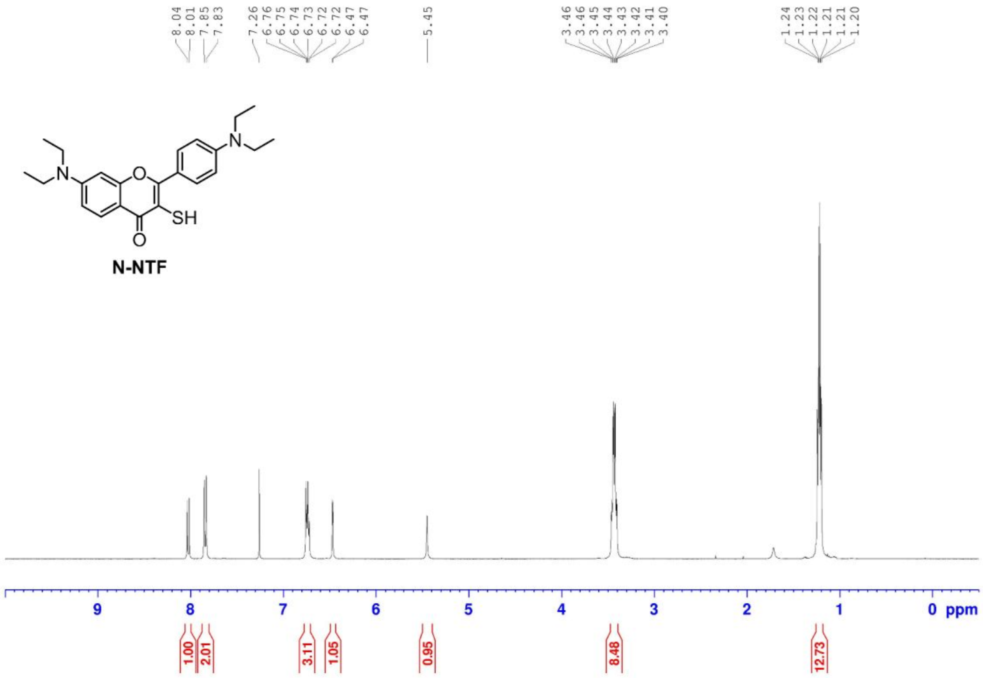

Figure S37. ¹H NMR of **N-NTF** in $CDCl_3$

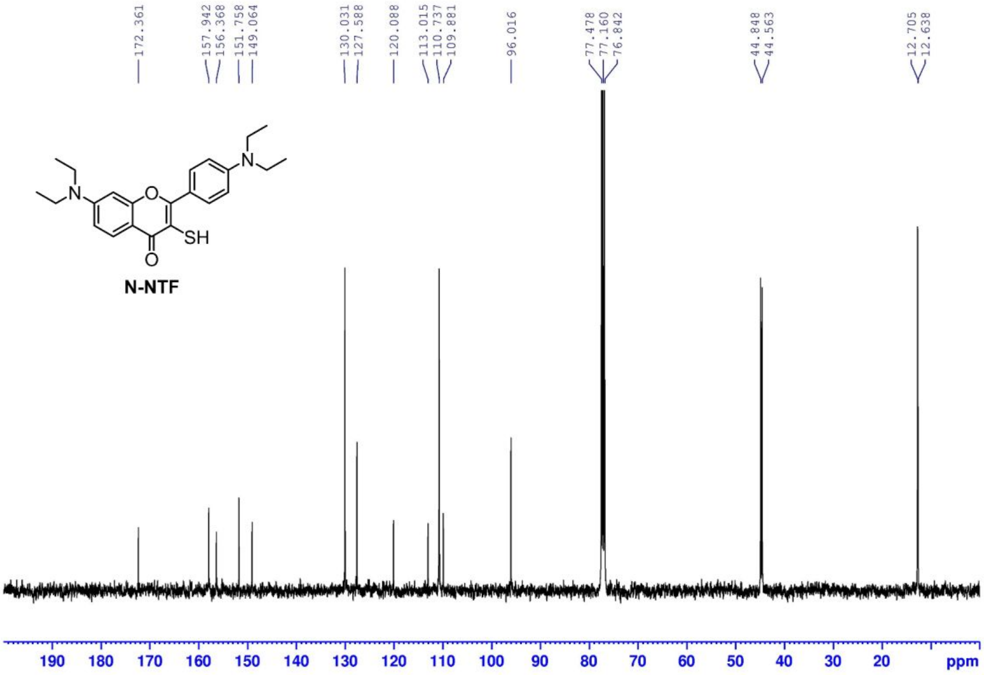

Figure S38. ¹³C NMR of **N-NTF** in $CDCl_3$

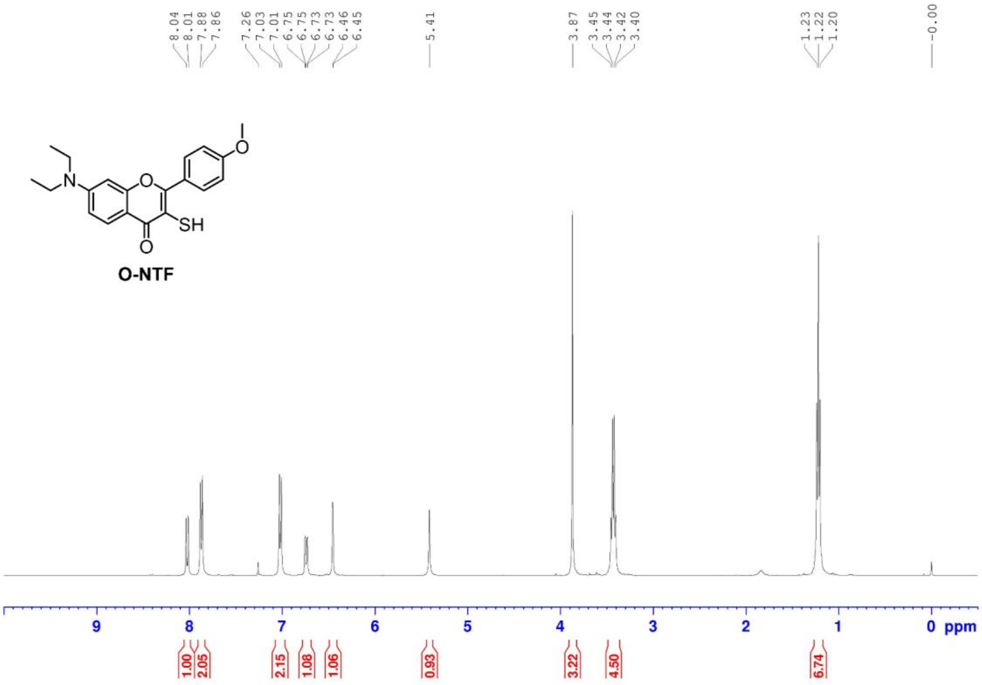

Figure S39. ^{1}H NMR of **O-NTF** in $CDCl_3$

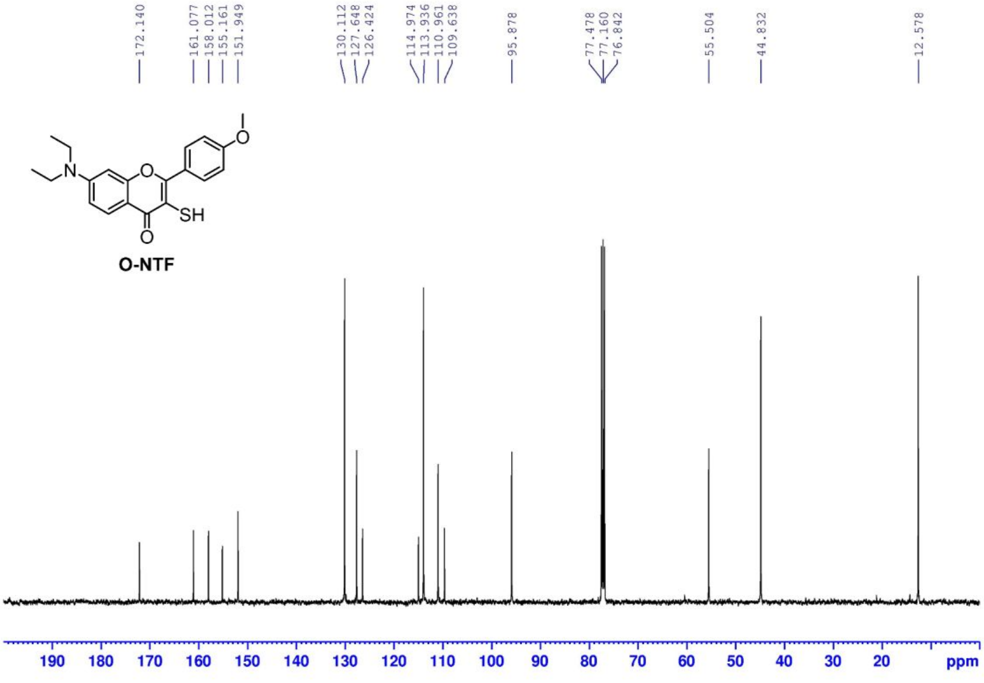

Figure S40. ^{13}C NMR of **O-NTF** in $CDCl_3$

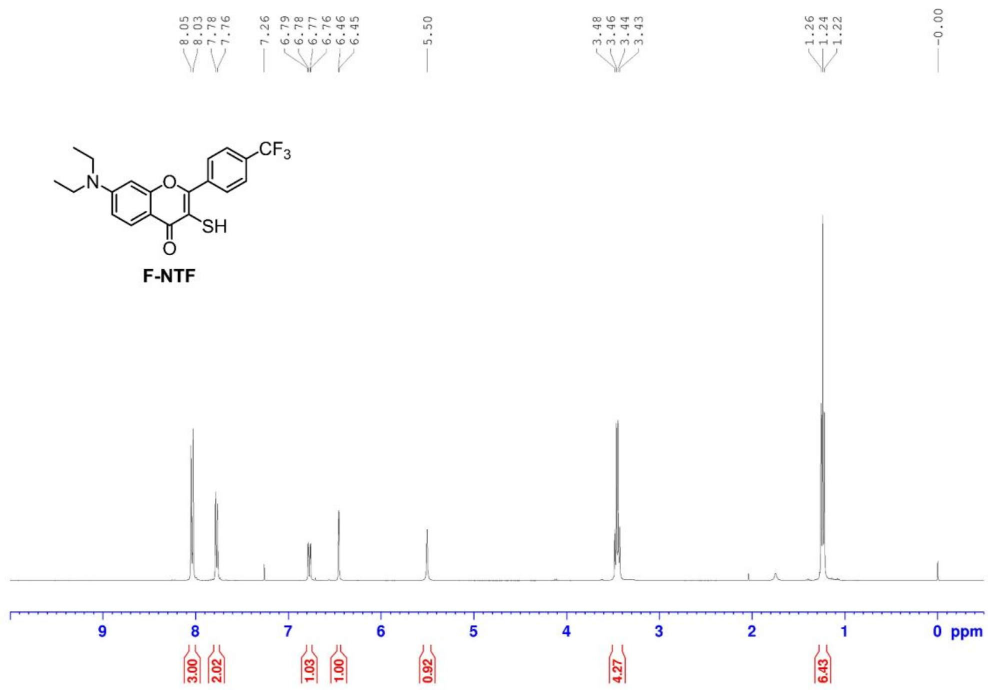

Figure S41. ^{1}H NMR of **F-NTF** in $CDCl_3$

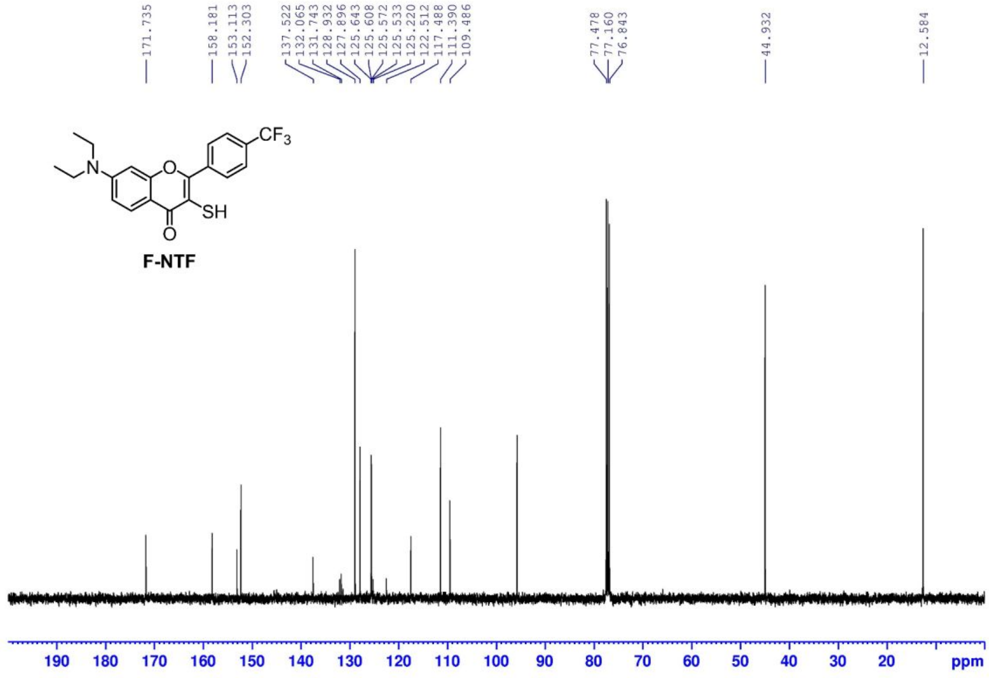

Figure S42. ^{13}C NMR of **F-NTF** in $CDCl_3$

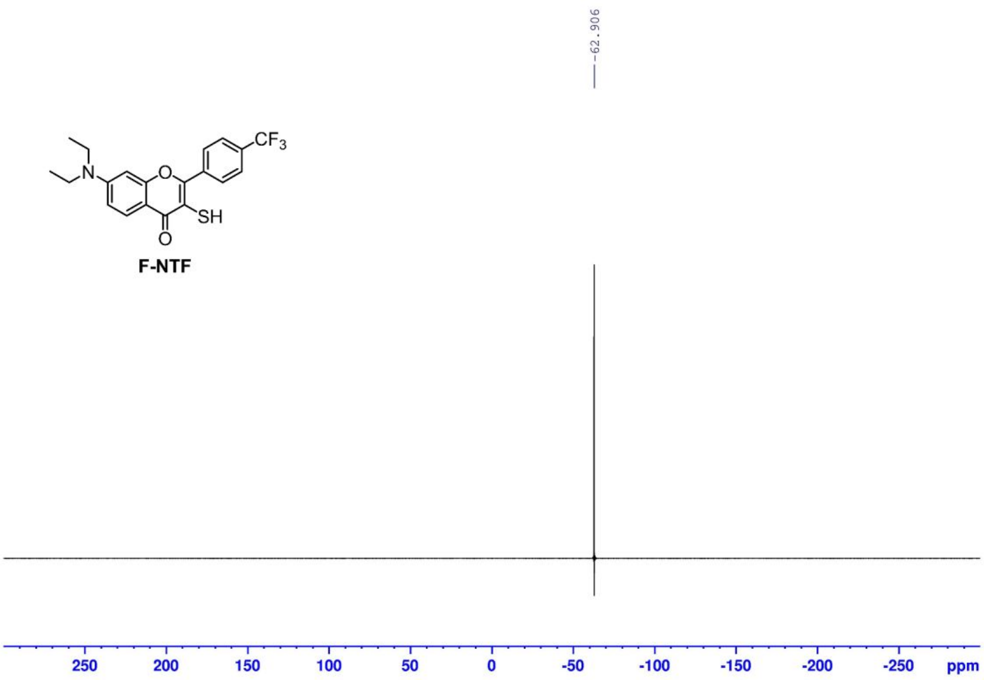

Figure S43. ^{19}F NMR of **F-NTF** in $CDCl_3$

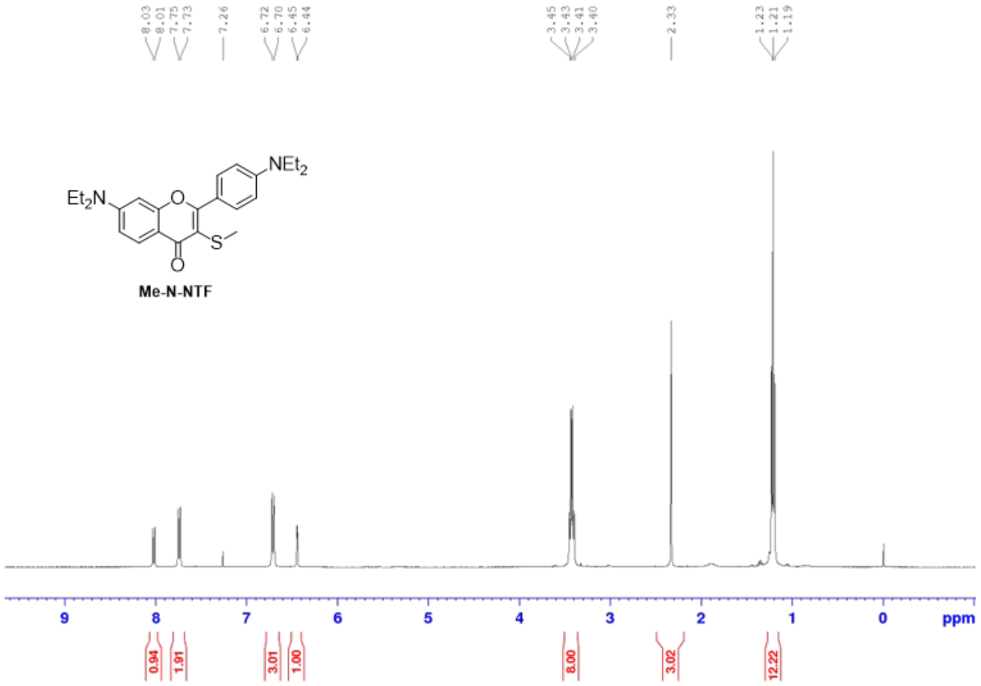

Figure S44. ^{1}H NMR of **Me-N-NTF** in $CDCl_3$

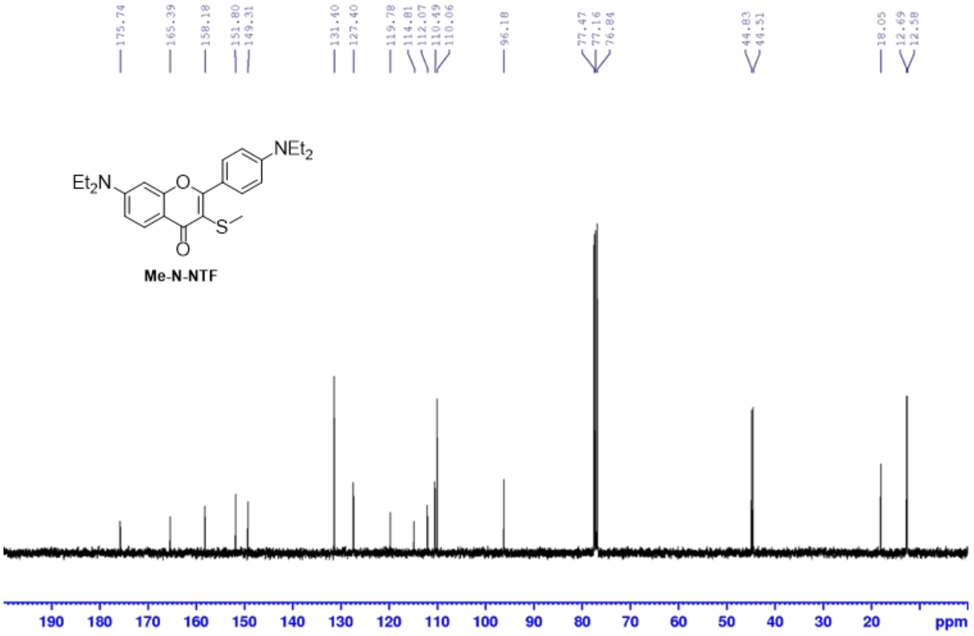

Figure S45. ^{13}C NMR of **Me-N-NTF** in $CDCl_3$

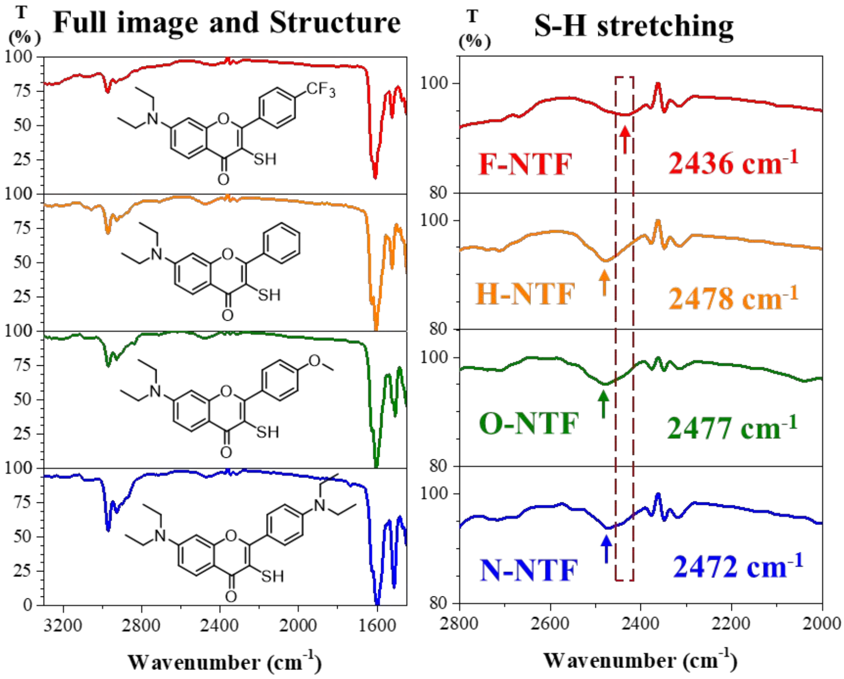

Figure S46. S-H stretching wavenumber obtained via FTIR (KBr). The SH stretching peak wavenumber of **F-NTF**, **H-NTF**, **O-NTF**, and **N-NTF** are depicted in red, orange, green, and blue respectively.

Table S1. Single-crystal XRD data for **F-NTF** (CCDC 2295378)

**Crystal data**		
Empirical formula	C20 H18 F3 N O2 S	
Formula weight	393.41	
Crystal system	Triclinic	
Space group	P-1	
Unit cell dimensions	a = 4.7673(4) Å	a= 88.822(3)°.
	b = 10.4607(9) Å	b= 85.630(3)°.
	c = 17.9972(13) Å	g = 77.540(3)°.
Volume	873.81(12) $Å^3$	
Z	2	
F(000)	408	
Density (calculated)	1.495 Mg/m^3	
Wavelength	0.71073 Å	
Cell parameters reflections used	9828	
Theta range for Cell parameters	3.02 to 27.49°.	
Absorption coefficient	0.232 mm^{-1}	
Temperature	100(2) K	
Crystal size	0.200 x 0.100 x 0.025 mm^3	
**Data collection**		
Diffractometer	Bruker AXS D8 VENTURE,	
PhotonIII_C28		
Absorption correction	Semi-empirical from equivalents	
Max. and min. transmission	1.0000 and 0.8723	
No. of measured reflections	23776	
No. of independent reflections	4014 [R(int) = 0.0675]	
No. of observed [I>2_igma(I)]	3354	
Completeness to theta = 25.242°	99.9 %	
Theta range for data collection	1.994 to 27.514°.	
**Refinement**		
Final R indices [I>2sigma(I)]	R1 = 0.0445, wR2 = 0.1132	
R indices (all data)	R1 = 0.0535, wR2 = 0.1210	
Goodness-of-fit on F^2	1.018	
No. of reflections	4014	
No. of parameters	278	
No. of restraints	36	
Largest diff. peak and hole	0.290 and -0.340 e.$Å^{-3}$	

Table S2. Single-crystal XRD data for **H-NTF** (CCDC 2295381)

**Crystal data**		
Empirical formula	C19 H19 N O2 S	
Formula weight	325.41	
Crystal system	Monoclinic	
Space group	$P2_1/c$	
Unit cell dimensions	a = 8.1415(3) Å	a= 90°.
	b = 14.4182(6) Å	b= 102.6978(15)°.
	c = 14.0301(6) Å	g = 90°.
Volume	1606.65(11) $Å^3$	
Z	4	
F(000)	688	
Density (calculated)	1.345 Mg/m^3	
Wavelength	0.71073 Å	
Cell parameters reflections used	8477	
Theta range for Cell parameters	2.83 to 28.71°.	
Absorption coefficient	0.211 mm^{-1}	
Temperature	100(2) K	
Crystal size	0.180 x 0.100 x 0.050 mm^3	
**Data collection**		
Diffractometer	Bruker AXS D8 VENTURE,	
PhotonIII_C28		
Absorption correction	Semi-empirical from equivalents	
Max. and min. transmission	1.0000 and 0.9533	
No. of measured reflections	36702	
No. of independent reflections	4163 [R(int) = 0.0522]	
No. of observed [I>2_igma(I)]	3539	
Completeness to theta = 25.242°	100.0 %	
Theta range for data collection	2.052 to 28.767°.	
**Refinement**		
Final R indices [I>2sigma(I)]	R1 = 0.0358, wR2 = 0.0930	
R indices (all data)	R1 = 0.0425, wR2 = 0.0988	
Goodness-of-fit on F^2	1.038	
No. of reflections	4163	
No. of parameters	214	
No. of restraints	0	
Largest diff. peak and hole	0.306 and -0.288 e.$Å^{-3}$	

Table S3. Single-crystal XRD data for **O-NTF** (CCDC 2295379)

**Crystal data**		
Empirical formula	C20 H21 N O3 S	
Formula weight	355.44	
Crystal system	Monoclinic	
Space group	C2/c	
Unit cell dimensions	a = 21.9535(5) Å	a= 90°.
	b = 8.1948(2) Å	b= 102.7986(7)°.
	c = 19.9641(4) Å	g = 90°.
Volume	3502.40(14) $Å^3$	
Z	8	
F(000)	1504	
Density (calculated)	1.348 Mg/m^3	
Wavelength	1.54178 Å	
Cell parameters reflections used	9838	
Theta range for Cell parameters	2.27 to 78.09°.	
Absorption coefficient	1.797 mm^{-1}	
Temperature	100(2) K	
Crystal size	0.150 x 0.150 x 0.100 mm^3	
**Data collection**		
Diffractometer	Bruker AXS D8 VENTURE	
Absorption correction	Semi-empirical from equivalents	
Max. and min. transmission	1.0000 and 0.8626	
No. of measured reflections	32122	
No. of independent reflections	3542 [R(int) = 0.0315]	
No. of observed [I>2_igma(I)]	3414	
Completeness to theta = 67.679°	98.9 %	
Theta range for data collection	4.542 to 78.007°.	
**Refinement**		
Final R indices [I>2sigma(I)]	R1 = 0.0439, wR2 = 0.1210	
R indices (all data)	R1 = 0.0449, wR2 = 0.1222	
Goodness-of-fit on F^2	1.093	
No. of reflections	3542	
No. of parameters	233	
No. of restraints	0	
Largest diff. peak and hole	0.888 and -0.383 e.$Å^{-3}$	

Table S4. Single-crystal XRD data for **N-NTF** (CCDC 2295382)

**Crystal data**		
Empirical formula	C23 H28 N2 O2 S	
Formula weight	396.53	
Crystal system	Monoclinic	
Space group	$P2_1/c$	
Unit cell dimensions	a = 9.0568(4) Å	a= 90°.
	b = 17.7157(9) Å	b= 96.7986(19)°.
	c = 12.6112(6) Å	g = 90°.
Volume	2009.21(17) $Å^3$	
Z	4	
F(000)	848	
Density (calculated)	1.311 Mg/m^3	
Wavelength	0.71073 Å	
Cell parameters reflections used	9936	
Theta range for Cell parameters	2.87 to 28.67°.	
Absorption coefficient	0.183 mm^{-1}	
Temperature	100(2) K	
Crystal size	0.350 x 0.250 x 0.200 mm^3	
**Data collection**		
Diffractometer	Bruker AXS D8 VENTURE,	
PhotonIII_C28		
Absorption correction	Semi-empirical from equivalents	
Max. and min. transmission	1.0000 and 0.8386	
No. of measured reflections	47675	
No. of independent reflections	5191 [R(int) = 0.0632]	
No. of observed [I>2_igma(I)]	4680	
Completeness to theta = 25.242°	100.0 %	
Theta range for data collection	1.992 to 28.727°.	
**Refinement**		
Final R indices [I>2sigma(I)]	R1 = 0.0389, wR2 = 0.1003	
R indices (all data)	R1 = 0.0437, wR2 = 0.1049	
Goodness-of-fit on F^2	1.009	
No. of reflections	5191	
No. of parameters	279	
No. of restraints	18	
Largest diff. peak and hole	0.825 and -0.512 e.$Å^{-3}$	

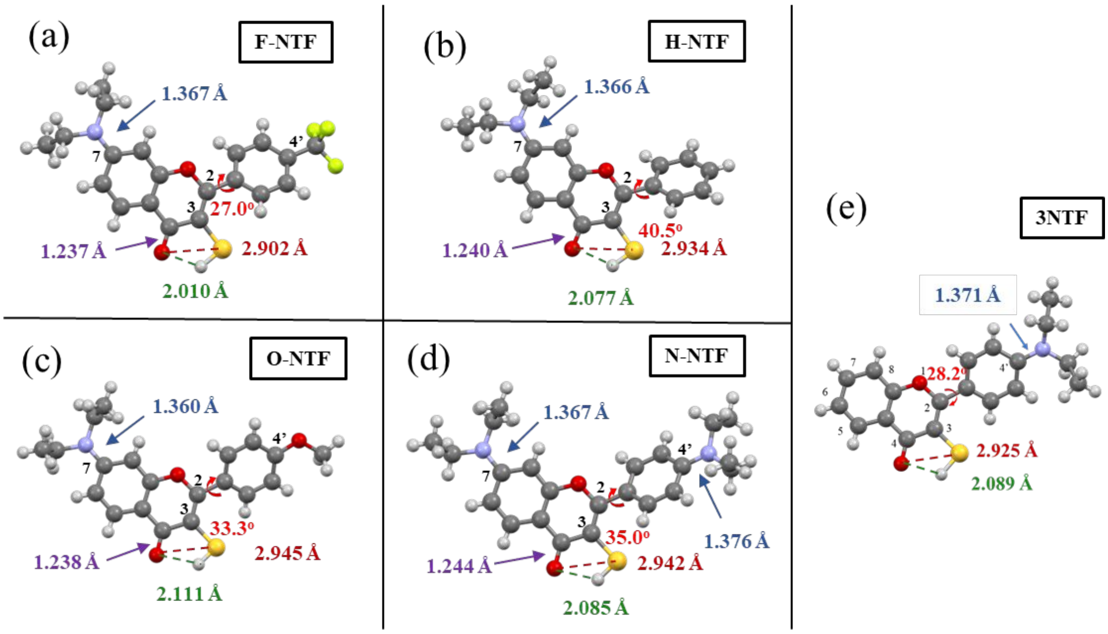

Figure S47. Single-crystal XRD structure of **F-NTF**, **H-NTF**, **O-NTF**, **N-NTF**, and **3NTF**.[1] The displacement ellipsoids are drawn at the 50% probability level, and the H atoms are drawn as spheres of arbitrary radii.

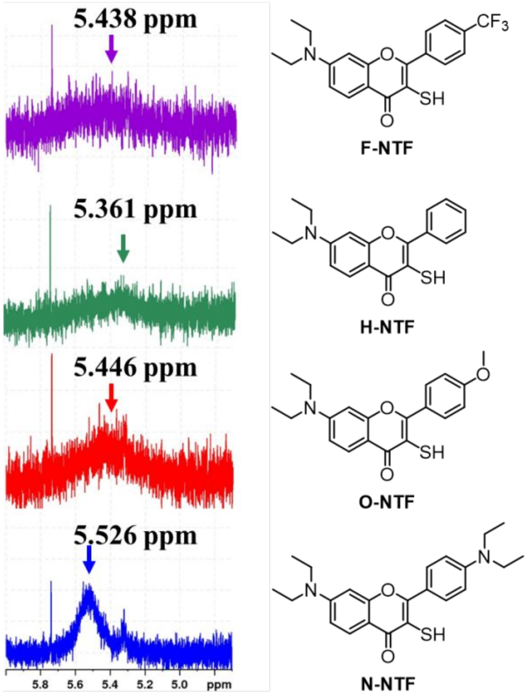

Figure S48. S-H chemical shifts in DMSO-d_6 for **F-NTF**, **H-NTF**, **O-NTF**, and **N-NTF.**

4. Photophysical Properties

4.1 Steady-State Spectra, PLQY and Lifetime Measurements

Steady-state absorption and emission spectra were recorded by a double-beam spectrophotometer (Hitachi U-3310) and a fluorescence spectrometer (Edinburgh FS980), respectively. The photoluminescence quantum yields (PLQYs) were obtained by a comparative method relative to Rhodamine 6G (Q.Y.=0.94) in methanol. The nanosecond time-resolved studies were performed by a time-correlated single photon counting (TCSPC) technique (Edinburgh FLS980) with a picosecond pulsed diode laser as the excitation light source. Both excitation and emission wavelength of FLS 980 were carefully calibrated. The samples were prepared in a 1-cm length cuvette with the absorbance of 0.3 (ca. 2 × 10^{-5} M) at the excitation wavelength.

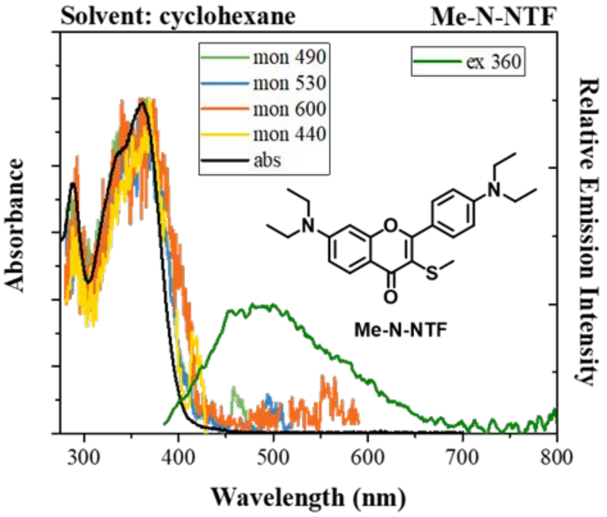

Figure S49. The steady-state absorption, emission, and excitation spectra of **Me-N-NTF** in cyclohexane. The excitation wavelength for the emission is 360 nm.

4.2 Time-resolved fluorescence spectroscopy.

The picosecond time-resolved studies were performed by a TCSPC technique where a ~100 fs laser (400 nm) was used as a pumping source, which incorporated with a microchannel detector gives a time resolution of ~20 ps. As for the fluorescence up-conversion measurement, a stable 35-fs laser system was used as the excitation source. In detail, a 10 fs pulse from Ti:Sapphire-based oscillator (Element 2, Spectra-Physics) seeds a regeneration amplifier (Spitfire ACE, Spectra-Physics). This 10 fs broadband pulse (800 nm, FWHM is 100 nm, 650 mW) was truncated its spectrum of both edges by the stretcher grating, and only reserves the required frequency for a 35 fs pulse (800 nm). The energy of stretched pulse was amplified ~20 cycles in the regen area and then compressed to a 29 fs pulse (800 nm, 5W average power). An autocorrelator,

femtometer (Spectra-Physics), using a *Frequency Resolved Autocorrelation* (FRAC) technique, was applied to measure the pulse duration after the amplifier output.

The 400 nm pump beam was generated by the attenuated amplifier 800 nm output traveling through the second harmonic generation (SHG, β-barium borate crystal cut at theta of 38 grads, Type I). An iris was then installed to select the energy and the beam size after SHG. Three off-axis parabolic mirrors were used to focus pump pulse (1~2mW) on the sample, and the fluorescence was collected and focused on a 0.1 (or 0.2 mm) SHG BBO crystal that was blazed at theta of 38 grads (type I or II). The sample solution was measured in a rotated cell with a transmitted collection mode.

As for the gating, the 800 nm pulse output from the attenuated amplifier is attenuated to 22 mW with the beam size controlled by an iris. The pulse then enters a delay line and acts as a gate pulse crossing the fluorescence beam on the sum-frequency BBO with a collinear alignment. The polarized angle between pump and intercepted fluorescence is set at a magic angle of 54.7˚.

A dual monochromator was applied to decrease the special chirp, which is coupled with an analog photomultiplier to record the up-converted signal. In this study, the slit of the monochromator, the beam size of pump and gate pulses, and the non- or collinear mode determine the FWHM of Instrument Responds Function (IRF). The FWHM of IRF is measured to be 75 and 95 fs, which are in the collinear and non-collinear modes, respectively.

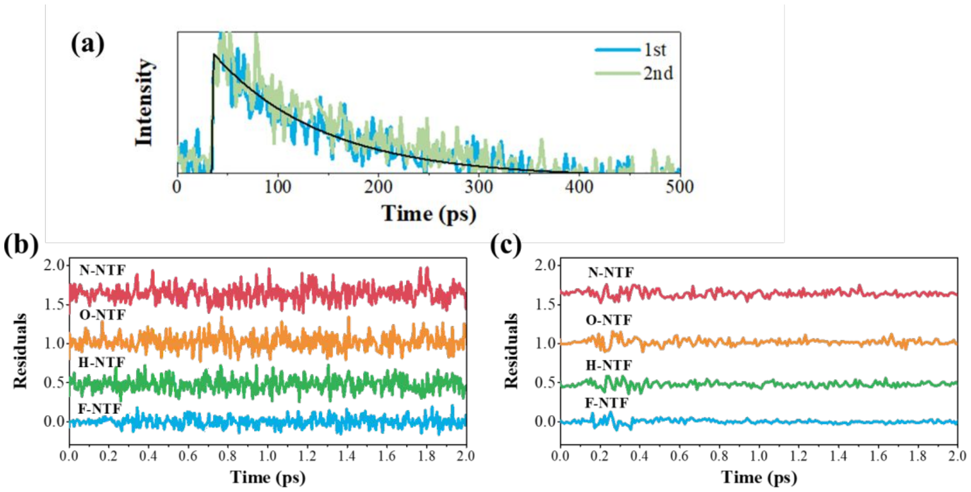

Figure S50. (a) The decay curves were obtained from repeated scans of **F-NTF** in toluene. This result suggests that photodegradation during the fs-upconversion measurement period is negligible. (excitation wavelength: 400 nm).
(b) and (c) represent the residuals of the fitting line for **NTFs** corresponding to the dynamic data in Figures 4-(b) (tautomer rise) and 4-(c) (normal species decay), respectively.

4.3 Impurity issue

During the experiment, we found that it is hard to avoid the species that emit around 540 nm (denoted as F_{short} in the following content) with an excitation spectrum of 430 nm peak. Note that the measurement was carried out under a very low concentration of $10^{-4} \sim 10^{-5}$ M; therefore, the possibility of the origin from the aggregation effect has been eliminated. We assumed that it was an impurity at first, so we conducted a series of experiments trying to find out what compound the species is. The first culprit is thought to be the S-S linked dimer of the 3-mercaptoflavones produced from either the incomplete reduction of the precursor or the oxidation of the titled 3-mercaptflavones. In addition, the absorption of the studied 3-mercaptoflavones with S-S linkage, i.e., the oxidized precursor during the synthesis, renders an absorption band < 400 nm (see Figure S51) that is much different from the observed 400~470 nm excitation bands. Therefore, the origin of the S-S linked dimer can be eliminated. On the other hand, using **F-NTF** as an example, we noticed that photodecomposition took place under an aerated solution (see Figure S52), yielding a trace product that exhibits emission in a similar region as F_{short}. Therefore, as reported in 3-hydroxyflavones,[2] 3-mercaptoflavones may undergo photooxygenation, yielding the product to account for the increase of the emission. However, examination of Figure S52 indicates a much different product emission profile from that of the initial F_{short} band. In addition, upon working up the sample purification under oxygen-free conditions, the 530 nm F_{short} emission was still observed promptly. Therefore, the origin of the F_{short} emission from the photoproduct is discarded.

After eliminating the above possibilities, our source points to a trace of S-H deprotonated NTFs anion (i.e., ion pair in aprotic solvent) emission.[1] One piece of evidence, though rather qualitative, is the disappearance of the 530 nm F_{short} band upon adding a tiny fuming HCl gas to the cyclohexane solution, implying the anion, implying purposed anion is re-protonated. This tentative proposal seems to be reasonable because the thiol acidity of NTFs is in the order of **N-NTF** < **O-NTF** < **H-NTF** < **F-NTF**, which qualitatively correlates with the increase of the F_{short} emission intensity (see Figures S53 left part and S54). Nevertheless, we must be honest that this explanation may not be completely convincing because **NTFs**, especially **F-NTF**, still have different F_{short}/F_{tau} ratios in different batches of synthesis and purification.

We then attempted to perform sublimation as an additional purification. To our delight, the emission of F_{short} in all **NTFs** is either negligible or rather small (see Figure S53). The result represents a significant improvement in purification, as well as indicates that the emission of F_{short} is more plausibly from an impurity rather than the claimed ion pair existing intrinsically.

In yet another approach, we also tried hard to investigate impurities. Apparently, this

impurity is present in trace amounts but has a significantly higher emission quantum yield (QY) compared to the main ESIPT species, whose tautomer has a lower emission QY. This makes structure determination of impurities extremely challenging. From a chemical perspective, impurities may arise from side reactions with oxygen during synthesis or purification, such as SO_n-related derivatives.[3] However, this is only speculation, and its structure remains unresolved.

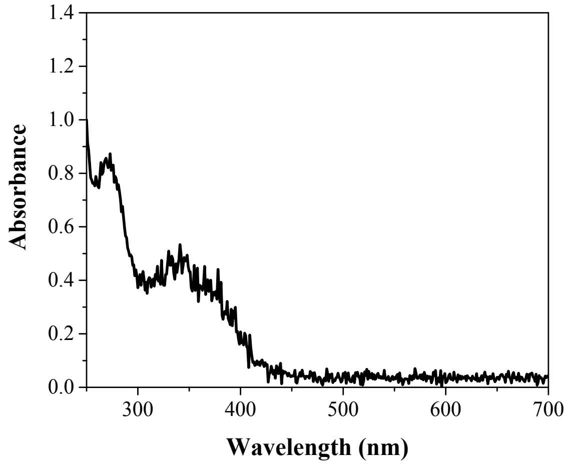

Figure S51. The absorption spectrum of **N-NSSF** (i.e., **N-NTF** dimer with an S-S linkage) in cyclohexane.

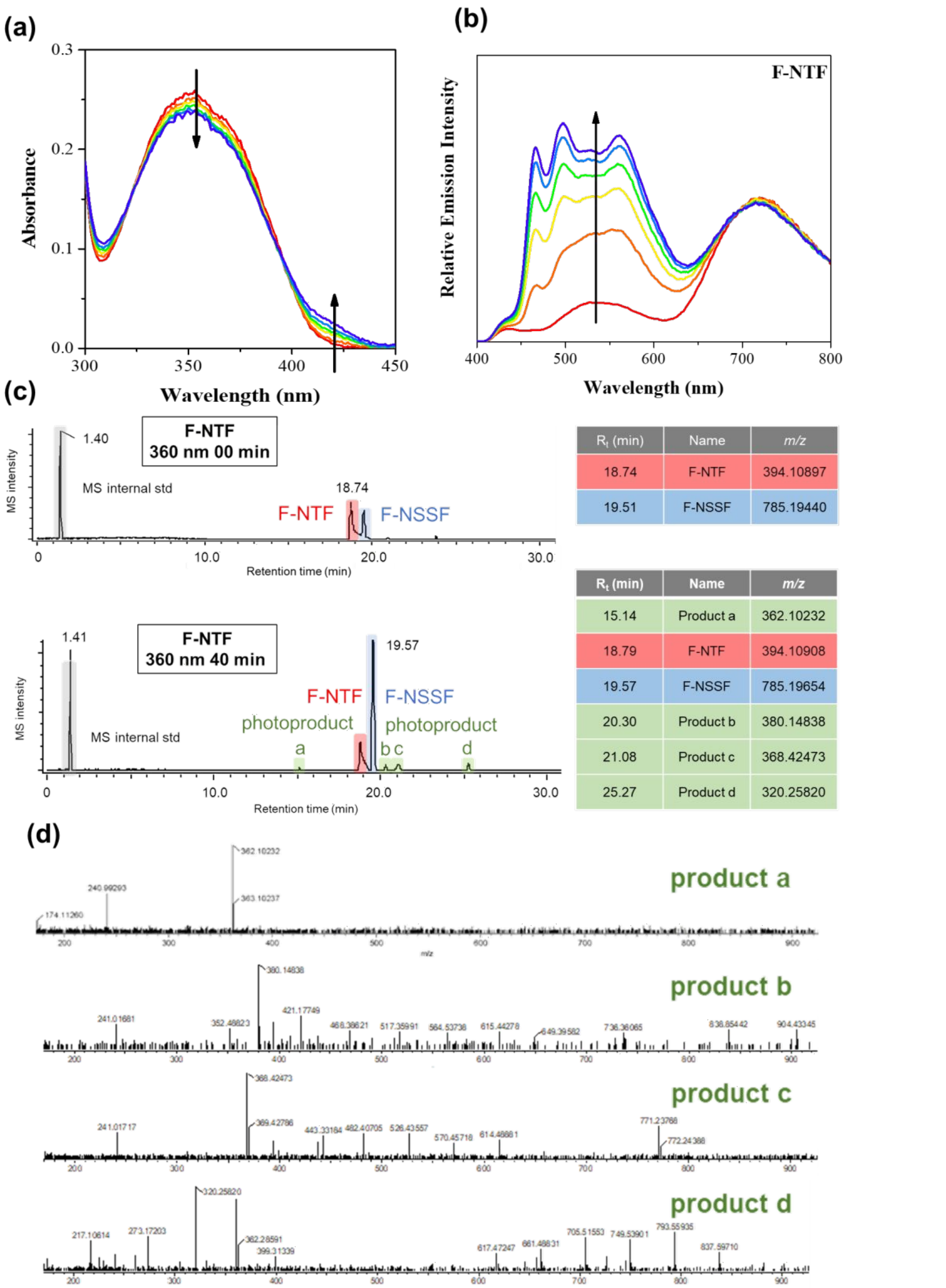

R_t (min)	Name	m/z
18.74	F-NTF	394.10897
19.51	F-NSSF	785.19440

R_t (min)	Name	m/z
15.14	Product a	362.10232
18.79	F-NTF	394.10908
19.57	F-NSSF	785.19654
20.30	Product b	380.14838
21.08	Product c	368.42473
25.27	Product d	320.25820

Figure S52. The absorption (a) and emission (b) spectrum of **F-NTF** in cyclohexane. The emission spectrum was excited at 360 nm. The F-NTF solution was exposed by 360 nm UV light (~70 μW) and measured with an interval of every 20 mins. Furthermore, the crude of this reaction was also analyzed with LC-ESI-MS and the trace and mass spectrum of photoproduct are shown in (c) and (d) respectively. Absorption, emission spectrum and LCMS trace indicate the low photodecomposition rate, with the resulting complex high PLQY products exhibiting pronounced fluorescence.

LC-ESI-MS method:

The analysis was conducted with a JEOL AccuTOF mass spectrometer coupled with an Agilent 1100 HPLC system and a Waters Xbridge column (C18 5 μm 4.6 x 250 mm). Mobile phase: MeCN: H2O (containing 0.1% formic acid) = 70: 30 linearly to 100: 0 within 20 minutes followed by 10 min of pure MeCN. The flow rate is 0.1 mL/min for mass spectrometer.

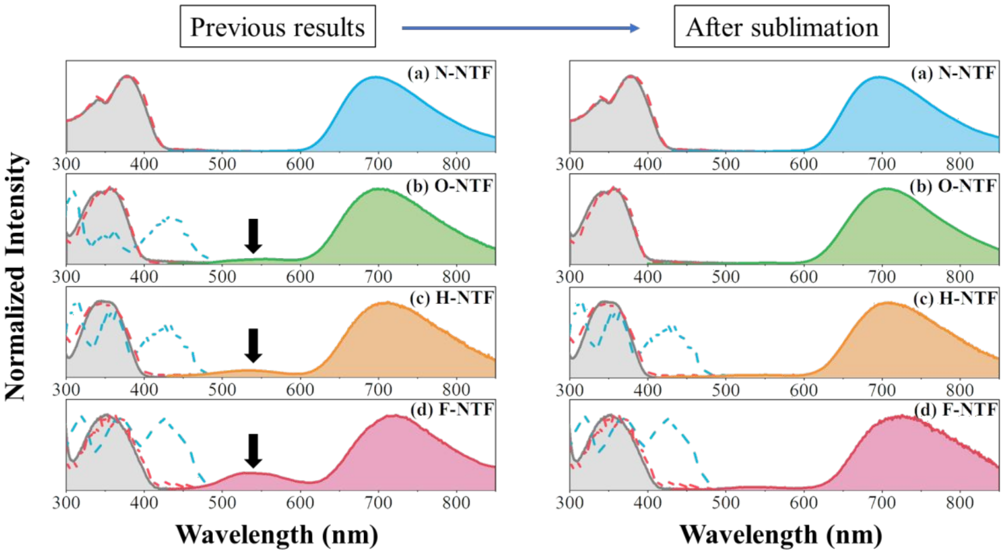

Figure S53. The compared steady-state spectrum of **NTFs** before (left) and after (right) additional sublimation purified.

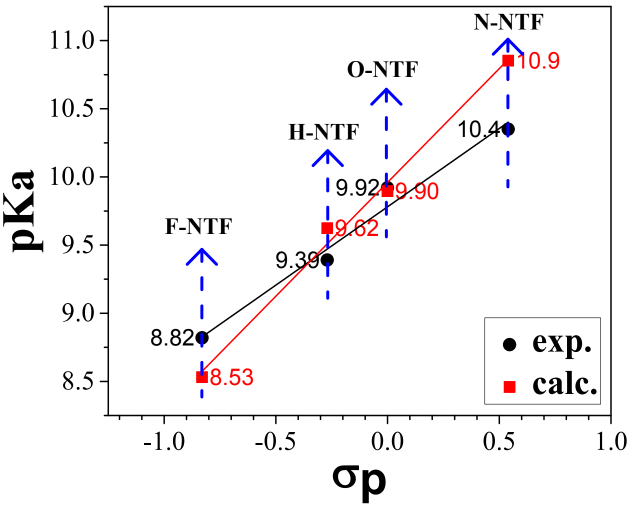

Figure S54. The pKa value of each compound is achieved by the experiment and calculation respectively.

Spectrophotometric titrations were performed in ethanol/water solutions (1:1 v/v) across a range of pH levels. To initiate the experiments, the initial solution underwent acidification using 1 M HCl, resulting in a pH of 3. Subsequently, incremental additions of 1 M NaOH were made, elevating the pH to 13. Stock solutions of the investigated compounds in ethanol were prepared meticulously at a concentration of 10^{-3} M. For each compound, 10 μL of the stock solution was introduced into 2 mL of the ethanol/water solution at varying pH levels. The conversion of the measured pKa value in the mixed solvent to its corresponding value in water necessitates consideration of the disparity in solvent properties. A more straightforward approach involves employing the concept of solvent dielectric constant.[4-5] This approximation method assumes that the solvent's influence is solely governed by the dielectric constant, and to further validate the results of the experimental measurements, computational methods[6-8] were utilized (red data, vide infra).

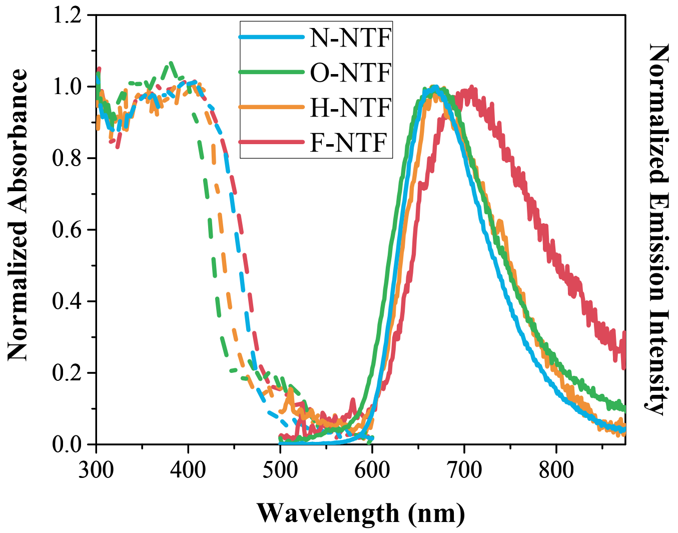

Figure S55. The emission and absorption spectra of the studied **NTFs** in solid powder. The absorption spectra of solid powders were measured by an integrating sphere. The emission spectra were measured by 390 nm excitation. Note that all **NTFs** exhibit solely the proton-transfer tautomer emission.

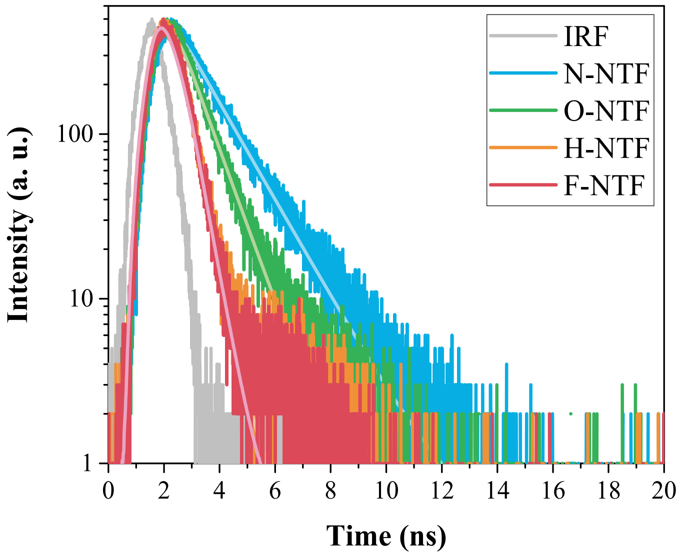

Figure S56. The tautomer emission lifetime of solid powder **NTFs** was measured using the TCSPC technique (see section 4), with a 377.8 nm pulse laser used as the excitation wavelength. The emission was monitored at 750 nm.

5. Computational Section

The ground-state and excited-state geometries are optimized by density functional theory (DFT) and time-dependent DFT (TD-DFT), respectively, under B3LYP/6-311++g(3df,3pd) for all title compounds. The solvent effects were considered using the polarizable continuum model (PCM) with toluene (ε=2.38). The energy minima are confirmed with no imaginary frequencies by vibrational frequency analyses.

Table S5. The computed optical excitations and molecular orbital contributions for all title compounds.

Compound	Structure	State	E (eV)	Wavelength (nm)	f	Contribution	weight
H-NTF	Normal @S_0-opt	S_1	3.20	387.3	0.2805	HOMO→LUMO	98%
		S_2	3.65	339.8	0.1462	HOMO-1→LUMO	95%
	Normal @S_1-opt	S_1	2.62	473.4	0.2612	HOMO→LUMO	99%
		S_2	3.13	396.0	0.1714	HOMO-1→LUMO	98%
	Tautomer @S_0-opt	S_1	2.11	586.4	0.1768	HOMO→LUMO	99%
		S_2	2.48	499.6	0.0012	HOMO-1→LUMO	99%
	Tautomer @S_1-opt	S_1	1.57	787.3	0.0879	HOMO→LUMO	93%
		S_2	1.81	683.5	0.0151	HOMO-1→LUMO	93%
F-NTF	Normal @S_0-opt	S_1	3.04	408.0	0.2527	HOMO→LUMO	98%
		S_2	3.48	356.0	0.1401	HOMO-1→LUMO	96%
	Normal @S_1-opt	S_1	2.44	508.1	0.2192	HOMO→LUMO	99%
		S_2	3.00	413.9	0.1942	HOMO-1→LUMO	98%
	Tautomer @S_0-opt	S_1	2.08	595.6	0.1938	HOMO→LUMO	99%
		S_2	2.43	509.2	0.0015	HOMO-1→LUMO	99%
	Tautomer @S_1-opt	S_1	1.53	810.9	0.0911	HOMO→LUMO	92%
		S_2	1.76	703.0	0.0158	HOMO-1→LUMO	92%
O-NTF	Normal @S_0-opt	S_1	3.26	380.7	0.3941	HOMO→LUMO	99%
		S_2	3.72	333.7	0.1578	HOMO-1→LUMO	94%
	Normal @S_1-opt	S_1	2.72	456.2	0.4323	HOMO→LUMO	99%
		S_2	3.29	376.3	0.1593	HOMO-1→LUMO	97%
	Tautomer @S_0-opt	S_1	2.12	584.0	0.2332	HOMO→LUMO	99%
		S_2	2.52	491.5	0.0008	HOMO-1→LUMO	99%
	Tautomer @S_1-opt	S_1	1.66	747.6	0.1308	HOMO→LUMO	96%
		S_2	1.91	647.9	0.0083	HOMO-1→LUMO	96%
N-NTF	Normal @S_0-opt	S_1	3.07	403.4	0.7029	HOMO→LUMO	98%
		S_2	3.68	337.0	0.0984	HOMO-1→LUMO	95%
	Tautomer @S_0-opt	S_1	2.11	588.6	0.41	HOMO→LUMO	98%
		S_2	2.56	484.1	0.0008	HOMO-1→LUMO	99%
	Tautomer @S_1-opt	S_1	1.72	722.2	0.2396	HOMO→LUMO	97%
		S_2	2.03	609.7	0.0082	HOMO-1→LUMO	98%

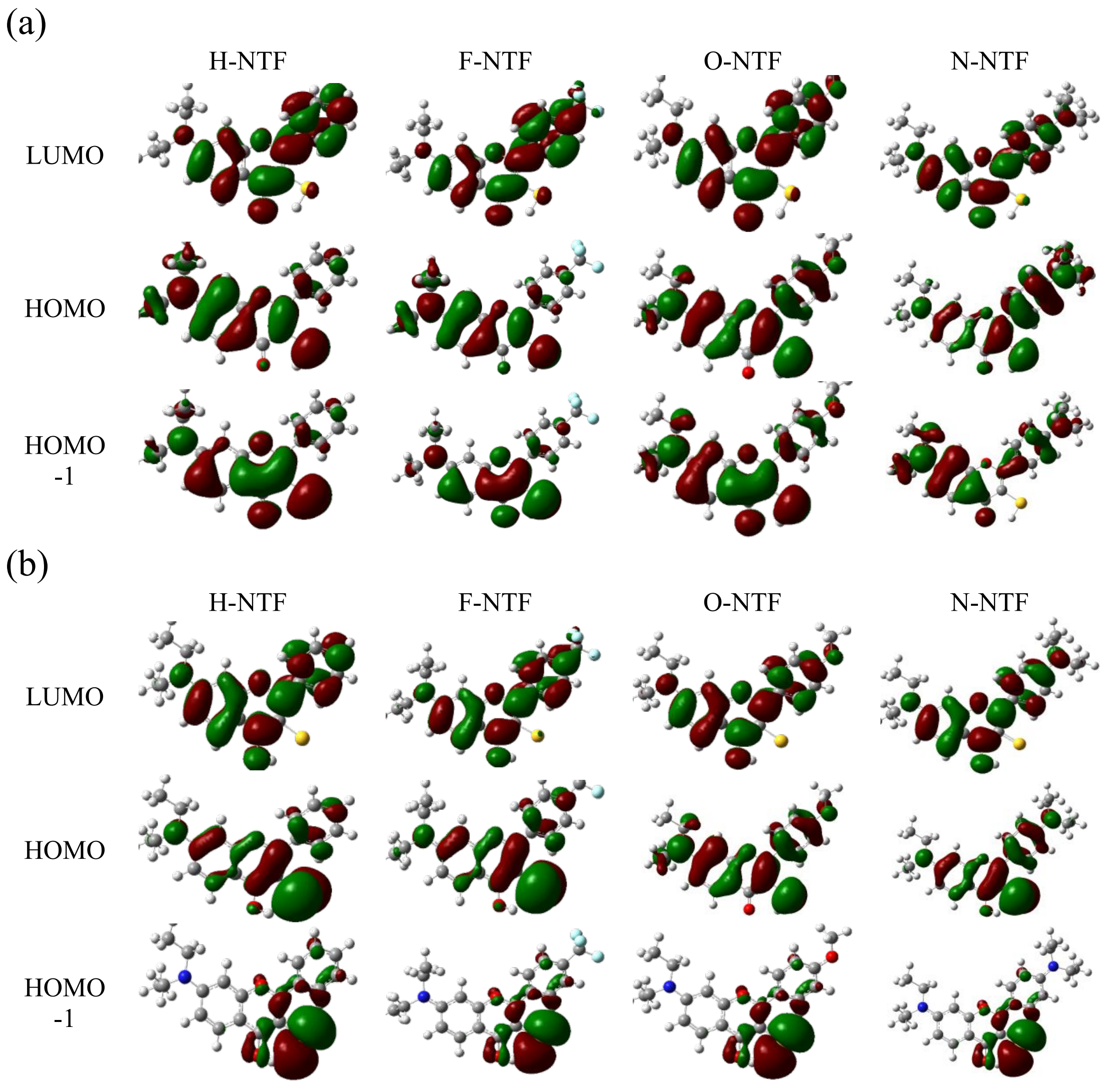

Figure S57. Frontier molecular orbitals associated with major optical transitions of (a) normal form at S_0-optimized structure and (b) tautomer form at S_1-optimized structure for all title compounds.

Table S6. The distances (Å) between S-H, H…O and C=O in S_0 and S_1-optimized geometries of normal forms.

	F-NTF		H-NTF		O-NTF		N-NTF	
	N@S_0	N@S_1	N@S_0	N@S_1	N@S_0	N@S_1	N@S_0	N@S_1
S-H	1.352	1.353	1.352	1.359	1.351	1.379	1.351	--
H…O	2.028	2.042	2.040	1.985	2.042	1.829	2.036	--
O=C	1.233	1.248	1.234	1.256	1.234	1.264	1.236	--

Table S7. The Cartesian coordinates of the normal forms and tautomers of S_0 and S_1-optimized **F-NTF**, **H-NTF**, **O-NTF** and **N-NTF**.

(a) F-NTF

Normal@S_0-opt				Normal@S_1-opt			
C	-4.379349	0.875414	-0.090953	C	4.387285	0.886912	-0.165089
C	-3.954291	-0.484543	0.003419	C	3.953107	-0.459151	-0.039497
C	-2.569397	-0.723169	0.040278	C	2.561611	-0.708699	0.089196
C	-1.67843	0.337267	-0.010338	C	1.660909	0.348737	0.087007
C	-2.088101	1.668755	-0.103818	C	2.085889	1.664097	-0.029988
C	-3.474624	1.901858	-0.144086	C	3.464633	1.905227	-0.153928
H	-5.43021	1.112978	-0.104238	H	5.430845	1.121529	-0.286354
H	-2.1609	-1.718484	0.091164	H	2.168467	-1.70471	0.200095
H	-3.816333	2.924806	-0.211095	H	3.78252	2.934228	-0.240233
O	-0.357393	0.015579	0.031357	O	0.360404	0.016441	0.197102
C	0.60811	0.97844	-0.008552	C	-0.639179	0.976601	0.115363
C	-1.112606	2.736132	-0.163171	C	1.092596	2.757793	0.058631
C	0.300287	2.299595	-0.103986	C	-0.266008	2.338749	0.148746
O	-1.416083	3.926677	-0.269866	O	1.475799	3.944343	0.104463
C	1.947979	0.364871	0.023889	C	-1.931672	0.380554	0.049904
C	2.167774	-0.84524	-0.647078	C	-2.119946	-0.983083	0.432119
C	3.416085	-1.443132	-0.636758	C	-3.356361	-1.580915	0.376472
C	4.465869	-0.847516	0.057692	C	-4.479895	-0.86986	-0.071531
C	4.257758	0.343301	0.74367	C	-4.315131	0.461721	-0.48135
C	3.008423	0.94562	0.724122	C	-3.08355	1.072171	-0.423967
H	1.357594	-1.311277	-1.187139	H	-1.279568	-1.552584	0.795563
H	3.574656	-2.36714	-1.173568	H	-3.463022	-2.609272	0.692706
H	5.065254	0.802365	1.294636	H	-5.162393	1.017655	-0.857042
H	2.858311	1.860593	1.276214	H	-2.996073	2.083944	-0.785498
N	-4.860047	-1.506887	0.059268	N	4.833056	-1.50356	-0.033726
C	-6.297963	-1.285493	-0.086283	C	6.281782	-1.291787	-0.04823
H	-6.472288	-0.486787	-0.805279	H	6.515963	-0.39437	0.517299
H	-6.717541	-2.185882	-0.535754	H	6.727997	-2.129471	0.485521
C	-7.014703	-0.988694	1.231456	C	6.857019	-1.212442	-1.465236
H	-6.634172	-0.078828	1.694144	H	6.433699	-0.375755	-2.017629
H	-8.084047	-0.862348	1.057376	H	7.936258	-1.077329	-1.405871
H	-6.882915	-1.806938	1.939919	H	6.659912	-2.127737	-2.022099
C	-4.444162	-2.89199	0.266051	C	4.405013	-2.905114	-0.015019
H	-5.257716	-3.400098	0.784271	H	5.170903	-3.471421	-0.542418
H	-3.595125	-2.915634	0.948236	H	3.489208	-3.011376	-0.589384
C	-4.115166	-3.638914	-1.027569	C	4.234091	-3.459268	1.401553
H	-3.828809	-4.66755	-0.804946	H	3.959756	-4.511875	1.344313

H	-4.979048	-3.665831	-1.692117	H	5.159811	-3.379991	1.969962
H	-3.293978	-3.164255	-1.563196	H	3.453571	-2.927658	1.943012
S	1.498534	3.587181	-0.234446	S	-1.434283	3.632187	0.423057
H	0.522368	4.510155	-0.387484	H	-0.444131	4.553908	0.436761
C	5.806107	-1.526663	0.10868	C	-5.797209	-1.545217	-0.19237
F	6.090269	-2.181128	-1.034726	F	-5.990322	-2.491973	0.756617
F	5.864255	-2.448	1.099828	F	-5.958746	-2.195655	-1.384812
F	6.813131	-0.66198	0.330809	F	-6.83828	-0.687507	-0.107448

Tautomer@S_0-opt				Tautomer @S_1-opt			
C	-4.363659	0.8404502	-0.085	C	-4.386833	0.877747	-0.05976
C	-3.91258	-0.520684	-0.006843	C	-3.973804	-0.474369	0.004572
C	-2.525098	-0.74197	0.02401	C	-2.57801	-0.721826	0.044575
C	-1.650233	0.3306271	-0.017136	C	-1.686309	0.324447	0.027625
C	-2.088663	1.6639992	-0.093895	C	-2.087541	1.668476	-0.043917
C	-3.487483	1.8827932	-0.127373	C	-3.468846	1.91027	-0.087952
H	-5.4191	1.0555842	-0.093123	H	-5.435128	1.126018	-0.072984
H	-2.103451	-1.731984	0.061561	H	-2.177846	-1.721131	0.080799
H	-3.854265	2.8972413	-0.17915	H	-3.816778	2.931318	-0.139557
O	-0.335304	0.0580361	0.014186	O	-0.356706	-0.002875	0.099776
C	0.636864	1.0198641	-0.019211	C	0.633044	0.943017	0.0072
C	-1.112022	2.6669613	-0.131872	C	-1.070953	2.659483	-0.08874
C	0.300854	2.3648002	-0.088922	C	0.281321	2.284668	-0.106156
O	-1.463482	3.9213614	-0.216597	O	-1.457922	3.949042	-0.185561
C	1.9632341	0.4007681	0.003541	C	1.950095	0.356378	0.051237
C	2.1031321	-0.957725	-0.339063	C	2.150351	-0.951995	-0.447107
C	3.3396482	-1.57372	-0.323115	C	3.401866	-1.528639	-0.448259
C	4.4739183	-0.853029	0.046877	C	4.501494	-0.829823	0.061603
C	4.3544113	0.485459	0.398748	C	4.319991	0.444097	0.592168
C	3.1159362	1.1094421	0.371405	C	3.062383	1.022145	0.606278
H	1.236845	-1.529268	-0.630996	H	1.314245	-1.494174	-0.861347
H	3.4242642	-2.613231	-0.605786	H	3.53418	-2.520421	-0.857023
H	5.2271394	1.0506051	0.691024	H	5.156165	0.977455	1.019999
H	3.0372872	2.1506452	0.635284	H	2.932828	1.974698	1.094401
N	-4.803651	-1.548051	0.040704	N	-4.874232	-1.505408	0.031196
C	-6.248137	-1.346579	-0.094579	C	-6.310424	-1.279451	-0.110412
H	-6.439111	-0.540678	-0.800323	H	-6.480922	-0.464317	-0.812227
H	-6.652529	-2.24709	-0.5564	H	-6.732473	-2.169377	-0.578152
C	-6.959395	-1.084468	1.2325951	C	-7.027575	-1.007175	1.213004
H	-6.593802	-0.174496	1.7070331	H	-6.63968	-0.111758	1.697033
H	-8.03177	-0.976442	1.0667411	H	-8.095388	-0.86738	1.039636
H	-6.807471	-1.910778	1.9272761	H	-6.904635	-1.843008	1.90231

C	-4.37067	-2.933533	0.225691	C	-4.458512	-2.894579	0.206045
H	-5.176427	-3.455248	0.741562	H	-5.274279	-3.414842	0.708609
H	-3.51756	-2.956053	0.902098	H	-3.612484	-2.936863	0.890773
C	-4.043938	-3.655127	-1.082012	C	-4.125092	-3.611063	-1.103791
H	-3.745586	-4.683532	-0.876269	H	-3.843854	-4.645984	-0.904618
H	-4.912404	-3.681501	-1.740331	H	-4.985211	-3.618091	-1.773726
H	-3.231364	-3.16447	-1.616194	H	-3.298904	-3.126363	-1.622587
S	1.3338961	3.7589073	-0.16872	S	1.480221	3.553055	-0.374824
H	-0.572416	4.4166094	-0.235822	H	-0.657646	4.500542	-0.162265
C	5.8045004	-1.546698	0.107184	C	5.843966	-1.491567	0.102833
F	5.9657864	-2.429274	-0.901258	F	6.089236	-2.22151	-1.007235
F	5.9523504	-2.253971	1.2541501	F	5.961011	-2.35256	1.146992
F	6.8398104	-0.689759	0.050388	F	6.855003	-0.610166	0.225769

(b) H-NTF

Normal@S_0-opt				Normal@S_1-opt			
C	2.939656	-1.438704	-0.140302	C	2.934446	1.473935	-0.096284
C	2.917847	-0.01754	-0.007962	C	2.912128	0.058742	-0.019406
C	1.658133	0.601485	0.061954	C	1.648421	-0.585347	0.067571
C	0.502085	-0.162026	0.005076	C	0.48283	0.162692	0.071224
C	0.517478	-1.551527	-0.126092	C	0.501084	1.553044	0.003477
C	1.780126	-2.165772	-0.198502	C	1.75396	2.183203	-0.077359
H	3.879996	-1.963352	-0.179575	H	3.865952	2.006657	-0.185522
H	1.548415	1.669899	0.143469	H	1.559128	-1.655611	0.144234
H	1.816436	-3.241567	-0.295075	H	1.765509	3.262721	-0.124671
O	-0.672805	0.518512	0.080837	O	-0.669762	-0.543769	0.141565
C	-1.872437	-0.129974	0.033589	C	-1.900222	0.083149	0.04133
C	-0.72237	-2.297749	-0.192227	C	-0.760529	2.302071	0.107986
C	-1.949371	-1.481538	-0.100135	C	-1.934794	1.496305	0.147084
O	-0.766341	-3.522957	-0.33236	O	-0.759888	3.552712	0.220348
C	-2.986026	0.832644	0.108344	C	-2.968276	-0.845848	-0.118224
C	-2.882993	2.060508	-0.558255	C	-2.78565	-2.221592	0.213431
C	-3.918293	2.981668	-0.502423	C	-3.812784	-3.132032	0.075529
C	-5.066949	2.700889	0.231043	C	-5.064106	-2.740547	-0.410487
C	-5.171118	1.492542	0.909776	C	-5.255498	-1.403473	-0.770146
C	-4.140784	0.563237	0.848774	C	-4.241311	-0.476423	-0.638885
H	-1.992031	2.284657	-1.126411	H	-1.834231	-2.548861	0.603069
H	-3.829029	3.919824	-1.033147	H	-3.643165	-4.164549	0.353471
H	-6.053657	1.272022	1.49457	H	-6.206536	-1.0865	-1.178304
H	-4.227039	-0.363332	1.39642	H	-4.412326	0.533662	-0.976372
H	-5.872819	3.420797	0.275685	H	-5.862897	-3.460522	-0.519985
N	4.077479	0.706292	0.052112	N	4.05895	-0.684351	-0.019377
C	5.391091	0.091171	-0.128489	C	5.380603	-0.057651	0.013018
H	5.322501	-0.702358	-0.870924	H	5.332701	0.842516	0.620533
H	6.043837	0.848547	-0.563448	H	6.04732	-0.748413	0.527502
C	6.011246	-0.436909	1.165823	C	5.932625	0.254136	-1.380424
H	5.39288	-1.213793	1.613893	H	5.289919	0.952752	-1.913047
H	6.997496	-0.85786	0.965791	H	6.922998	0.698956	-1.286569
H	6.1282	0.363271	1.89716	H	6.023587	-0.651853	-1.978852
C	4.071881	2.146372	0.295293	C	4.055294	-2.148211	-0.049367
H	5.005015	2.393171	0.802295	H	4.962408	-2.454534	-0.568971
H	3.276609	2.390137	0.99896	H	3.221862	-2.495211	-0.654568
C	3.94126	2.988968	-0.974827	C	4.01925	-2.781222	1.343736
H	3.958354	4.050628	-0.725038	H	4.059731	-3.866217	1.250494
H	4.765681	2.790483	-1.660148	H	4.870182	-2.460551	1.94384
H	3.010225	2.776597	-1.498805	H	3.107376	-2.514845	1.875777

S	-3.469971	-2.367857	-0.245537	S	-3.419342	2.371496	0.476971
H	-2.803487	-3.527006	-0.442422	H	-2.720306	3.535796	0.533919

Tautomer@S_0-opt				Tautomer @S_1-opt			
C	2.8180123	-1.543084	-0.232476	C	2.848716	-1.538106	-0.202392
C	2.8420403	-0.108923	-0.185975	C	2.875542	-0.124145	-0.187056
C	1.6051442	0.550742	-0.101218	C	1.625459	0.538859	-0.102232
C	0.4248771	-0.178357	-0.101104	C	0.451419	-0.180442	-0.069281
C	0.3993941	-1.579551	-0.163885	C	0.418419	-1.582678	-0.111605
C	1.6478652	-2.242818	-0.228013	C	1.656957	-2.238191	-0.175856
H	3.7405123	-2.096437	-0.276345	H	3.764822	-2.102748	-0.242928
H	1.5259482	1.6209921	-0.024005	H	1.549103	1.610532	-0.038922
H	1.6625971	-3.321863	-0.271054	H	1.673143	-3.317724	-0.202912
O	-0.725791	0.5115761	-0.021133	O	-0.70515	0.543914	0.043969
C	-1.957777	-0.077151	-0.001719	C	-1.946929	-0.054405	0.011606
C	-0.855993	-2.206733	-0.152174	C	-0.854783	-2.214977	-0.096768
C	-2.083562	-1.459062	-0.067128	C	-2.023937	-1.439875	-0.083153
O	-0.938393	-3.508605	-0.229106	O	-0.88158	-3.564665	-0.162755
C	-3.009087	0.9398431	0.064823	C	-3.017607	0.910077	0.099493
C	-2.738562	2.2492642	-0.372658	C	-2.834168	2.21258	-0.41623
C	-3.711754	3.2328283	-0.312968	C	-3.853139	3.145635	-0.366829
C	-4.973891	2.9418933	0.197571	C	-5.083302	2.824718	0.208619
C	-5.250379	1.6554442	0.6440741	C	-5.269885	1.557857	0.752282
C	-4.28545	0.6599091	0.5745761	C	-4.250563	0.618851	0.718682
H	-1.764887	2.4892862	-0.771054	H	-1.892736	2.468974	-0.878607
H	-3.486005	4.2292714	-0.668119	H	-3.69336	4.130491	-0.785511
H	-6.225281	1.4194422	1.0486811	H	-6.205785	1.307138	1.233487
H	-4.512775	-0.337119	0.9138171	H	-4.390607	-0.328198	1.216551
H	-5.732872	3.7111374	0.244433	H	-5.875677	3.559412	0.249588
N	4.0242864	0.581816	-0.236856	N	4.055167	0.587393	-0.264077
C	5.3000534	-0.102707	-0.011812	C	5.327616	-0.084168	-0.001831
H	5.3141035	-1.024951	-0.587199	H	5.346872	-1.025978	-0.545316
H	6.0860335	0.5118159	-0.438332	H	6.119675	0.515586	-0.439655
C	5.6025595	-0.384613	1.4607001	C	5.617297	-0.320464	1.482471
H	4.8410484	-1.023033	1.9078122	H	4.845979	-0.937241	1.942625
H	6.5657385	-0.887444	1.5549391	H	6.575403	-0.828169	1.601848
H	5.6477235	0.54015	2.0364412	H	5.666495	0.622407	2.028148
C	3.9798084	2.0485881	-0.134624	C	4.003312	2.051306	-0.162761
H	3.6502464	2.3434331	0.8687551	H	3.711302	2.355493	0.850651
H	3.2178023	2.3984391	-0.830394	H	3.216318	2.396787	-0.831359
C	5.2780215	2.7691491	-0.472216	C	5.285355	2.775081	-0.555107
H	5.0812735	3.8408412	-0.45953	H	5.084171	3.84619	-0.55582

H	6.0715226	2.5765611	0.247941	H	6.103657	2.599513	0.14146
H	5.6375575	2.5095531	-1.46777	H	5.613557	2.496734	-1.556649
S	-3.526325	-2.431447	-0.098678	S	-3.559288	-2.288546	-0.296207
H	-1.943532	-3.68137	-0.212911	H	-1.810729	-3.84328	-0.100833

(c) O-NTF

Normal@S_0-opt				Normal@S_1-opt			
C	-3.687881	1.175468	-0.256875	C	-3.687881	1.175468	-0.256875
C	-3.412766	-0.223445	-0.193049	C	-3.412766	-0.223445	-0.193049
C	-2.064235	-0.603377	-0.094437	C	-2.064235	-0.603377	-0.094437
C	-1.063024	0.359249	-0.098755	C	-1.063024	0.359249	-0.098755
C	-1.327551	1.724864	-0.179433	C	-1.327551	1.724864	-0.179433
C	-2.679328	2.102572	-0.256431	C	-2.679328	2.102572	-0.256431
H	-4.70416	1.527531	-0.312172	H	-4.70416	1.527531	-0.312172
H	-1.760529	-1.631697	-0.001438	H	-1.760529	-1.631697	-0.001438
H	-2.909585	3.156768	-0.315258	H	-2.909585	3.156768	-0.315258
O	0.2124	-0.10064	-0.000789	O	0.2124	-0.10064	-0.000789
C	1.278148	0.752777	0.00936	C	1.278148	0.752777	0.00936
C	-0.240373	2.684929	-0.186316	C	-0.240373	2.684929	-0.186316
C	1.108764	2.101294	-0.086257	C	1.108764	2.101294	-0.086257
O	-0.419272	3.902139	-0.287489	O	-0.419272	3.902139	-0.287489
C	2.540514	0.005544	0.097798	C	2.540514	0.005544	0.097798
C	2.66962	-1.223306	-0.555517	C	2.66962	-1.223306	-0.555517
C	3.84569	-1.957768	-0.495107	C	3.84569	-1.957768	-0.495107
C	4.925844	-1.47483	0.245665	C	4.925844	-1.47483	0.245665
C	4.804121	-0.257062	0.921137	C	4.804121	-0.257062	0.921137
C	3.632641	0.469863	0.844483	C	3.632641	0.469863	0.844483
H	1.840667	-1.610329	-1.129895	H	1.840667	-1.610329	-1.129895
H	3.909328	-2.895063	-1.025384	H	3.909328	-2.895063	-1.025384
H	5.639369	0.099861	1.506894	H	5.639369	0.099861	1.506894
H	3.558338	1.397637	1.391577	H	3.558338	1.397637	1.391577
N	-4.425026	-1.157218	-0.244281	N	-4.425026	-1.157218	-0.244281
C	-5.808334	-0.755832	0.016476	C	-5.808334	-0.755832	0.016476
H	-6.028731	0.143879	-0.552871	H	-6.028731	0.143879	-0.552871
H	-6.460323	-1.520297	-0.394804	H	-6.460323	-1.520297	-0.394804
C	-6.130885	-0.541332	1.496825	C	-6.130885	-0.541332	1.496825
H	-5.507397	0.240805	1.929133	H	-5.507397	0.240805	1.929133
H	-7.174757	-0.247493	1.614863	H	-7.174757	-0.247493	1.614863
H	-5.972146	-1.45506	2.070468	H	-5.972146	-1.45506	2.070468
C	-4.070508	-2.575262	-0.10579	C	-4.070508	-2.575262	-0.10579
H	-3.710317	-2.781888	0.910183	H	-3.710317	-2.781888	0.910183
H	-3.234865	-2.767597	-0.777744	H	-3.234865	-2.767597	-0.777744
C	-5.177647	-3.56036	-0.458567	C	-5.177647	-3.56036	-0.458567
H	-4.758114	-4.565916	-0.43481	H	-4.758114	-4.565916	-0.43481
H	-6.007342	-3.538695	0.246202	H	-6.007342	-3.538695	0.246202
H	-5.566905	-3.385323	-1.461589	H	-5.566905	-3.385323	-1.461589
S	2.44705	3.252438	-0.170047	S	2.44705	3.252438	-0.170047

H	1.586554	4.278385	-0.351775	H	1.586554	4.278385	-0.351775
O	6.116086	-2.11073	0.371478	O	6.116086	-2.11073	0.371478
C	6.305317	-3.353353	-0.294593	C	6.305317	-3.353353	-0.294593
H	6.20327	-3.241713	-1.375707	H	6.20327	-3.241713	-1.375707
H	7.31759	-3.666611	-0.058276	H	7.31759	-3.666611	-0.058276
H	5.599966	-4.105596	0.063824	H	5.599966	-4.105596	0.063824

Tautomer@S$_0$-opt				Tautomer @S$_1$-opt			
C	-3.66581	1.1555351	-0.224604	C	-3.694409	1.164636	-0.184841
C	-3.37055	-0.247033	-0.183399	C	-3.414885	-0.221336	-0.183185
C	-2.01785	-0.614991	-0.098627	C	-2.049648	-0.599665	-0.113269
C	-1.029345	0.358641	-0.09608	C	-1.05744	0.354947	-0.081524
C	-1.315806	1.7292461	-0.155189	C	-1.329164	1.731974	-0.10979
C	-2.679459	2.0983192	-0.217061	C	-2.681032	2.105131	-0.158919
H	-4.688148	1.4907221	-0.265983	H	-4.711058	1.51873	-0.211751
H	-1.702952	-1.641171	-0.024781	H	-1.743759	-1.630253	-0.062478
H	-2.933719	3.1472352	-0.256338	H	-2.929556	3.15604	-0.173655
O	0.247088	-0.058286	-0.017568	O	0.228135	-0.104683	0.012492
C	1.3195961	0.7875681	-0.003063	C	1.316346	0.748244	-0.003071
C	-0.228576	2.6195932	-0.144624	C	-0.223213	2.622999	-0.099469
C	1.1308231	2.1653322	-0.066699	C	1.08899	2.119293	-0.090084
O	-0.441762	3.9084663	-0.220518	O	-0.487274	3.946127	-0.159276
C	2.5659422	0.031851	0.058046	C	2.561898	0.025706	0.087036
C	2.5840082	-1.321295	-0.316555	C	2.651549	-1.288489	-0.413212
C	3.7437303	-2.077428	-0.268076	C	3.831006	-2.012677	-0.359952
C	4.9331084	-1.495133	0.17711	C	4.971689	-1.448161	0.217474
C	4.9318534	-0.153149	0.566717	C	4.896179	-0.158983	0.75438
C	3.7760053	0.597342	0.502104	C	3.718256	0.555041	0.702775
H	1.6773151	-1.791592	-0.664194	H	1.785359	-1.737138	-0.875863
H	3.7123393	-3.109732	-0.579848	H	3.854252	-3.008611	-0.775095
H	5.8545474	0.287975	0.9168041	H	5.770517	0.255399	1.236712
H	3.7977553	1.6330751	0.7982201	H	3.677662	1.522407	1.177671
N	-4.370058	-1.18468	-0.23968	N	-4.413348	-1.170858	-0.259158
C	-5.766539	-0.799434	-0.022835	C	-5.801492	-0.787241	-0.006434
H	-5.980014	0.096817	-0.600162	H	-6.017192	0.12873	-0.551729
H	-6.394978	-1.57239	-0.453157	H	-6.444227	-1.542396	-0.448655
C	-6.134768	-0.589988	1.4470241	C	-6.145915	-0.618061	1.475484
H	-5.53518	0.199985	1.8984711	H	-5.527438	0.148943	1.940431
H	-7.185408	-0.310179	1.5331231	H	-7.191213	-0.326607	1.587043
H	-5.980992	-1.501825	2.0246621	H	-5.996792	-1.549731	2.022227
C	-4.001616	-2.604134	-0.132007	C	-4.049133	-2.589275	-0.153113
H	-3.620856	-2.817277	0.8741831	H	-3.696727	-2.819202	0.860728

H	-3.176692	-2.778446	-0.822269	H	-3.207874	-2.762079	-0.822547
C	-5.10549	-3.595411	-0.474786	C	-5.147219	-3.572676	-0.538355
H	-4.676522	-4.596968	-0.457308	H	-4.721611	-4.575972	-0.533182
H	-5.926824	-3.581809	0.239768	H	-5.98346	-3.571694	0.15902
H	-5.507009	-3.42369	-1.473331	H	-5.528305	-3.377412	-1.540795
S	2.3228752	3.4364263	-0.104366	S	2.392609	3.287469	-0.307226
H	0.497738	4.3023463	-0.208858	H	0.368299	4.410238	-0.132031
O	6.1187445	-2.144141	0.263019	O	6.173076	-2.073188	0.323313
C	6.1857625	-3.510859	-0.125778	C	6.303147	-3.395639	-0.180131
H	5.9192115	-3.636145	-1.176958	H	6.113016	-3.430744	-1.254872
H	7.2180475	-3.812376	0.022992	H	7.330434	-3.689552	0.013392
H	5.5340764	-4.130355	0.493419	H	5.626799	-4.083233	0.332114

(d) N-NTF

Normal@S_0-opt			
C	-4.664518	0.876292	-0.205896
C	-4.210495	-0.474101	-0.137882
C	-2.822603	-0.677329	-0.080341
C	-1.952712	0.405348	-0.125379
C	-2.392635	1.723957	-0.210139
C	-3.782869	1.924984	-0.247135
H	-5.718874	1.094693	-0.233481
H	-2.387006	-1.657118	0.013196
H	-4.148405	2.94009	-0.309749
O	-0.627685	0.113424	-0.061414
C	0.325474	1.092949	-0.095282
C	-1.435078	2.813887	-0.263868
C	-0.024023	2.409194	-0.203793
O	-1.774646	3.997168	-0.375062
C	1.666632	0.512588	-0.032157
C	1.920348	-0.746544	-0.596653
C	3.172075	-1.324997	-0.558287
C	4.262188	-0.684187	0.076014
C	3.994261	0.571387	0.666534
C	2.740428	1.147036	0.60418
H	1.120655	-1.275427	-1.094639
H	3.307102	-2.278162	-1.043942
H	4.767272	1.098762	1.201906
H	2.592046	2.096104	1.097244
N	-5.097362	-1.531746	-0.148769
C	-6.502953	-1.310733	0.194848
H	-6.869574	-0.44596	-0.352539
H	-7.0751	-2.151958	-0.184489
C	-6.762991	-1.140183	1.693216
H	-6.219769	-0.28488	2.094198
H	-7.827009	-0.981973	1.87446
H	-6.455859	-2.026461	2.249527
C	-4.557369	-2.890444	-0.021989
H	-4.138364	-3.046092	0.9807
H	-3.727575	-2.97513	-0.722623
C	-5.539759	-4.011429	-0.337825
H	-4.992999	-4.954248	-0.335912
H	-6.337716	-4.098441	0.397957
H	-5.986914	-3.887062	-1.324208
S	1.150174	3.723203	-0.356502

H	0.156895	4.622506	-0.532825
N	5.510483	-1.252884	0.119315
C	5.764767	-2.596357	-0.390167
H	6.590773	-3.012376	0.187873
H	4.905507	-3.232633	-0.180353
C	6.111246	-2.641236	-1.879872
H	5.296497	-2.250479	-2.488282
H	7.001682	-2.048134	-2.090355
H	6.308666	-3.66822	-2.19083
C	6.661632	-0.543616	0.668723
H	7.546926	-0.90324	0.142839
H	6.582428	0.515951	0.42787
C	6.849949	-0.736162	2.1749
H	7.735077	-0.197442	2.516333
H	5.98976	-0.367392	2.732388
H	6.981925	-1.79066	2.419243

Tautomer@S_0-opt				Tautomer @S_1-opt			
C	-4.641456	0.856912	-0.193372	C	-4.671828	0.877899	-0.115523
C	-4.168267	-0.493086	-0.137109	C	-4.223534	-0.463332	-0.118781
C	-2.778241	-0.682865	-0.08475	C	-2.821093	-0.67146	-0.105883
C	-1.922836	0.409642	-0.125026	C	-1.950566	0.397005	-0.122095
C	-2.383572	1.728943	-0.198582	C	-2.391944	1.73012	-0.146926
C	-3.782861	1.919184	-0.228801	C	-3.781209	1.934878	-0.13938
H	-5.699059	1.058244	-0.21559	H	-5.724631	1.104277	-0.101861
H	-2.332256	-1.658616	-0.001499	H	-2.389815	-1.656595	-0.062564
H	-4.171103	2.925752	-0.27995	H	-4.156713	2.947461	-0.150031
O	-0.601397	0.160755	-0.07329	O	-0.619227	0.098656	-0.084761
C	0.359618	1.132953	-0.101805	C	0.361884	1.079642	-0.125895
C	-1.415076	2.751079	-0.233063	C	-1.405735	2.749609	-0.195548
C	-0.013704	2.478378	-0.187082	C	-0.040151	2.416463	-0.228996
O	-1.801851	4.001965	-0.322082	O	-1.834211	4.027442	-0.26444
C	1.682957	0.543697	-0.050477	C	1.679295	0.516519	-0.039133
C	1.856496	-0.840602	-0.260407	C	1.917068	-0.815819	-0.448965
C	3.093807	-1.440691	-0.215856	C	3.169698	-1.386601	-0.402098
C	4.26769	-0.69736	0.061156	C	4.292643	-0.675192	0.083558
C	4.091228	0.686853	0.28518	C	4.048787	0.641	0.53946
C	2.849433	1.282168	0.222438	C	2.793429	1.206837	0.486312
H	0.999596	-1.457564	-0.482064	H	1.098977	-1.397714	-0.846512
H	3.152929	-2.498025	-0.418941	H	3.282166	-2.393768	-0.771853
H	4.936309	1.311583	0.526337	H	4.843719	1.21731	0.985831
H	2.769535	2.342059	0.398811	H	2.662374	2.194427	0.89938

N	-5.042375	-1.555025	-0.152943	N	-5.101438	-1.52904	-0.148462
C	-6.457936	-1.352646	0.16363	C	-6.508884	-1.319694	0.18796
H	-6.825711	-0.491602	-0.388724	H	-6.86847	-0.437452	-0.33637
H	-7.010699	-2.200328	-0.22874	H	-7.078694	-2.148659	-0.220883
C	-6.746152	-1.191009	1.657381	C	-6.782456	-1.194066	1.688947
H	-6.223349	-0.329027	2.071032	H	-6.236085	-0.35642	2.121187
H	-7.815449	-1.049728	1.819715	H	-7.847103	-1.033326	1.86447
H	-6.435293	-2.074403	2.215946	H	-6.487774	-2.100146	2.21951
C	-4.488947	-2.911918	-0.049081	C	-4.561237	-2.891307	-0.066387
H	-4.05835	-3.071226	0.947468	H	-4.140797	-3.078437	0.930359
H	-3.666001	-2.980293	-0.759698	H	-3.733999	-2.957311	-0.771186
C	-5.463317	-4.039401	-0.364565	C	-5.545323	-4.001817	-0.413334
H	-4.906813	-4.976366	-0.370522	H	-4.999162	-4.944706	-0.437523
H	-6.255297	-4.137785	0.376108	H	-6.343299	-4.108425	0.319861
H	-5.917421	-3.915822	-1.347717	H	-5.992284	-3.849893	-1.395949
S	1.004115	3.895076	-0.27772	S	1.083305	3.735546	-0.519251
H	-0.925604	4.516361	-0.342491	H	-1.036162	4.588188	-0.296096
N	5.502266	-1.288351	0.106591	N	5.545859	-1.239781	0.13078
C	5.675199	-2.732116	-0.023862	C	5.771428	-2.64522	-0.184406
H	6.567015	-3.004448	0.541626	H	6.627599	-2.978912	0.403826
H	4.845786	-3.24247	0.46419	H	4.923174	-3.234389	0.162886
C	5.821107	-3.211002	-1.469618	C	6.036911	-2.916643	-1.667404
H	4.935863	-2.971208	-2.057382	H	5.190291	-2.615109	-2.282944
H	6.680397	-2.742902	-1.950539	H	6.914706	-2.369585	-2.012596
H	5.967293	-4.291849	-1.495581	H	6.217268	-3.980485	-1.829669
C	6.724283	-0.508587	0.285455	C	6.726558	-0.463235	0.489848
H	7.528641	-1.046298	-0.217612	H	7.573589	-0.884609	-0.053997
H	6.626713	0.441419	-0.238064	H	6.612273	0.555896	0.122306
C	7.101755	-0.275851	1.749503	C	7.037716	-0.457229	1.988669
H	8.030471	0.292926	1.812637	H	7.941298	0.122572	2.183402
H	6.325515	0.278955	2.274903	H	6.220716	-0.019797	2.561206
H	7.250016	-1.222741	2.269583	H	7.20175	-1.470313	2.357196

6. References

(1) Wang, C.-H.; Liu, Z.-Y.; Huang, C.-H.; Chen, C.-T.; Meng, F.-Y.; Liao, Y.-C.; Liu, Y.-H.; Chang, C.-C.; Li, E. Y.; Chou, P.-T., Chapter Open for the Excited-State Intramolecular Thiol Proton Transfer in the Room-Temperature Solution. *J. Am. Chem. Soc.* **2021,** *143*, 12715-12724.

(2) Brewer, W. E.; Studer, S. L.; Standiford, M.; Chou, P. T., Dynamics of the triplet state and the reverse proton transfer of 3-hydroxyflavone. *J. Phys. Chem.* **1989,** *93*, 6088-6094.

(3) Giles, G. I.; Tasker, K. M.; Jacob, C., Hypothesis: the role of reactive sulfur species in oxidative stress. *Free Radical Biology and Medicine* **2001,** *31*, 1279-1283.

(4) Bates, R. G.; Paabo, M.; Robinson, R. A., INTERPRETATION OF pH MEASUREMENTS IN ALCOHOL—WATER SOLVENTS1. *J. Phys. Chem.* **1963,** *67*, 1833-1838.

(5) Wyman, J., The dielectric constant of mixtures of ethyl alcohol and water from-5 to 40. *J. Am. Chem. Soc.* **1931,** *53*, 3292-3301.

(6) Seybold, P. G.; Shields, G. C., Computational estimation of pKa values. *Wiley Interdisciplinary Reviews: Computational Molecular Science* **2015,** *5*, 290-297.

(7) Pezzola, S.; Tarallo, S.; Iannini, A.; Venanzi, M.; Galloni, P.; Conte, V.; Sabuzi, F., An Accurate Approach for Computational pKa Determination of Phenolic Compounds. *Molecules* **2022,** *27*, 8590.

(8) Burk, P.; Koppel, I. A.; Koppel, I.; Leito, I.; Travnikova, O., Critical test of performance of B3LYP functional for prediction of gas-phase acidities and basicities. *Chem. Phys. Lett.* **2000,** *323*, 482-489.
